# Novel Bioconjugate Materials: Synthesis, Characterization and Medical Applications

**DOI:** 10.1002/adhm.202500303

**Published:** 2025-10-23

**Authors:** Ellie Martin, Sean P. Doidge, Eiman Aleem, Suela Kellici, Steven Dunn, Claire Atkinson, Philip D. Howes

**Affiliations:** ^1^ College of Technology and Environment School of Engineering and Design London South Bank University 103 Borough Road London SE1 0AA UK; ^2^ Cancer, Infection and Therapeutics Research Group College of Health and Life Science School of Applied and Health Sciences London South Bank University 103 Borough Road London SE1 0AA UK; ^3^ Materials and Environment Research Centre London South Bank University 103 Borough Road London SE1 0AA UK; ^4^ School of Engineering and Informatics Department of Engineering and Design University of Sussex Brighton BN1 9RH UK

**Keywords:** bio‐bio conjugation, bioconjugation, diagnostics, nanozymes, novel materials, targeted therapy

## Abstract

Bioconjugation is a pillar of modern medicine, enabling the likes of targeted therapeutics and sensitive diagnostics by exploiting synergies between biomolecules and functional materials. Conjugation techniques have expanded to match an evolving materials discovery landscape, fueling a new wave of bioconjugates. Despite the breadth of conjugate literature, most reviews describe common and relatively simple substrates such as metal nanoparticles or polymers. This review therefore centers around novel materials including biological (e.g., viral capsids, live cells), hybrid (e.g., gold‐decorated nanoparticles, covalent‐organic frameworks), and synthetic (e.g., piezoelectrics, upconverting nanoparticles) substrates. Applications in cancer and viral therapy, tissue engineering, optogenetics, antimicrobials, diagnostics, advanced imaging, and related topics are explored, revealing trends in conjugation approach. This review also compares characterization techniques used to confirm and optimize conjugation before offering perspectives on the future of the field. By shedding light on the latest materials, applications, and analytical methods, we hope to empower researchers to harness bioconjugation for transformative medical innovations.

## Graphical abstract

This review discusses the bioconjugation of novel materials grouped under “biological”, “hybrid” and “synthetic” categories. Medical applications ranging from cancer therapy and diagnostics to optogenetics and tissue engineering are explored, and conjugation trends are highlighted as a general guide. The review concludes with a comparison of techniques used to confirm and characterize conjugates before offering perspectives on the future of this rapidly‐developing field.

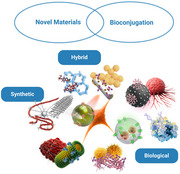

## 1 Introduction

Bioconjugation is an exciting area of research that has transformed modern medicine, enabling the likes of targeted drugs; responsive therapeutics; and prolonged diagnostics. Since the term's introduction in the 1980s,^[^ ^1^, ^2^ ^]^ building on work from the 1950s,^[^ ^3^ ^]^ bioconjugation has exploded across disciplines. Indeed, papers referencing “bioconjugation” have increased nearly twelvefold in the last two decades.^[^ ^4^ ^]^ This often owes to the fact that conjugation joins the properties and scale economies of materials science with the specificity, biocompatibility and dynamic nature of biomolecules. It enhances functionality while maintaining individual nano‐ and micro‐structures, occupying a sweet spot between performance and simplicity. Altogether, bioconjugation is a strong tool for enhancing even the most unique materials.

While inorganic particles are often used for conjugation, the term is not limited by substrate. Bioconjugation is usually defined as a stable linkage between two molecules, at least one of which is a biomolecule or derivative.^[^ ^5^ ^]^ This covers a range of interactions with a frequent emphasis on covalent bonding. Synthetic ligands such as polyethylene glycol (PEG) are sometimes considered honorary “biomolecules”, but for simplicity we will only consider ligands with at least one true biomolecule or derivative. This is because ligands are sometimes multi‐component, with some parts assisting the attachment itself, and other parts providing the key functionality of the ligand.

**Figure 1:**
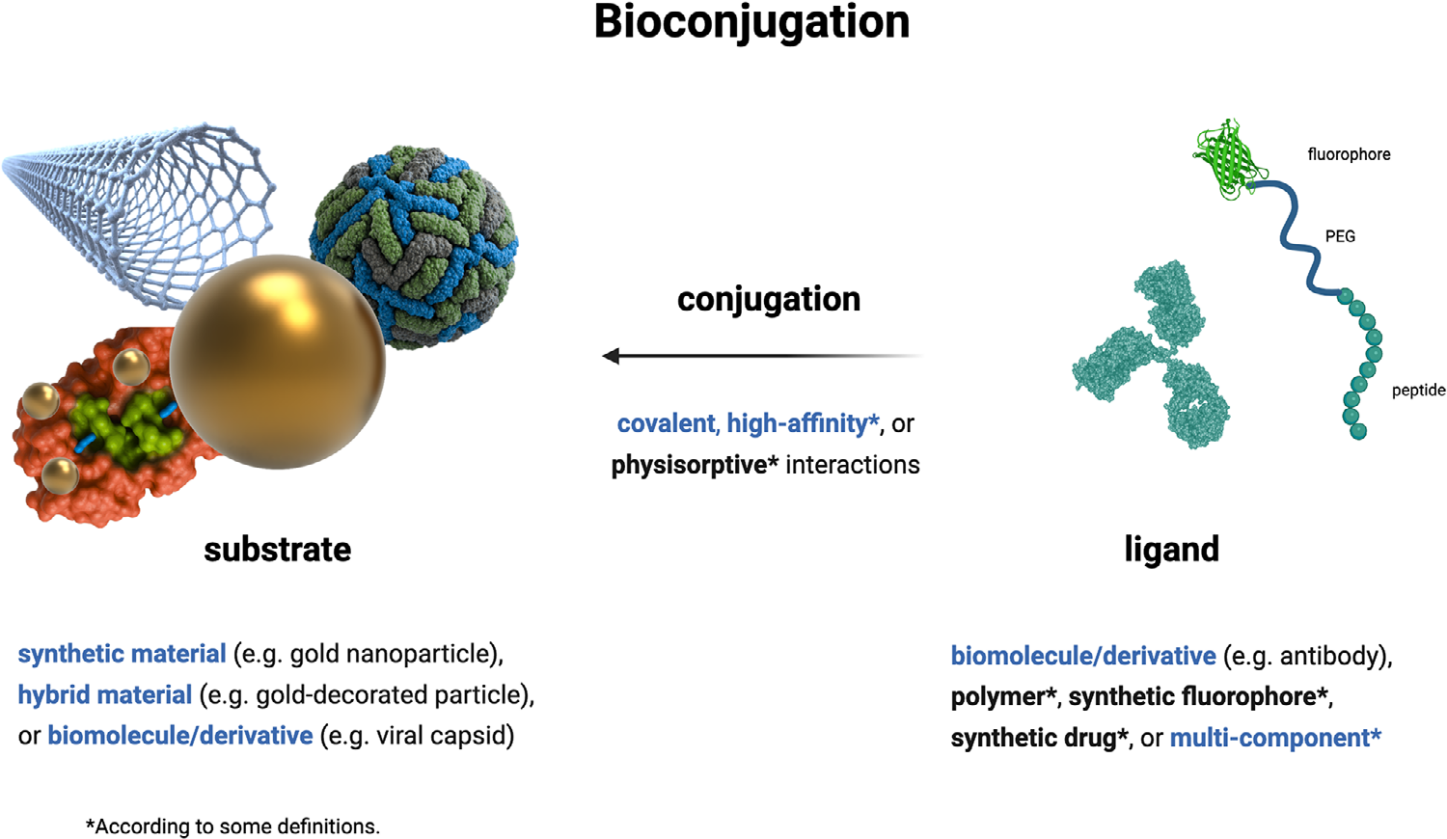
Visual representation of the terms bioconjugation, ligand and substrate. Categories for each are specified in writing; this review focuses on those highlighted in blue. Created in Martin, C. (2025) https://BioRender.com/p19o072.

Biomolecules can attach t most surfaces with the right chemistry, and as such, two groups of substrates are often left out of reviews. The first is biomolecules, in what is termed “bio‐bio conjugation”. The second is hybrid materials presenting a surface of two or more synthetic materials, or a combination of synthetic material and biomolecule. Conjugation is usually limited to one component (for example, the gold on gold‐decorated nanoparticles), requiring more consideration for size and concentration (**Figure** **1**).

To emphasize the ability of bioconjugation to enhance functionality, this review discusses the conjugation of novel materials spanning biological, hybrid, and synthetic categories. While materials such as “smart” polymers are often covered, there are few extensive reviews on the conjugation of novel materials more broadly. By comparing a wide breadth of papers, we aim to highlight which conjugation methods are well suited to certain material types, define unique challenges for each material, highlight projects that might inspire future work, and provide a comprehensive guide that is particularly suited to researchers working in interdisciplinary fields. Due to the sheer mass of bioconjugate research, this scope is limited to medical applications with a special emphasis on therapy, diagnostics and related topics.

**Figure 2:**
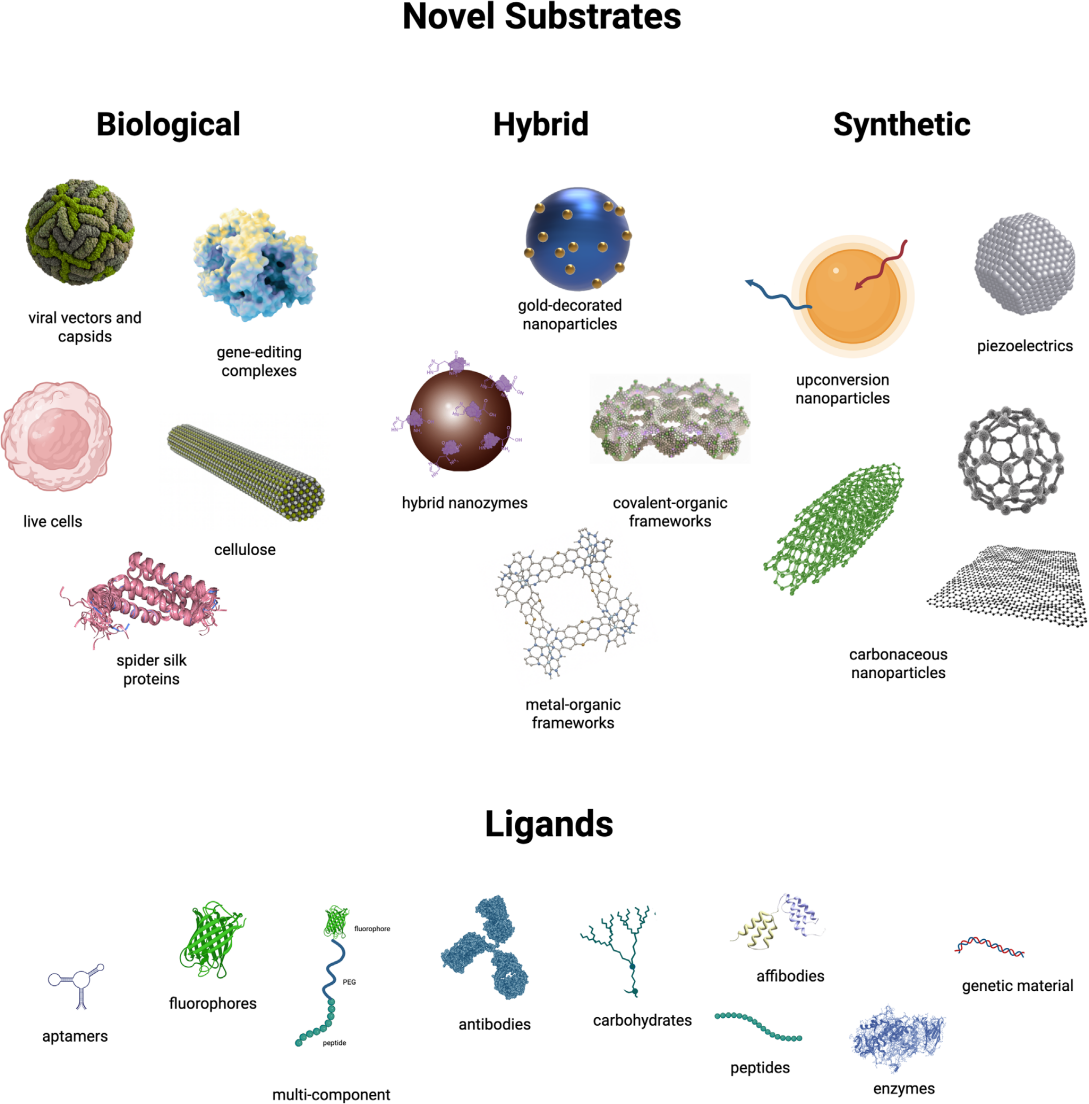
Examples of novel biological, hybrid and synthetic substrates (and their ligands) that we will highlight in this review. Created in Martin, C. (2025) https://BioRender.com/i7lixcs.

We begin by exploring these applications and the materials used to achieve them. These include viral capsids, live cells and other biological substrates; gold‐decorated nanoparticles, covalent‐organic frameworks and other hybrid substrates; and piezoelectrics, upconversion nanoparticles and other synthetic substrates (please place Figure 2 here!). Material properties often breed a preference for certain conjugation methods, and these are underlined in each case. A broader discussion is informed by comparing methods across materials and can be found at the end of the section as a general guide. We next describe characterization techniques to help readers determine whether their own conjugates are successfully bound, stable, and oriented correctly, among other traits. We conclude by reflecting on the current regulatory landscape for bioconjugates and future directions for the field. A general overview of conjugation techniques can be found in the Supporting Information; this provides context for the approaches used in each study and is particularly useful when it comes to enzyme‐, protein‐ and aptamer‐mediated conjugation, which are less common. It also explains our focus on covalent and high‐affinity techniques (**Figure** **2**).

## 2 Novel substrates and applications

### 2.1 Biological substrates

Several unique biological substrates have been used for cutting‐edge medical research upon conjugation. These include viral vectors and capsids; gene editing complexes; live cells; spider silk proteins; and cellulose. Given the highly specific nature of biomolecules (in which key residues are vital for biological function), care should be taken when choosing the conjugation strategy. Thiol‐ and amine‐based strategies risk depositing ligands on top of key cysteines and lysines, and the use of organic solvents risks denaturation. As a result, certain biological substrates tend towards the same few conjugation methods, some of which are quite unique themselves (these methods, which include enzyme‐, protein‐ and aptamer‐mediated conjugation, are described under Supporting Information). We point out these trends and highlight unique challenges accordingly.

#### 2.1.1 Viral Vectors and Capsids

As some of the most abundant entities on earth,^[^ ^6^ ^]^ viruses come in a staggering array of morphologies that can be classified as helical, polyhedral, complex or enveloped. Despite their impressive capabilities, most capsids are made up of repeating units of just one or a few proteins. This signature makeup aids conjugation and generates more predictable properties, simplifying therapy and medical approval processes.^[^ ^7^ ^]^ Capsids, viral vectors and virus‐like particles (VLPs) can be used to load drugs and other cargo, and their protein composition enhances biocompatibility and biodegradation. Plant viruses are also non‐pathogenic to human and animal models.^[^ ^8^ ^]^ Researchers have harnessed these qualities to produce unique conjugates for gene therapy, drug delivery, vaccines, and other applications.

**Figure 3:**
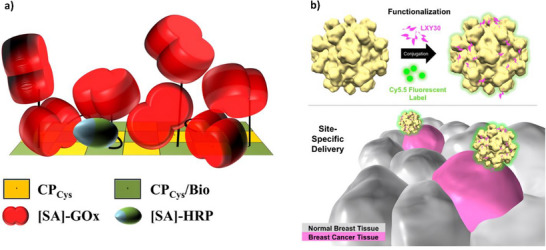
a) Schematic of streptavidin‐GOx and streptavidin‐HRP attached to TMV surface coat proteins (CPs). A 22‐fold excess of biotin was used to conjugate every second cysteine‐modified coat protein (CP_Cys_/Bio). Reproduced under the terms of the CC BY license.^[^ ^8^ ^]^ Copyright 2015, the Authors. b) Schematic demonstrating the targeting capabilities of LXY30‐ and Cy5.5‐conjugated Hepatitis E VLPs. Reproduced with permission.^[^ ^21^ ^]^ Copyright 2016, JoVE.

Positional control is important to bio‐bio conjugation, particularly when it comes to enzyme scaffolding. The precise and convenient organization of cascading enzymes is a hallmark of many living systems, and researchers are increasingly interested in scaffolding/co‐localizing enzymes for applications in diagnostics, therapy and cascade modelling. The highly ordered nature of capsids lends itself to this goal as enzymes can be conjugated with precise, nanometer‐scale spacing. For example, Koch et al. used Michael addition and biotin‐streptavidin to immobilize glucose oxidase (GOx) and horseradish peroxidase (HRP) onto tobacco mosaic virus (TMV) particles. The TMV was genetically modified to exchange serine 3 on each coat protein for a cysteine, ensuring uniform surface thiols for Michael addition of a maleimide‐PEG‐biotin linker before attaching commercial streptavidin‐enzymes. This enabled a two‐step cascade wherein byproduct hydrogen peroxide from GOx could be used as a substrate for HRP to generate a colorimetric signal (**Figure** **3**a). The resulting assay provided glucose detection with impressive sensitivity, reusability and shelf‐life compared to standard assays. Indeed, conjugates provided 45‐fold higher catalytic activity than microtiter plates immobilizing the same concentrations of GOx and HRP. Performance was credited to the dense loadings, steric accessibility and enzyme stabilization provided by the TMV surface.^[^ ^8^ ^]^ Enzymes and cofactors have been immobilized within self‐assembling capsids through methods such as SpyCatcher/SpyTag,^[^ ^9^, ^10^ ^]^ Michael addition^[^ ^11^ ^]^ and electrostatic interactions,^[^ ^12^, ^13^ ^]^ creating protocells for enzyme prodrug therapy, metabolic supplementation, and immunomodulation.^[^ ^14^, ^15^, ^16^ ^]^

Given the ease with which viruses enter cells and insert their genetic material, viral gene therapy is a burgeoning field of research. However, its high transfection efficiency is often nuanced by safety concerns. For example, adenovirus (ADV) is attractive because it can carry large segments of DNA, however ADV enters most host cells through the coxsackie‐adenovirus receptor (CAR). Many cell types express CAR, meaning target‐specific gene therapy requires repeated local injections that can provoke serious innate and adaptive immune responses. To get around this problem, Oh et al. used EDC/NHS coupling to conjugate surface amines on GFP‐encoded ADV vectors with folate‐polyethylene glycol (FOL‐PEG) ligands. PEG has been shown to reduce immune responses by forming a hydrophilic “cloud” around nanoparticles that shields interactions with immune cells and reduces protein adsorption.^[^ ^17^ ^]^ Folate was included to selectively target KB tumor cells, which overexpress folate receptors due to the vitamin's role in rapid cell turnover.^[^ ^18^ ^]^ GFP expression in KB cells increased by ca. 45% when FOL‐PEG conjugates were used compared to naked vectors, however this was not observed when a folate receptor deficient cell line was used (A549). Immune responses measured by interleukin 6 release from macrophages decreased by ca. 40% and 60% in the case of PEG and FOL‐PEG vectors, respectively, highlighting an ability to improve both precision and safety in gene therapy.^[^ ^19^ ^]^ Leray et al. restated the need for both qualities, arguing that genetic modification to this end reduces vector yield and cross‐species translatability. The group used diazo‐coupling to conjugate adeno‐associated virus (AAV) tyrosines with N‐acetylgalactosamine (GalNAc) and mannose for targeting the liver and retina, respectively. GalNAc is known to target asialoglycoprotein receptors on hepatocytes, while mannose targets specific mannose receptors on retinal pigment epithelium. Conjugation in each case improved transduction efficiency both in vitro and in vivo: for example, immunolabels indicated 3‐fold accumulation in target cells when conjugates were used over naked capsids. Minimal accumulation of conjugates was detected in off‐target tissues.^[^ ^20^ ^]^

Chen et al. underscored capsids’ ability to tolerate extreme pH environments and intestinal proteases, making them exciting candidates for orally‐delivered chemotherapeutics. The group constructed a VLP from hepatitis E and modified it to replace a native asparagine (N573) with cysteine so breast cancer recognition peptide LXY30 could be attached via Michael addition (Figure 3b). Tracking with the additional attachment of near‐infrared (NIR) Cy5.5 fluorophores demonstrated high specificity and accumulation both in vitro (more than fivefold) and in tumor‐bearing mice. The cysteine modification also caused the VLP to appear immune silent by disrupting interactions with a known neutralizing antibody. The authors highlighted the platform's ability to encapsulate drugs, DNA/siRNA and inorganic particles for targeted delivery.^[^ ^21^ ^]^

While viral particles are susceptible to recognition and rapid clearance, this is sometimes a boon. Thrombolytics represent the only standard of care for treating abnormal blood clots such as ischemic stroke; however, these drugs lack specificity and can break down healthy blood clots, causing life‐threatening blood loss. This is particularly common for streptokinase and tissue plasminogen activator (tPA), which convert circulating plasminogen to plasmin so it can digest fibrin and break up the clots. Drug‐eluting stents or catheters can be used to direct thrombolytics to the site of disease, but these require resource‐intensive clinical settings and have limited access when it comes to the brain. To remedy this issue, Pitek et al. used NHS coupling and Michael addition to conjugate tPA to solvent‐exposed lysines on TMV, whose rod‐shaped nature increases margination to vessel walls. Conjugates demonstrated a similar therapeutic efficacy to free tPA with a decreased risk of hemorrhage. Both candidates achieved 100% artery re‐opening within 120 minutes in a mouse model, however TMV‐tPA showed a negligible increase in bleeding time relative to a PBS control, while free tPA doubled bleeding time. The conjugate's improved safety profile was attributed to the rapid immune clearance of TMV, which was largely removed from circulation within 10 minutes of injection. In this way, tPA's circulation time was shortened just enough to curtail off‐targets without compromising its therapeutic window.^[^ ^22^ ^]^

Out of all the unique properties conferred by viral substrates, most applications make use of their predictable protein chemistry by using equally precise conjugation methods. Techniques such as EDC/NHS and Michael addition either make use of well‐placed lysine and cysteine residues or are paired with genetic modification. As seen above, native residues are often mutated to cysteine so Michael addition can react with its thiol group at a low risk of misplacement (cysteine is one of the least abundant amino acids).^[^ ^23^ ^]^ Selected residues tend to be located away from key active sites required for viral entry and are sometimes located on immunogenic sites so their modification confers immune silence. This careful placement also ensures in‐vivo capsid assembly is preserved where needed. Certain icosahedral plant viruses are characterized by more surface‐accessible tyrosines, promoting the use of diazo coupling. We also observe more cases of SpyCatcher/SpyTag and enzyme‐mediated conjugation, as these are inherently precise due to the selective nature of biomolecules.

#### 2.1.2 Gene Editing Complexes

CRISPR‐Cas9, zinc‐finger nucleases (ZFNs) and TALENs are the most commonly‐used gene editing tools to date. Despite their differences, all three have the potential to be modified for enhanced performance, imaging, delivery and epigenetic editing. TALEN TALE domains provide long and repetitive binding sites that can stabilize multiple ligands, while ZFNs present lower‐risk substrates for nonspecific conjugation, containing just four key residues necessary for function (two cysteines and two histidines).^[^ ^24^ ^]^

**Figure 4:**
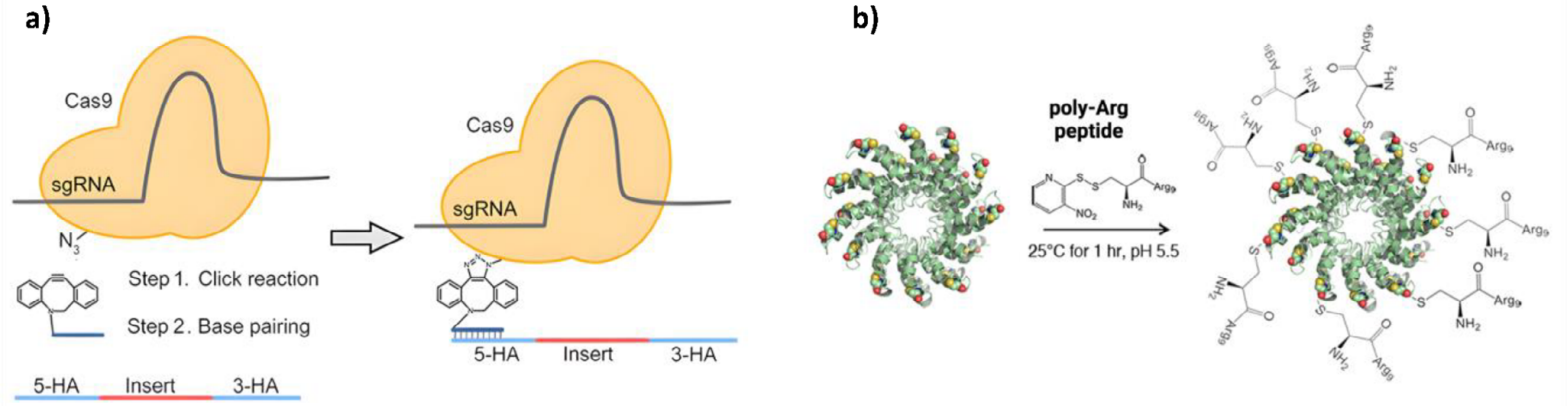
a) Schematic representation of SPAAC adaptor conjugation and the recruitment of donor DNA through base pairing for improved CRISPR‐Cas9 HDR efficiency. Reproduced under the terms of the CC BY‐NC license.^[^ ^25^ ^]^ Copyright 2020, the Authors. b) Disulfide conjugation of Npys Arg_9_ peptides for enhanced cell penetration of TALENs.Adapted under the terms of the CC BY 4.0 license.^[^ ^30^ ^]^ Copyright 2014, the Authors.

Despite rapid advances in CRISPR‐Cas9, precise editing remains a challenge due to the low efficiency of homology‐directed repair (HDR), which supports point substitutions and gene insertions. One factor limiting HDR efficiency is the availability of donor DNA at the site of cleavage. Donor DNA tends to be delivered separately from the Cas9 complex and thus may not localize to the break in time or at high enough concentrations. To address this, Ling et al. used genetic code expansion (GCE) technology to equip Cas9 with a noncanonical amino acid, permitting strain‐promoted azide‐alkyne cycloaddition (SPAAC) for the conjugation of “adaptor” oligonucleotides to the protein. The resulting conjugate recruited donor DNA through base pairing to the adaptor ligands, effectively carrying the DNA directly to the site of cleavage (**Figure** **4**a). As a result, HDR efficiency improved in both human cell lines (HEK293) and mouse zygotes (1.6‐ and 2.3‐fold, respectively, compared to standard unconjugated Cas9).^[^ ^25^ ^]^

CRISPR is also limited by low uptake of the ribonucleoprotein (RNP) complex due to its large size, structural complexity, and immunogenicity. Uptake can be enhanced by encoding nuclear localization sequences (NLS) into constructs, producing RNPs with NLS peptides fused to Cas9's N‐ or C‐terminus for recognition by nuclear transport receptors. The presence of multiple sequences can disrupt Cas9 folding and activity, so most protocols include only one or two NLS tags. By attaching four additional NLS tags (two peptides containing two motifs) to Cas9 using enzyme‐mediated conjugation, Lobba et al. increased editing efficiency 20‐fold compared to unmodified Cas9 as demonstrated by fluorescent reporter expression in neural progenitor cells. Conjugation was performed using tyrosinase, which oxidized exposed tyrosines on the peptides to react with thiol groups on Cas9's two native cysteines, conveniently located away from the active site.^[^ ^26^ ^]^

TALENs usually require carriers—yet TALEN pairs are too large to fit in workhorse AAV vectors, and the repetitive nature of effector domains leads to rearrangements during packaging.^[^ ^27^ ^]^ Carriers’ long circulation times also increase off‐target nuclease activity.^[^ ^28^ ^]^ As a workaround, researchers are using conjugation to impart free‐diffusing TALENs with cell‐penetrating properties. Liu et al. employed poly‐Arg cell‐penetrating peptides (CPPs),^[^ ^30^ ^]^ which dock to cell membranes and induce lamellar stacking for passive entry through fusion pores.^[^ ^29^ ^]^ The CPPs were functionalized with a nitropyridyl (Npys) group, permitting disulfide conjugation to exposed cysteines on the TALEN protein (Figure 4b). Disulfide exchange forms a bond that is cleavable under reducing conditions, meaning CPPs could be released from the TALENs after cytosolic entry. CPP‐TALEN conjugates achieved CCR5 gene knockout frequencies of ca. 16% in HeLa cells, nearly 3‐fold higher than vector controls, with no apparent toxicity. Interestingly, only 8:1–15:1 peptide:TALEN ratios showed activity.^[^ ^30^ ^]^

While a few additional papers describe the covalent attachment of polymers and synthetic drugs,^[^ ^31^, ^32^ ^]^ bioconjugation of gene‐editing complexes is noticeably scarcer than that of other biological substrates, and its primary application is improving the efficiency of gene editing through enhanced cellular uptake of either complexes or genetic material. This is likely because current approaches already achieve suitable efficiencies for many applications (which tend to involve basic knockouts), and they are often simpler and more scalable. As a result of this scarcity, there is no clear common approach to the conjugation of these materials. It is unsurprising that bio‐mediated and azide‐alkyne chemistries have been used, however, given the need to preserve function. Depending on the trajectory of current and breakthrough technologies, conjugation may become vital to high‐fidelity editing.

#### 2.1.3 Live Cells

Researchers are increasingly interested in decorating cell surfaces for therapeutic delivery, cell imaging, and the study of intrinsic and pathological interactions. Cells make for unique substrates as they contain a wide variety of extracellular proteins, complexes and receptors that vary by cell type, expanding the range (and therefore function) of targeting ligands. While antibodies and dyes have traditionally been used to functionalize cells, they lack the more permanent nature provided by high‐affinity and covalent bioconjugation, limiting their use in long‐term studies. Genetic modification can also be applied to stimulate extracellular expression of ligands, however this is characterized by unpredictable and inconsistent expression levels and faces many challenges in vivo.^[^ ^33^ ^]^ Bioconjugation can provide a higher degree of control if the right techniques are used.

In a study by Cui et al., T‐cell transmembrane receptor protein tyrosine kinase 7 (PTK7) was labelled with DNA strands via aptamer‐mediated conjugation. Each aptamer was hybridized with a semi‐complementary DNA strand functionalized with NHS and FITC at either end. Upon aptamer binding to PTK7, the fluorescent DNA strand was brought close enough to the protein to bind nearby lysine residues via its NHS ester. Flow cytometry was used to verify enhanced fluorescence in CEM T‐cell variants, which express high levels of PTK7, relative to Ramos T‐cell controls, which express low levels. The authors described the study's use towards in vivo cell studies and future therapeutic functionalization.^[^ ^34^ ^]^

**Figure 5:**
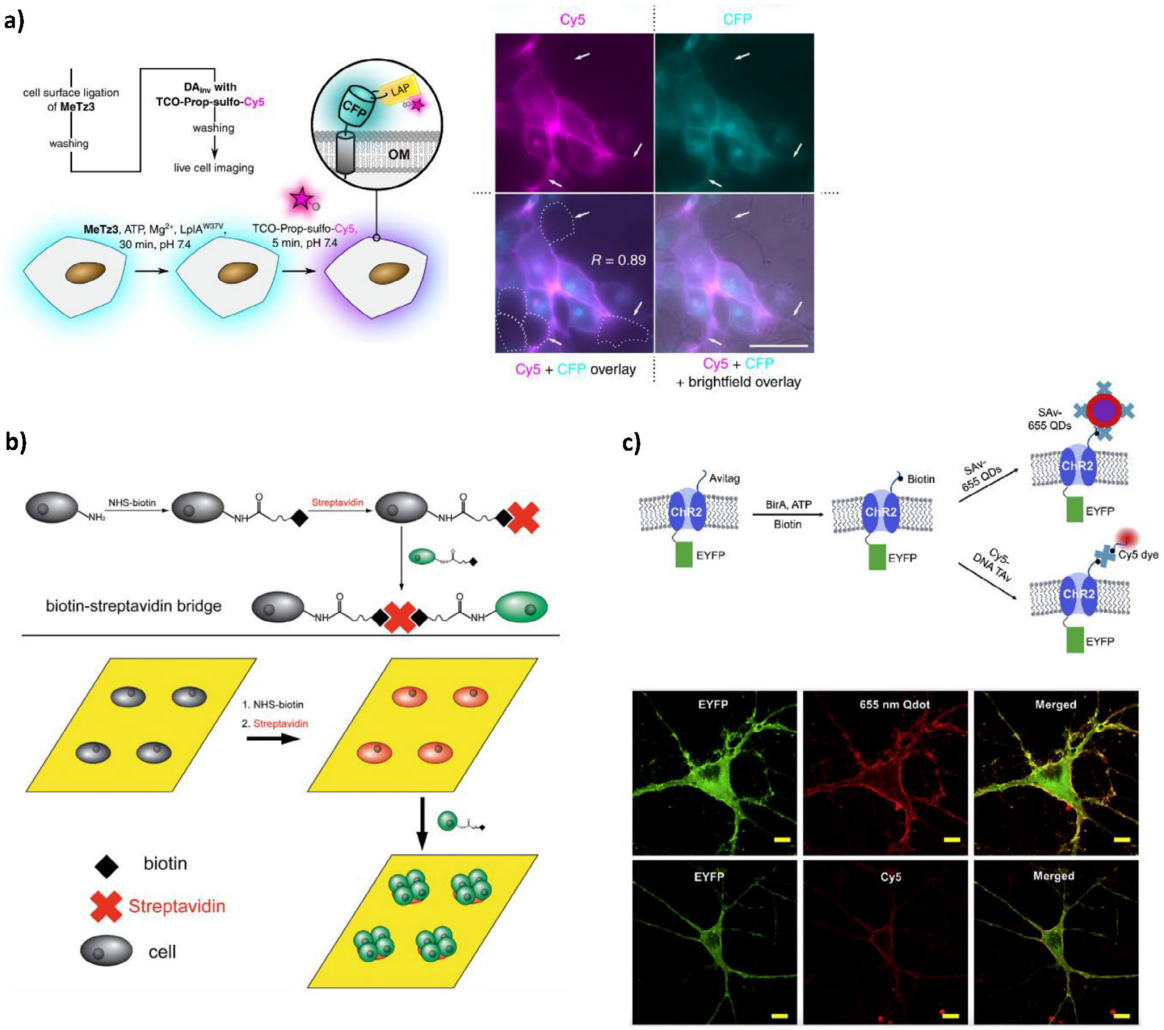
a) **Left**: schematic demonstrating the extracellular labeling process for HEK293 cells transfected with pDisplay‐LAP‐CFP‐TM. The engineered cells are first ligated with reactive dienes (e.g., MeTz3, or 3‐hydroxymethyl‐6‐methyl tetrazine) via enzyme‐mediated conjugation, which is supplemented with magnesium acetate, ATP, and LplA. DAinv is then performed using TCO‐Prop‐sulfoCy5, contributing a pink fluorescence. **Right**: fluorescent images of live‐cell‐labelled membrane proteins. The top left image shows Cy5 fluorescence from conjugated cells, while the top right image displays the natural CFP fluorescence of the same cells. Merged images (bottom left and right) indicate a high specificity of conjugation, as demonstrated by the overlap of Cy5 and CFP signals. White arrows in each image indicate nontransfected cells serving as internal negative controls. Scale bar = 25 µm. Adapted with permission.^[^ ^35^ ^]^ Copyright 2019, American Chemical Society. **b)** Schematic diagram of cell surface conjugation and the stepwise formation of multicellular structures. The yellow sheet represents a fibronectin‐coated SIRM made from polydimethylsiloxane (PDMS), grey cells represent SMCs, and green cells represent HUVECs. Reproduced with permission.^[^ ^38^ ^]^ Copyright 2013, WILEY‐VCH Verlag GmbH & Co. KGaA, Weinheim. **c) Top**: schematic illustration of live cell biotinylation followed by secondary conjugation with streptavidin‐quantum dots (QDs) and traptavidin‐DNA‐Cy5, respectively. **Bottom**: fluorescent images of unconjugated neurons are shown on the left hand side (ChR2 was expressed in a construct with enhanced yellow fluorescent protein, or EYFP). The middle images show red fluorescence produced by quantum dot conjugation on the top and Cy5 conjugation on the bottom. Merged images are shown to the right, demonstrating opsin‐specific conjugation. Reproduced with permission.^[^ ^39^ ^]^ Copyright 2020, Elsevier Inc.

Following this line of thinking, Baalmann et al. used enzyme‐mediated conjugation for applications in fluorescence microscopy. Lipoic acid protein ligase A (LpIA) was used to attach reactive dienes onto cell‐surface cyan fluorescent protein (CFP) encoded with LpIA acceptor peptide (LAP). Cy5 fluorophores tagged with trans‐cyclooctene (TCO) were incorporated via Diels‐Alder inverse electron‐demand click chemistry (DAinv), forming a covalent bond between the peptide and TCO and producing a clear, photostable image with precise localization (**Figure** **5**a).^[^ ^35^ ^]^

In a more extensive study, Zhang and Raymo functionalized the fluorescent molecule rhodamine with NHS, enabling it to bind to primary amines on extracellular proteins. The researchers went a step further by functionalizing a membrane‐permeable fluorophore with NHS so the compound could non‐selectively label intracellular proteins upon entry, resulting in lysosome imaging. HaloTag and SNAP‐tag conjugation were then used to label specific intracellular proteins of interest. Notably, all fluorophores were photoactivatable, meaning they could switch from non‐emissive to emissive upon specific illumination (“super‐resolution microscopy”). This approach circumvented the fluorescence quenching and background fluorescence that occurs when fluorophores are exposed to continuous illumination, enabling visualization of specific cellular functions down to the nanometer scale.^[^ ^36^ ^]^ Hayashi et al. studied target proteins using an antibody‐enzyme complex to tag epidermal growth factor receptor (EGFR) and human epidermal growth factor receptor 2 (HER2) with ester‐functionalized fluorophores. The antibody was used to locate the target receptor, while the enzyme (4‐dimethylaminopyridine, or DMAP) was used to selectively conjugate receptor residues such as lysines. As a result, the group was able to monitor the cellular dynamics and lifetime of HER2 endogenous to cancer cells, suggesting the receptor exhibits a more dynamic structure than crystallography suggests. When combined with a peptide mass fingerprinting analysis, the EGFR conjugate facilitated epitope mapping of antibodies on living cells, identifying new potential binding sites.^[^ ^37^ ^]^

Cell conjugation is largely applied towards imaging, but it is in no way limited to such applications. For example, Gong et al. used ligand interactions to form a bilayer of smooth muscle cells (SMCs) and human umbilical vein endothelial cells (HUVECs). The bilayer was rolled into 3D tubes, mimicking in vivo tubular structures such as blood vessels, where different cell types interact and communicate. The bilayer was formed by adhering SMCs to a stress‐induced rolling membrane (SIRM) and conjugating the cells with sulfonated NHS‐biotin via covalent interactions with cell surface amines, followed by a streptavidin coating. The HUVECs were similarly conjugated with NHS‐biotin and added to the SMC layer to form strong biotin‐streptavidin interactions (Figure 5b), after which the SIRM was released to form the 3D shape. This work has applications in tissue engineering, where tubular structures are difficult to mimic.^[^ ^38^ ^]^ Membrane biotinylation has also been applied in optogenetics: Bang et al. used the enzyme BirA to conjugate biotin to genetically‐encoded AviTag peptide expressed on transmembrane opsin channelrhodopsin‐2 (ChR2). Optogenetics is limited by the fact that many opsin proteins are inefficient at converting light into ion flow, meaning high‐intensity light is needed for effective neuromodulation. This problem is exacerbated by light scattering and absorption by biological tissues, which can be damaged by the generated heat. Metallic and upconversion nanoparticles are increasingly used for treatment with safer low‐intensity light, but in many cases the particles freely diffuse and interact with off‐target cells in the brain. Bang et al.’s approach enabled neuron‐specific tagging by streptavidin‐coated quantum dots (in addition to traptavidin‐DNA‐Cy5 ligands), demonstrating proof of concept targeting through fluorescent imaging (Figure 5c). Biotinylated quantum dots were also directly conjugated onto the transmembrane ChR2. Future studies might therefore replace the quantum dots or Cy5 with metallic or upconversion nanoparticles for the targeted conversion of low‐intensity light, enhancing optogenetic efficiency through a safer means.^[^ ^39^ ^]^

Brasino et al. demonstrated the potential for high therapeutic efficiency at patient‐tolerable levels by conjugating anti‐EGFR affibodies to cancer cells. Deep tissue penetration is limited in antibody‐based immunoconjugates due to their low dissociation constants (K_D_​) and large Stokes radii, causing tumor recurrence. To remedy this problem, cysteine‐mutated affibodies were conjugated with maleimide‐benzophenone ligands via Michael addition, as benzophenone is capable of photocrosslinking. Upon near‐UV irradiation, interactions between cell EGFR and affibody conjugates were converted to covalent bonds, showing enhanced retention for up to 24 hours relative to controls. In a separate test, upconverting nanoparticles were diffused alongside the conjugates to convert clinically relevant, deep‐penetrating NIR radiation into UV for crosslinking, with similar retention levels. The work's therapeutic relevance was expanded in a follow‐up study in which prodrug enzyme cytosine deaminase (CodA) and fluorescent markers were conjugated to the affibody. Affibody structures were shown to undergo receptor‐mediated endocytosis within 12 hours, followed by recycling back to the cell membrane within 24 hours. Inhibited ERK signaling and prolonged prodrug therapy suggested promising clinical potential. High cell viability was maintained in monolayer and 3D spheroid models under both near‐UV and NIR irradiation, as demonstrated by MTT assays.^[^ ^40^ ^]^

Since most proteins contain primary amines, ester‐based approaches (particularly NHS) are commonly used for the nonspecific surface tagging of live cells. While primary amines are largely associated with lysine side chains, surface‐exposed N‐termini may also be accessible, particularly in the case of single‐pass proteins. Because N‐termini influence protein folding and function, ester‐based conjugation should proceed with caution when these are exposed. Precise tagging of target proteins often employs enzyme‐, protein‐ or aptamer‐mediated conjugation. Apart from offering site‐selectivity, these “mediated” techniques forgo potentially harsh chemical conditions, decreasing the odds of denaturation or reduced cell viability. Many examples of mediated conjugation are covered in the above examples, demonstrating a pattern similar to viral substrates.

#### 2.1.4 Spider Silk Proteins

Spider silk's unique properties have sparked increasing interest over the past few decades. Spider silk structural proteins (“spidroins”) are composed of crystalline beta‐sheet regions interspersed with amorphous domains, providing incredible strength and elasticity, even in water.^[^ ^41^ ^]^ Their amphiphilic nature promotes self‐assembly into fibers, films, and other structures that are biocompatible and offer tunable degradation rates, and some of these are transparent or adhesive.^[^ ^42^, ^43^, ^44^ ^]^ These properties naturally lend themselves to bioconjugate applications in tissue engineering and wound dressing. Natural silk is difficult to scale up due to low yields that are exacerbated by spider cannibalism,^[^ ^45^ ^]^ so recombinant spidroins are typically used.

**Figure 6:**
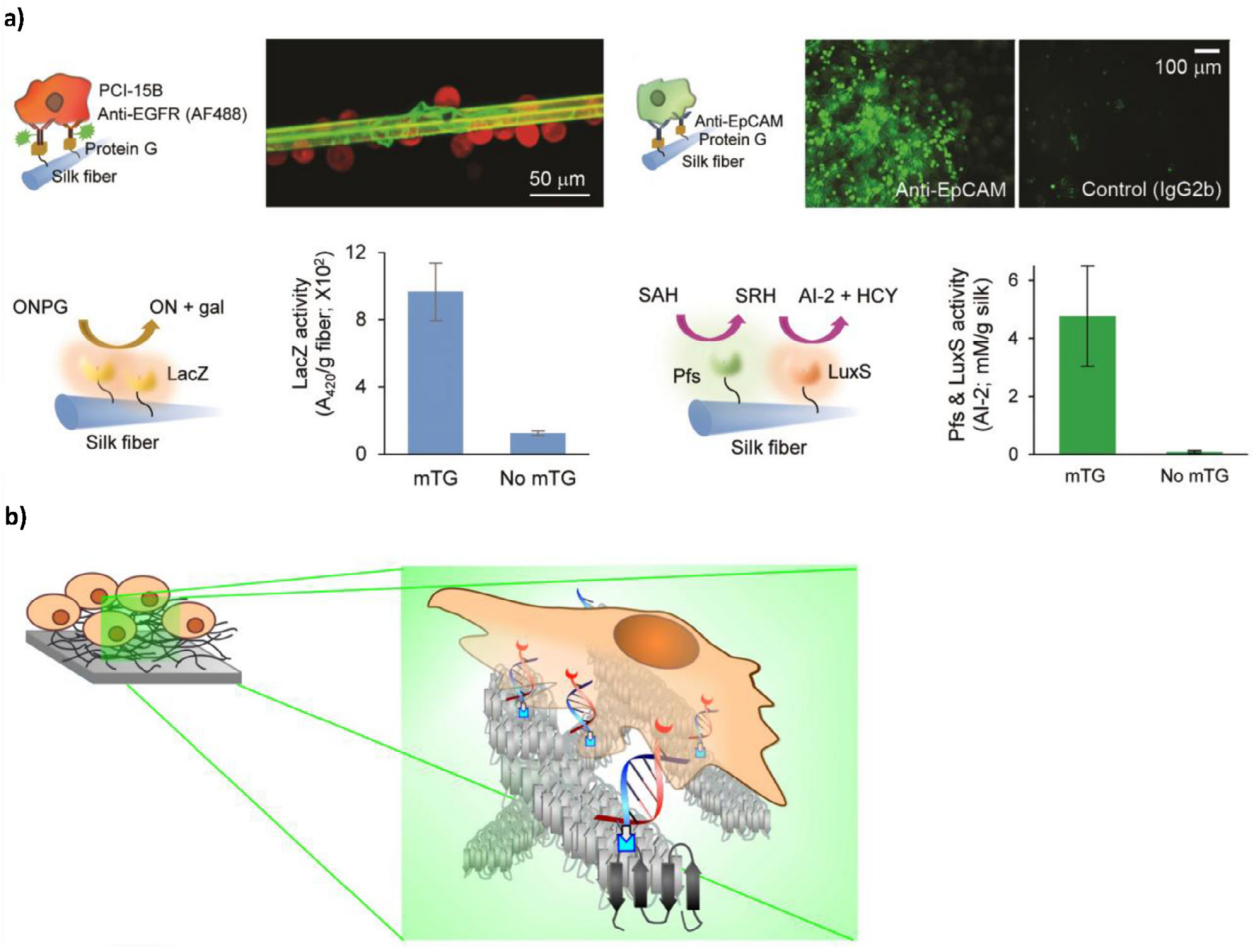
a) Top: cell capture by antibody ligands. The left‐hand schematic illustrates PCI‐15B cell capture on spider silk conjugated with protein G and anti‐EGFR antibody labelled with green fluorescent Alexa Fluor 488. A confocal image to the right shows PCI‐15B cells loaded with red fluorescent Calcein as they are captured onto the spider silk complex. The right‐hand schematic illustrates HT29 cell capture on spider silk conjugated with protein G and anti‐EpCAM (epithelial cell adhesion molecule) antibody. Cells were loaded with green fluorescent Calcein acetoxymethyl (Calcein AM) to allow visualization (confocal image to the right of the schematic). Control spider silk conjugated with mouse IgG2b antibody showed minimal cell capture (far right), demonstrating the cell‐specific nature of conjugation. Bottom: activity of enzyme ligands. The right‐hand graph shows enzymatic activity of LacZ conjugated to spider silk as measured by 420 nm absorption (corresponding to ONP, the product of ONPG hydrolysis by LacZ), relative to unconjugated spider silk (treated with LacZ but no mTG). The left‐hand graph shows enzymatic activity of Pfs and LuxS as measured by the presence of homocysteine (HCY), the product of LuxS activity, relative to unconjugated spider silk (treated with Pfs and LuxS but no mTG). Adapted with permission.^[^ ^46^ ^]^ Copyright 2016, Wiley Periodicals, Inc. b) Schematic illustrating cell capture by the self‐assembled network of spider silk proteins. The enlarged image to the right shows the spider silk conjugates and their captured cells in more detail. Light blue structures attached to the grey spider silk proteins represent azido groups, while the light grey arrows “clicking” into the azido groups represent DBCO. DNA, shown in blue, is attached to the DBCO groups. Attached to the Jurkat cells and shown in red is lipid‐DNA complementary to the spider silk conjugates. Adapted with permission.^[^ ^47^ ^]^ Copyright 2022, American Chemical Society.

Wu et al. used enzyme‐mediated conjugation to decorate spider silk fibers with a number of ligands including antibodies, enzymes, protein tags and fluorescent proteins (**Figure** **6**a). Conjugation was performed by tagging each ligand with pentaglutamine and employing microbial transglutaminase (mTG) to catalyze bond formation between pentaglutamine acyls and spider silk lysines. Bound antibodies selectively captured head and neck carcinoma cells, showing potential for diagnostics and tissue therapy. Bound LuxS and Pfs enzymes recruited *E. coli* onto the fibers through simulated quorum sensing, showing potential for localized infection control and wound healing dressings. Through careful spatial organization of antibodies and enzymes, fibers were shown to perform both tasks simultaneously.^[^ ^46^ ^]^

Harvey et al. functionalized recombinant spider silk (4RepCT) with fluorophores and the antibiotic levofloxacin via copper‐catalyzed azide‐alkyne cycloaddition (CuAAC). To prepare for the click reaction, 4RepCT proteins were grown in methionine auxotroph *E. coli* to replace methionine amino acids with L‐azidohomoalanine (AHA), which contains an azide group. Ligands were pre‐functionalized with alkyne groups. The paper demonstrated how the same approach could be used to adapt silks for different applications in wound dressing, infection control and tissue regeneration.^[^ ^45^ ^]^ In a paper published by Heinritz et al., nonadherent Jurkat cells were immobilized onto a network of azido‐modified spider silk proteins conjugated with short nucleic acid sequences via SPAAC click chemistry (Figure 6b). Immobilization was made possible by incorporating complementary DNA into the membrane of the cells, with applications in tissue engineering.^[^ ^47^ ^]^

As highlighted above and in a recent review,^[^ ^48^ ^]^ azide‐alkyne click chemistry is often used to conjugate spider silk. It is biorthogonal and can proceed under mild conditions, preserving the integrity of enzyme and antibody ligands that are frequently attached. Methionine replacement to AHA is also straightforward compared to other biological systems. This is partly because spider silk, as noted earlier, is mostly used in its recombinant form: it is expressed in the same systems used to incorporate AHA azide groups (e.g., *E. coli* hosts). Spider silk also presents more of a “platform” substrate than an “active” one, meaning changes to methionine are unlikely to jeopardize function. In contrast, live cells could suffer as methionine is necessary for translation.

#### 2.1.5 Cellulose

Like spider silk, cellulose fibers are known for their high mechanical strength owing to crystalline structures. Cellulose is capable of forming hydrogels, which are useful for maintaining moisture and controlling the release of antimicrobial agents and other compounds (which can be further enhanced by enlarging internal pores).^[^ ^49^, ^50^ ^]^ Cellulosic scaffolds have emerged with mounting pressure to use sustainable biomaterials over synthetic polymers and animal‐derived materials for tissue engineering.^[^ ^51^ ^]^ They are decorated with surface hydroxyl groups that are increasingly exposed on nanostructures, some of which offer a significant surface‐to‐volume ratio. Cellulose nanostructures come in a variety of forms such as rod‐like cellulose nanocrystals (CNCs); flexible cellulose nanofibrils (CNFs); bacterial nanocellulo, such as rod‐like cellulose nanocrystals (CNCs), flexible cellulose nanofibrils (CNFs), bacterial nanocellulose (BNC), surface‐functionalized hairy bacterial nanocellulose (HCN).^[^ ^52^, ^53^, ^54^ ^]^

**Figure 7:**
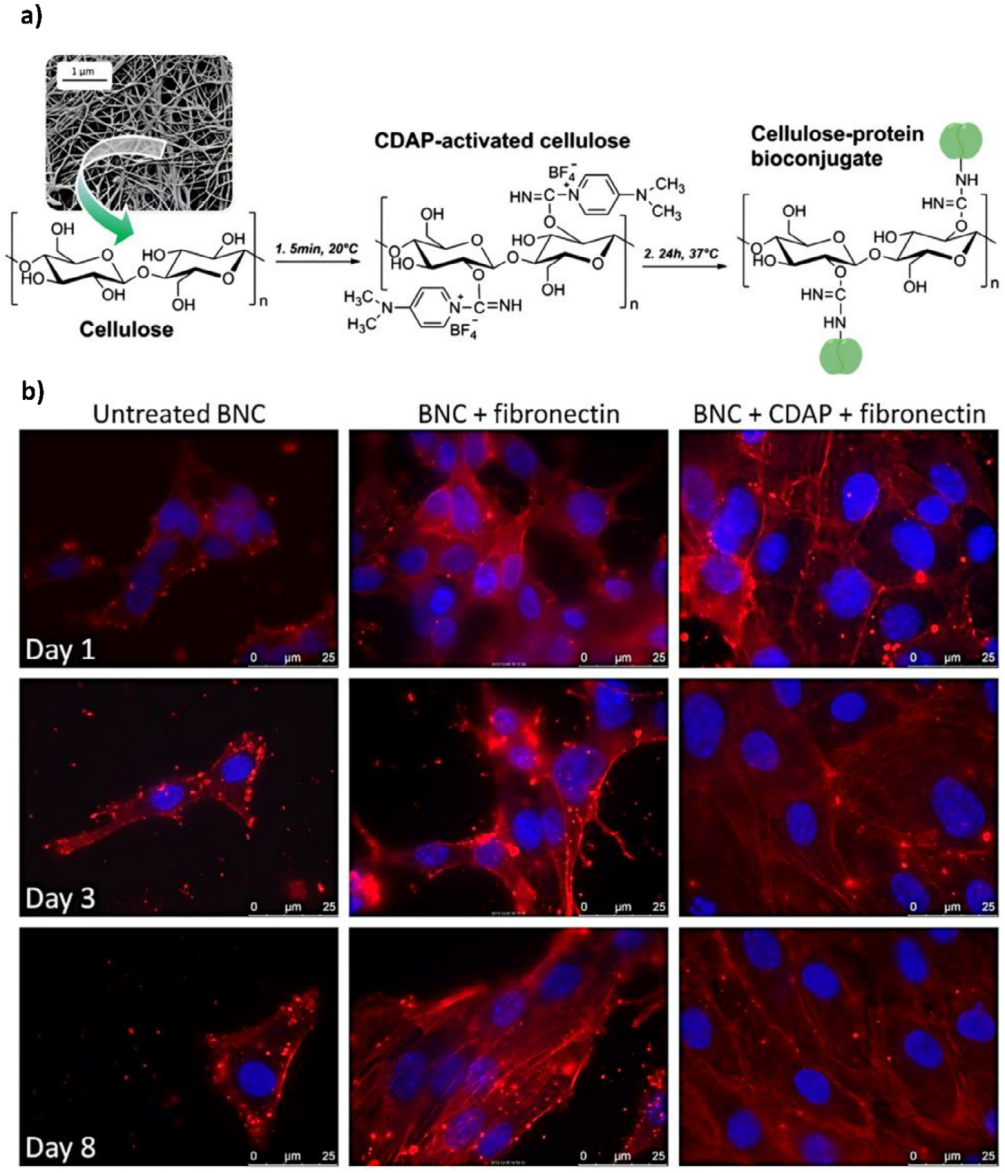
a) Cellulose bioconjugation using CDAP (SEM image of BNC pictured on the top left). b) Fluorescence microscopy images of HUVECs on unmodified BNC, fibronectin‐adsorbed BNC, and fibronectin‐conjugated BNC after 1, 3 and 8 days of culturing. Cell nuclei stained with DAPI can be seen in blue and actin filaments stained with rhodamine‐phalloidin can be seen in red. As shown, the covalently conjugated surface facilitated the growth of many adhered cells with well‐defined actin filaments, acquiring a cobblestone morphology typical of endothelial cells. Adapted with permission.^[^ ^55^ ^]^ Copyright 2013, Elsevier B.V.

In light of these features, Kuzmenko et al. used coupling agent 1‐cyano‐4‐dimethylaminopyridinium (CDAP) to activate hydroxyl groups on the surface of BNC matrices, forming esters capable of reacting with extracellular matrix proteins fibronectin and collagen type I. This addition significantly enhanced the scaffold's ability to promote HUVEC and mouse MSC adhesion, and cell viability also improved. Conjugation occurred rapidly in mild, aqueous conditions, enhancing BNC's potential for tissue regeneration and repair (**Figure** **7**).^[^ ^55^ ^]^

HNC is a relatively novel class of cellulose. These particles are characterized by needle‐like crystalline regions capped with amorphous, hair‐like chains that are easily functionalized.^[^ ^53^ ^]^ Ojagh et al. took advantage of this feature by introducing carboxyl groups onto the “hair” regions through periodate and chlorite oxidation. Carbodiimide‐like activating reagent DMTMM (4‐(4,6‐Dimethoxy‐1,3,5‐triazin‐2‐yl)‐4‐methylmorpholinium chloride) was then used to facilitate conjugation between the carboxyl groups and primary amines on PLL (ɛ‐poly‐L‐lysine), an antibacterial naturally secreted by *Streptomyces* and related bacteria. The conjugates were found to be effective against *E. coli* and *Bacillus subtilis*, with applications in wound dressing.^[^ ^56^ ^]^ In a similar conjugation approach, Raja et al. converted CNC hydroxyls to carboxyls using TEMPO‐mediated oxidation, followed by EDC/NHS coupling for attachment to amine‐functionalized PEG‐biotin. The same process was used for amine‐functionalized perylene diimide (PDI) dye. The conjugates were used to label and image a range of cell lines including J774A.1 macrophages, NIH‐3T3 fibroblasts, HeLa adenocarcinoma cells, and primary murine dendritic cells. PDI‐CNCs demonstrated superior photostability compared to commercially available dyes, and the particles were successfully internalized and localized to perinuclear regions of the cytoplasm, demonstrating potential applications in long‐term cell tracking.^[^ ^57^ ^]^

Goodge and Frey used cellulose nanofibers to produce a paper‐like binding surface for sensitive and rapid point‐of‐care diagnostics. The nanofibers were decorated with alkynes by substituting backbone hydroxyls with propargyl ethers through the use of propargyl bromide, and these were then conjugated to azide‐PEG‐biotin using CuAAC click chemistry. Streptavidin‐FITC was added to demonstrate the even and concentrated distribution of hydroxyls, which are key to enhancing diagnostic performance (with other potential applications such as filtration).^[^ ^58^ ^]^

When it comes to cellulose substrates, conjugation techniques such as oxidation combined with EDC/NHS or more direct activation reactions tend to be common due to the even distribution of hydroxyl groups on CNC surfaces, and the fact that nonspecific binding is more acceptable for many cellulose use‐cases such as cell grafting and wound dressing. In cases where higher specificity, stability or biocompatibility is desired, biorthogonal click chemistry may be preferred.

#### 2.1.6 Characteristics and Challenges of Biological Substrate Conjugation

As highlighted, bio‐bio conjugation embodies a wide range of approaches, and these are often more complicated than those used for synthetic substrates (as we will detail below). However, the diverse yet specific nature of biomolecule surfaces means ligation can become more precise. For example, it is impossible to consistently place ligands four nanometers apart on a gold nanoparticle. This can, however, be realized on a viral capsid. Bio substrates often contain key functional moieties such as active or unfolding sites, and as a result biorthogonal click chemistry and bio‐mediated conjugation are more likely to be used, as these proceed in biological environments and allow the precise ligation just mentioned. However, the frequent need for priming—often through genetic expression of azides, peptides or protein tags—adds a degree of complexity. Simpler methods such as EDC/NHS, Michael addition and diazo coupling, which conjugate native surfaces with at most one modification step (e.g., oxidation), are thus a good fit for substrates that lack active moieties (for example cellulose, spider silk and certain live cells and capsids depending on the application).

Following this logic, the biggest challenge facing biological substrates is conjugation in vivo, which while uncommon is not unheard of.^[^ ^34^ ^]^ EDC/NHS risks off‐target interactions with abundant amines, and while Michael addition provides some degree of specificity given that cysteine residues are less common, this requires local delivery and implies that target molecules must contain well‐placed cysteines. Priming for bioorthogonal or mediated techniques in this dynamic environment requires either genetic modification (typically using vectors); natural expression of relevant markers (such as lipid II in bacterial cell walls, which sortase A recognizes and ligates);^[^ ^59^ ^]^ or metabolic incorporation (e.g., feeding bacteria with azide‐sugar analogs to produce azide‐functionalized biomolecules).^[^ ^60^ ^]^ Given this enhanced degree of complexity, it is safe to assume that most forms of bioconjugation will continue to be performed in vitro.

### 2.2 Hybrid substrates

As material design has evolved over time, surface morphology has become increasingly complex. Substrates such as gold‐decorated particles, nanozymes, covalent organic frameworks (COFs) and metal‐organic frameworks (MOFs) present heterogeneous surfaces that require careful consideration when conjugating. On the other hand, COFs and MOFs are incredibly predictable, and all of these substrates are less inherently specific than biological substrates. As a result, they are characterized by a smaller pool of conjugation methods. As described in the introduction, hybrid substrates can be synthesized using biological and synthetic materials or a mix of synthetic materials. Their inherent multi‐functionality can assist bioconjugation and/or enhance the intended applications, which range from antibacterial coatings to enantiomer separation.

#### 2.2.1 Gold‐Decorated Nanoparticles

Gold nanoparticles have been extensively covered in bioconjugate literature thanks to unique properties such as tunable surface plasmon resonance and the fact that gold readily forms strong dative bonds with thiol groups. An emerging class of materials functionalizes larger base particles with smaller (ca. 2–20 nm)^[^ ^61^, ^62^ ^]^ gold particles that are embedded across the surface, combining the unique properties of both materials to form “gold‐decorated nanoparticles”. This also facilitates bioconjugation for materials that are difficult to functionalize otherwise. Although these substrates are relatively novel, several cases of bioconjugation have already been reported.

**Figure 8:**
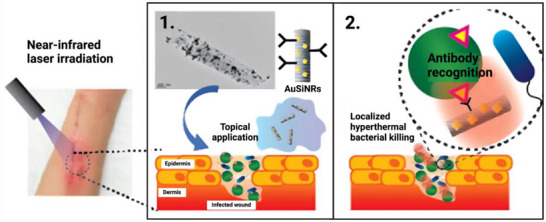
Schematic depicting the use of gold‐decorated nanopillar/antibody conjugates for topical antibiotic treatment of infected diabetic wounds. A micrograph (top center) shows a single nanopillar decorated with gold nanoparticles ranging from 2–50 nm. Adapted with permission.^[^ ^64^ ^]^ Copyright 2017, American Chemical Society.

Ozhikandathil et al. used gold‐decorated multi‐walled carbon nanotubes to construct a novel biosensor. The particles demonstrated highly‐sensitive (pg/mL) label‐free detection of recombinant bovine growth hormone (rbST) as a proof of concept, which was monitored through UV‐vis absorption courtesy of the localized surface plasmon resonance (LSPR) effect. Linkers containing thiols on one end and carboxyls on the other were used to attach anti‐rBST antibodies via EDC/NHS coupling. Performance was linked to dense rbST loadings afforded by the nanotube, with contact between the nanotube and gold decorations enhancing the local electromagnetic field and amplifying the LSPR signal.^[^ ^63^ ^]^ Alhmoud et al. used gold‐decorated porous silicon nanopillars to target pathogenic bacteria in diabetic wounds, highlighting the growing need for topical treatments alongside antibiotics due to overuse of the latter. The nanopillars were first primed with undecylenic acid for the introduction of carboxyl groups onto the silicon component. Similar to Ozhikandathil et al., these were then modified with EDC/NHS for the attachment of anti‐Staphylococcus aureus antibodies (**Figure** **8**). Naked substrates reduced bacterial viability up to 99% after 10 minutes of laser irradiation due to excellent photothermal conversion by the gold decorations. Performance was further enhanced up to 10‐fold after bioconjugation, and the authors highlighted the potential for antibiotic drug incorporation through the silicon nanopillars’ pores.^[^ ^64^ ^]^

Adams et al. immobilized peptides onto gold‐decorated magnetite in a proof‐of‐concept demonstrating surface‐enhanced Raman spectroscopy (SERS) sensing and bioseparation, with implications in diabetes treatment and monitoring. Glucagon‐like peptide 1 (GLP‐1), a gastrointestinal hormone that stimulates insulin release from pancreatic β‐cells, was presented as the ligand. Mild reducing agent tris(2‐carboxyethyl)phosphine (TCEP) was used to expose thiol residues on the peptide, which subsequently bound the gold decorations via dative interactions. SERS enabled GLP‐1 detection at concentrations as low as 10^−7^ M with no need for reporter molecules, and bioseparation via magnetic interactions was conducted in approximately 30 seconds.^[^ ^65^ ^]^ Several non‐covalent approaches have also been used for biosensor/immunoassay applications.^[^ ^66^, ^67^, ^68^ ^]^

Most gold‐decorated nanoparticles can be expected to bind ligands via the gold decorations rather than the base surface. This is due to a combination of projected surface, ease of conjugation, and the vast array of gold conjugate literature. As a result, techniques commonly applied towards larger plain gold nanoparticles also apply here. Due to its simplicity and strong covalent nature, dative bonding between gold surfaces and naturally‐occurring thiol groups is commonly employed. When thiol groups are unavailable or can't be coaxed out of the ligand, EDC/NHS may be used after functionalizing the gold with carboxyl‐thiol linkers such as mercapto acids or amine‐thiol linkers such as cysteamine. Both dative bonding and EDC/NHS were used in the papers described above. Electrostatic approaches are also popular, and indeed the remainder of gold hybrid literature takes this approach.^[^ ^69^ ^]^ However, it bears reminding that due to the heterogenous nature of these surfaces, conjugation needs to be optimized to overcome the limited surface area of gold. This challenge, which characterizes hybrid materials at large, is discussed at the end of this section.

#### 2.2.2 Hybrid Nanozymes

Nanozymes are an emerging class of particles that catalyze reactions by mimicking the characteristic properties of enzymes such as oxidase, peroxidase, catalase and superoxide dismutase.^[^ ^70^ ^]^ They can be synthesized from a range of materials including metals, metal oxides and carbon‐based materials with the benefit of improved stability, cost and tunability compared to many biological enzymes. The synthesis of nanozymes varies by precursors used, and approaches include coprecipitation, thermal decomposition, seed‐mediated growth, and hydrothermal/solvothermal synthesis from bulk materials and precursors through top‐down and bottom‐up methods. Many nanozymes contain just one uniform surface material, however synthesis methods are sometimes adjusted to produce hybrid nanozymes. For example, biomolecules such as histidine can be introduced into the reaction medium during hydrothermal or solvothermal synthesis, creating histidine‐modified nanoparticles with enhanced catalytic activity.^[^ ^71^ ^]^ Other nanozymes may be decorated, or contain Janus domains or dopants. We refer to this subset as “hybrid nanozymes”. Conjugating ligands widely vary, as do their applications.

**Figure 9:**
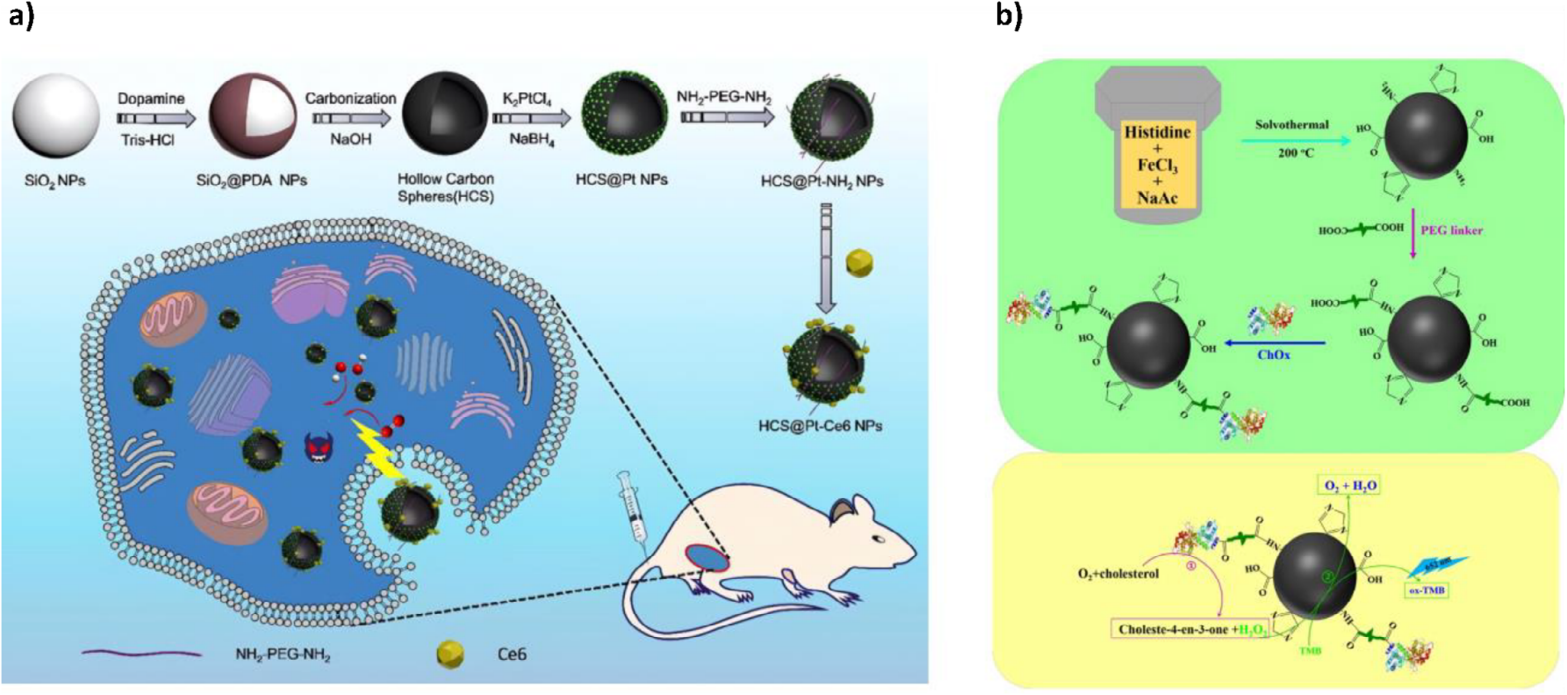
a) Schematic depicting the synthesis, decoration and conjugation of hollow carbon sphere nanozymes, with applications in tumor therapy demonstrated using mice models. Reproduced with permission.^[^ ^72^ ^]^ Copyright 2020, Elsevier B.V. b) Top: full synthesis process for the nanozyme‐ChOx conjugate. Bottom: schematic representation of the conjugate‐mediated cascade resulting in TMB oxidation for the quantification of cholesterol. Reproduced under the terms of the CC BY license.^[^ ^73^ ^]^ Copyright 2023, the Authors.

Xu et al. used platinum‐decorated hollow carbon spheres for synergistic tumor therapy (**Figure** **9**a). The platforms were ligated with chlorin e6 (Ce6), a photosensitizer derived from chlorophyll. Conjugation applied EDC/NHS in two separate steps, first joining amine‐PEG‐amine linkers to carboxyls present on the carbon HCS surface before joining Ce6 to the linkers. Platinum decoration conferred peroxidase‐ and oxidase‐like activities, catalyzing the generation of reactive oxygen species (ROS) from endogenous hydrogen peroxide and oxygen in tumor microenvironments. Ce6 ligands contributed their own ROS upon red light stimulation, and as a result, 4T1 tumor cell apoptosis increased from ca. 15% with carbon spheres alone to ca. 31% with the conjugates.^[^ ^72^ ^]^

Nanozymes are often ligated with natural enzymes. One example is 3β‐hydroxysteroid oxygen oxidoreductase (also known as cholesterol oxidase, or ChOx), a protein that catalyzes the oxidation and isomerization of cholesterol and generates H_2_O_2_ as a byproduct. Cholesterol is a versatile molecule that mediates immune function, brain synapse formation, and cancer progression. It also presents a biomarker for cardiovascular diseases, nephrosis, anemia and liver diseases. With this in mind, Zhao et al. used a solvothermal method to synthesize histidine‐modified iron oxide nanoparticles, as histidine has been shown to improve the peroxidase‐like activity of iron oxide through interactions between its imidazole group and H_2_O_2_. ChOx was conjugated to the nanozymes using a two‐step EDC/NHS approach similar to Xu et al.’s through a dual‐ended‐carboxyl PEG linker, which bound amine groups on the histidine surface components. As cholesterol was oxidized by ChOx, byproduct H_2_O_2_ could be oxidized by the hybrid substrate alongside 3,3′,5,5′‐tetramethylbenzidine (TMB), producing a colorimetric signal for cholesterol present (Figure 9b). The conjugates were easily removed through magnetic separation so as not to interfere with quantitative detection. Assays displayed limit of detection (LOD) and limit of quantification (LOQ) values as low as 0.446 and 1.488 µM, respectively.^[^ ^73^ ^]^

Given that nanozymes are a broad material category to begin with (a large number of materials display enzyme‐like activities), there are no definitive patterns in conjugation chemistries. With respect to ligands, PEG linkers are commonly used for a few potential reasons. Firstly, they space biomolecules apart from their substrates, making room for catalytic activity to take place. Secondly, they can be equipped with a range of end groups (such as carboxyls, amines, sulfhydryls and hydroxyls), promoting facile attachment to a variety of surface materials. Thirdly, PEG incorporation increases biocompatibility, and all hybrid nanozymes contain at least one inorganic or synthetic component. While nanozyme hybridization often improves catalytic properties, the added surface components can also facilitate conjugation, as in the case of gold‐decorated nanoparticles and the following materials, COFs and MOFs.

#### 2.2.3 Covalent Organic Frameworks

Covalent organic frameworks (COFs) are a class of porous crystalline nanomaterial typically made from two or more organic monomer building blocks, and they form promising bioconjugate substrates for several reasons. COFs are formed by covalently linking each monomer, meaning individual monomers can be specifically chosen to incorporate amines, thiols, azides, and similar groups capable of directing ligand attachment (functional groups can also be added through the bonds themselves).^[^ ^74^ ^]^ Pore size can be optimized for cargo encapsulation, selective filtering and the creation of microenvironments. This porosity, coupled with high surface areas, also increases the potential concentration of cargo molecules. As a result, COFs have been applied towards medical applications such as enhanced oxygen delivery, drug delivery, ROS generation, and bioimaging.^[^ ^75^, ^76^ ^]^ COF bioconjugation is an emerging field of research that has largely centered around enzyme immobilization,^[^ ^77^ ^]^ perhaps due to the efficient spatial organization conferred by COF frames (reminiscent of enzyme co‐localization in viral capsids).

**Figure 10:**
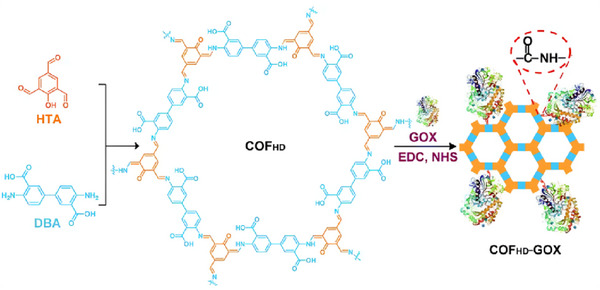
Schematic depicting the synthesis process for the two‐component COF (COF_HD_) and the COF_HD_‐GOx conjugate. As can be seen, GOx is attached to the DBA components (shown in blue). Reproduced with permission.^[^ ^78^ ^]^ Copyright 2021, Partner Organizations.

Yue et al. used EDC/NHS to conjugate a two‐component COF with glucose oxidase for the colorimetric detection and quantification of glucose in urine, serum and beverages. The COF was synthesized from 2‐hydroxybenzene‐1,3,5‐tricarbaldehyde (HTA) and 4,40‐diamino‐3,30‐biphenyldicarboxylic acid (DBA), monomers that were selected for their stability and the presence of carboxyl groups (in the case of DBA) (**Figure** **10**). The COF_HD_‐GOx conjugate displayed a wide linear range (0.005 mM‐2 mM) and an LOD of 0.54 µM under optimal testing conditions. Conjugates were tested against multiple saccharides and were shown to be highly selective towards glucose. More than 85% stability was retained after storage in PBS for over 100 days, indicating practical use within healthcare settings.^[^ ^78^ ^]^

Racemic compounds (or “racemates”) are mixtures of chiral molecule enantiomers. The ability to separate enantiomers from racemates is key to developing and characterizing chiral drugs, since enantiomers sometimes fatally differ in their pharmacodynamics and toxicity profiles. Enantiomeric purity is usually characterized by HPLC, which separates racemates through chiral stationary phases (CSPs) made from materials such as silica. Traditional CSPs often lack fine control over pore size, which can limit their ability to separate complex or bulky chiral molecules. Fortunately, pore control is a hallmark of COFs. Zhang et al. developed lysozyme‐COF conjugates for the successful separation of 11 different racemates. The COF was synthesized through a condensation reaction to form a polyimide‐based framework. Carboxyl groups formed from the condensation reaction (and therefore located near the junctions between monomers) were used for EDC/NHS conjugation of the lysozymes. Given that lysozymes are naturally chiral, these interacted with one enantiomer more strongly than the other to achieve separation. The COFs protected the lysozymes from leaching and denaturation, and the platform retained high efficiency after more than 120 runs over two months, demonstrating their practical use in devices such as HPLC.^[^ ^79^ ^]^

Given the presence of functional moieties, covalent techniques such as EDC/NHS, Michael addition and CuAAC click chemistry are often employed towards COF conjugation, although physisorptive methods have also been used in many cases (the latter presents virtually no risk of enzyme denaturation, and COF conjugates tend not to be applied towards highly dynamic environments).

#### 2.2.4 Metal Organic Frameworks

Metal‐organic frameworks (MOFs) are another type of porous crystalline material related to COFs, but with certain key differences. MOFs are composed of metal node building blocks joined together by organic “linker” molecules. These entities are held together by “coordinate bonds” in which the organic molecule donates electron pairs to the metal ions or clusters.^[^ ^80^ ^]^ Each MOF typically employs just one type of metal and one type of linker molecule, but the contrast between the two forms a unique hybrid surface for conjugation. Like COFs, MOFs benefit from highly reproducible structures, high surface areas and tunable pores that lend themselves to a variety of biomedical applications, particularly biosensing. Due to the presence of functional groups such as amines, carboxyls and hydroxyls on the organic linkers, biomolecule ligands are almost exclusively conjugated to these components. MOFs can be synthesized as individual particles, which occupy the nanometer scale, or as crystals, which fall within the micrometer to millimeter scale.^[^ ^81^ ^]^ Most bioconjugation experiments are performed on individual MOF nanoparticles, so we will focus on these applications.

Bhardwaj et al. used EDC/NHS to conjugate photoluminescent MOF nanoparticles with anti‐*Staphylococcus aureus (S. aureus)* antibodies for biosensing. The MOF employed benzene derivative 2‐aminobenzene‐1,4‐dicarboxylic acid (NH_2_‐BDC) as its organic linker, which provided the photoluminescence via its aromatic ring. Photoluminescent energy levels were enhanced by the presence of the amine group, which also enabled conjugation. Metal nodes were made from iron in the form of ferric chloride hexahydrate (FeCl_3_·6H_2_O). Conjugation was performed by joining carboxyl groups on the antibody Fc regions to organic linker amines via EDC/NHS. *S. aureas* binding to the conjugates quenched photoluminescence in a manner proportional to bacteria concentration, producing an LOD of just 85 CFU/mL. The biosensor was validated using spiked river water and cream pastry and displayed a high specificity for *S. aureus* in the presence of other bacteria such as *E. coli*. Detection occurred within 20 minutes, and the device produced a stable response when kept for over 60 days. These results demonstrated MOFs’ capacity to immobilize high concentrations of biosensing molecules over small areas, providing portable point‐of‐care diagnostics that could detect bacteria at low concentrations.^[^ ^82^ ^]^

**Figure 11:**
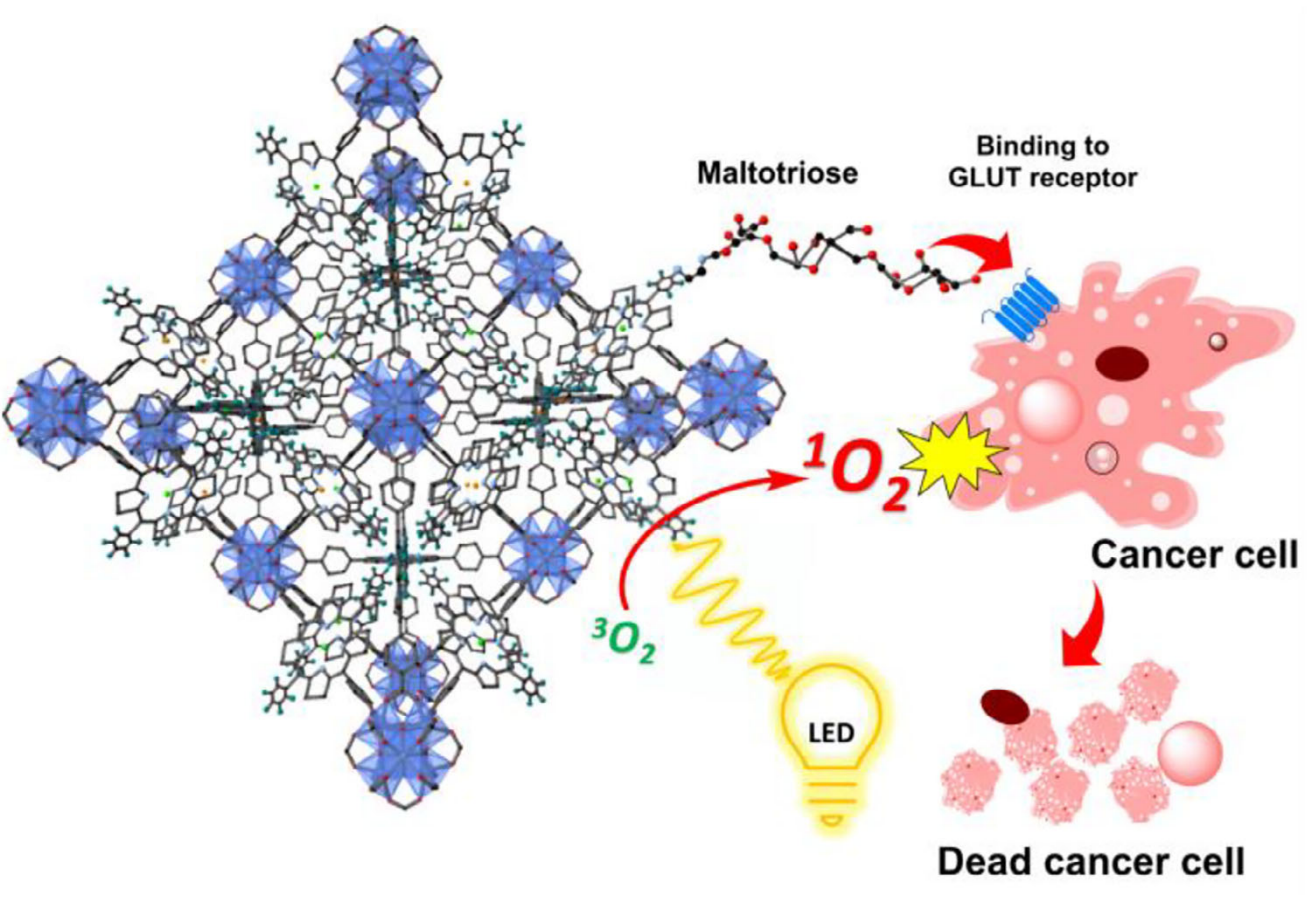
Schematic depicting targeted photodynamic therapy by maltotriose‐MOF conjugates for cancer treatment. Reproduced with permission.^[^ ^84^ ^]^ Copyright 2021, American Chemical Society.

Photodynamic therapy is a promising cancer treatment due to its low systemic toxicity and its ability to selectively destroy light‐accessible tumors,^[^ ^83^ ^]^ and Sakamaki et al. used MOFs as delivery vesicles for this mode of therapy. The MOFs consisted of hafnium (Hf) metal nodes and chlorin organic linkers, with chlorin chosen for its photosensitizer properties. Chlorin absorbs light in the red to near‐infrared (NIR) range and produces high yields of ROS, particularly singlet oxygen, making it well‐suited to deep tissue penetration and tumor eradication. Cancer targeting was conferred through the conjugation of carbohydrate molecule maltriose, which was modified with ethylenediamine (EDA) and attached to the MOF's chlorin groups using nucleophilic aromatic substitution (SNAr), a less conventional conjugation method. The substitution involved a nucleophilic attack by the amino group in EDA‐maltriose onto the aromatic pentafluorophenyl ring on the chlorin molecules, producing a strong covalent bond that enabled selective uptake in breast cancer, pancreatic cancer and cervical cancer cells (**Figure** **11**).^[^ ^84^ ^]^ Alves et al. also applied MOFs towards cancer therapy, however the group used CuAAC click chemistry to attach folic acid ligands for targeting, and the therapeutic payload was delivered by physically encapsulating curcumin into the MOF's pores.^[^ ^85^ ^]^

Given their similarities to COFs, it is unsurprising that MOFs preferentially employ many of the same conjugation techniques, such as EDC/NHS and CuAAC. However, because MOF organic linkers are often more structurally complex than COF monomers, unique conjugation methods such as SNAr can be accommodated. Specific components of interest include heteroaromatic backbones and electron‐withdrawing substituents.^[^ ^86^ ^]^

#### 2.2.5 Characteristics and Challenges of Hybrid Substrate Conjugation

Biomolecules can easily be made to bind surfaces such as gold, however this is nuanced by the fact that most decoration techniques produce surface particles that are unpredictable with respect to distribution and size. Considering sizes typically range from 2–20 nm, decorative particles are more difficult to conjugate from a surface area standpoint, particularly when considering they are partially embedded into the larger surface. The presence of multiple materials can also induce nonspecific binding, which could sterically hinder the conjugation of target sites. For example, positively‐charged metal nodes on MOFs could induce electrostatic interactions with ligands that are meant to conjugate the organic components instead. To remedy this issue, ligand quantification can be employed to determine the amount of starting material (reagent/ligand) necessary to overcome the limited odds of conjugation without promoting off‐target physisorption or side reactions. Given the wide range of materials used to form hybrid substrates, there are virtually no common conjugation techniques as a whole. However in each case, hybridization is often used to enhance performance or structure while simultaneously providing a surface for ligand attachment. In some cases (for example, certain hybrid nanozyme conjugates), this leads to three degrees of functionality that, when aligned in their purpose, can produce highly synergistic properties.

### 2.3 Synthetic substrates

A large portion of bioconjugate research focuses on synthetic substrates such as gold, magnetic, silica, and polymer nanoparticles. These are characterized by useful, predictable and well‐researched properties, however the hunt for new applications and characteristics has resulted in a growing list of conjugate materials. The following materials include piezoelectrics, upconversion nanoparticles, and carbonaceous nanoparticles.^[^ ^87^ ^]^ These materials are selected for their unique properties, which are either enhanced or complemented by functional ligands (or enhance the delivery of functional ligands).

#### 2.3.1 Piezoelectrics

Piezoelectrics are fascinating materials that generate electric potentials when mechanically deformed. This property (the “direct piezoelectric effect”) arises from non‐centrosymmetric crystal structures and can be triggered by remote ultrasound, which is routinely used in medicine to penetrate biological tissue safely and efficiently. At the same time, electrical stimulation produces mechanical strain (the “inverse piezoelectric effect”).^[^ ^88^ ^]^ Piezoelectrics possess resonant frequencies, meaning their crystal structure oscillates at maximum amplitude when subjected to a specific frequency. For this reason, piezoelectrics are often used in sensors such as quartz crystal microbalances (QCMs) for immunoassays. The addition of a tiny mass onto a piezoelectric surface alters its mechanical properties and therefore resonant frequency, which can be detected and correlated.^[^ ^89^ ^]^ Many piezoelectrics also exhibit second harmonic generation (SHG) in which two photons are converted to a single photon with twice the frequency (“second harmonic light”) due to electric field interactions with the crystal's dipoles. While not exclusive to piezoelectrics, this effect is often exploited as a stable alternative to fluorescence for the generation of second harmonic radiation imaging probes (SHRIMPS).^[^ ^90^ ^]^ With such unique properties, it is unsurprising that piezoelectrics have been used for applications such as cancer therapy, stem cell differentiation, immunomodulation, self‐powered implants, and antibacterial coatings. Piezoelectric bioconjugation is still a relatively untapped area of research but has nonetheless been reported in several cases.

**Figure 12:**
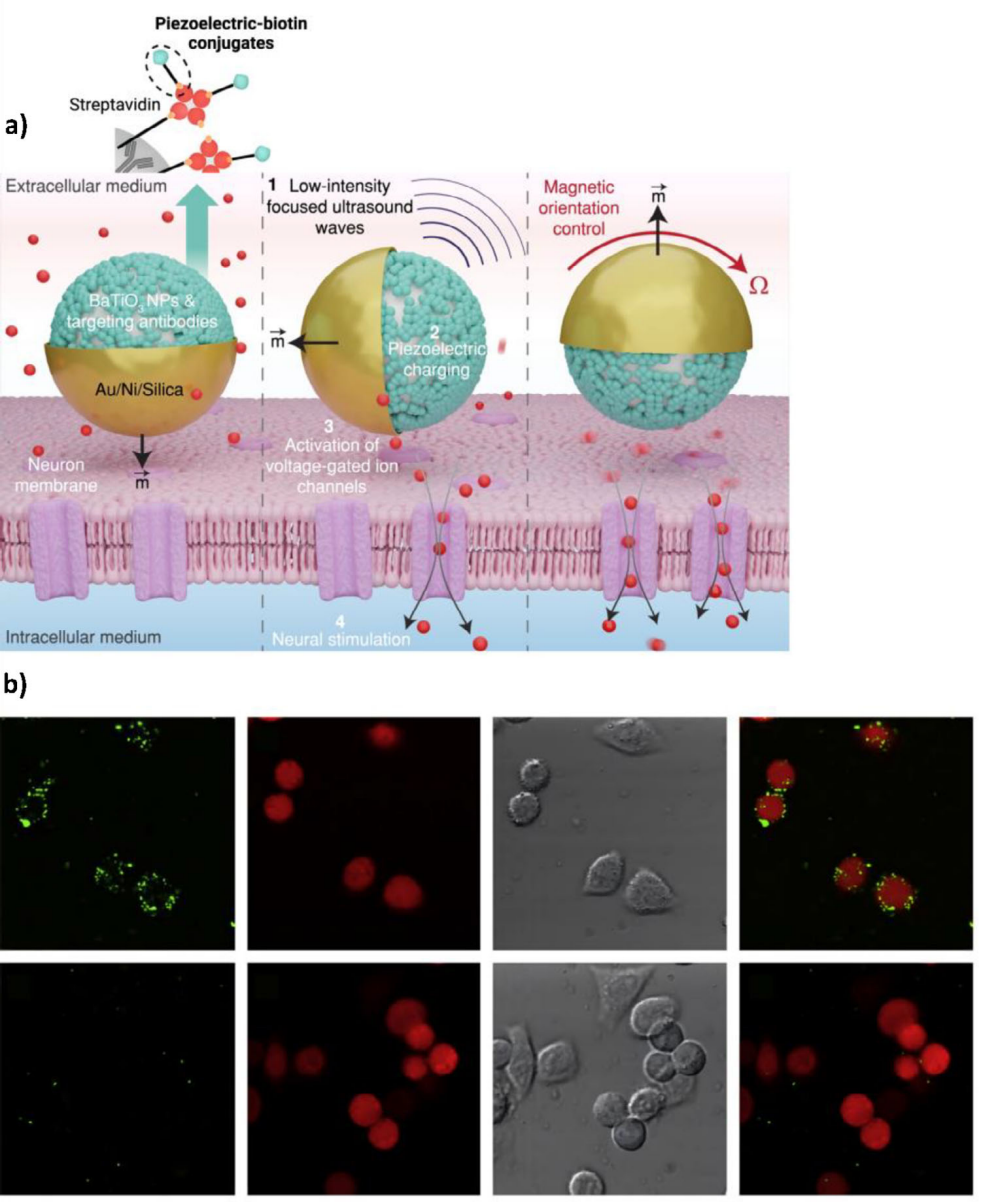
a) Schematic depicting use cases for the larger Janus nanoparticle. The left‐hand side depicts the Janus nanoparticle in extracellular medium, where it can operate in freestanding mode (using magnetically‐actuated locomotion and steering of the Au/Ni half) or cell‐attached mode when flipped (using G‐protein‐coupled inwardly rectifying potassium channel 2, or GIRK2, antibodies interspersed among barium titanate on the other half). The middle image depicts the neuromodulation process: BTNPs generate piezoelectric potentials in the extracellular fluid ^(1)^, which depolarize the cell membrane ^(2)^ to activate voltage‐gated ion channels ^(3)^, inducing neural stimulation ^(4)^. The right‐hand side depicts the orientation that results in maximum neural stimulation, which can be configured through magnetic actuation. Reproduced under the terms of the CC BY 4.0 license.^[^ ^91^ ^]^ Copyright 2024, the Authors. b) Top row: confocal images of HeLa cells labelled with primary antibodies specific to HLA class I molecules, followed by labeling with secondary antibody‐piezoelectric conjugates (SHRIMPs). From left to right: SHG‐marked HLA molecules in green, fluorescent calcein AM‐stained cells in red, transmission images, and merged SHG/fluorescent images. Bottom row: control samples in which cells were not labelled with primary antibody. Reproduced with permission.^[^ ^92^ ^]^ Copyright 2009, Elsevier Ltd.

Barium titanate is a biocompatible material capable of displaying piezoelectricity at the nanoscale. Han et al. used 3‐aminopropyltrimethoxysilane (APTES) to introduce amino groups onto barium titanate nanoparticles (BTNPs) through covalent interactions with surface hydroxyl groups, followed by NHS‐biotin ligation. As a result of bioconjugation, the barium titanate (BTO) nanoparticles could be incorporated onto a larger biotin‐decorated Janus nanoparticle, conferring stimulation of dopaminergic neurons upon ultrasound exposure for the minimally invasive treatment of neurological disorders (**Figure** **12**a).^[^ ^91^ ^]^

Hsieh et al. demonstrated biomarker applications by first treating BTNPs with nitric acid to leach barium ions from the surface, creating a TiO_2_‐like outer shell. APTES was once again used to introduce amine groups before adding sulfo‐SMCC (an NHS‐maleimide linker). This permitted Michael addition with IgG antibodies previously reduced by dithiothreitol (DTT) to yield free sulfhydryl groups at their hinge disulfide regions. The resulting conjugates were used to bind human leukocyte antigen (HLA) class I molecules presented on HeLa cell surfaces in vitro, with femtosecond laser pulses stimulating SHG for clear imaging (Figure 12b). The authors reported less than 5% nonspecific labeling as demonstrated by a primary antibody microarray. SHG signals are generated equally in both the forward and backward direction, enabling efficient quantification through methods such as photoluminescence spectroscopy (provided endogenous SHG is weak, which is the case for most cell surfaces). This approach therefore avoided complications related to fluorescence quenching and enhancement, with additional benefits such as a lack of bleaching over time, a signal unaffected by excitation power, and an extremely fast response time – enabling the observation of rapid and dynamic processes over extended periods.^[^ ^92^ ^]^

While a vast array of literature exists for unconjugated medical applications, piezoelectric surfaces such as BTO are less readily ligated relative to other materials, which is perhaps why their conjugation is less common. Another explanation is that many medical applications are still in the early stages of research, meaning studies are primarily conducted in vitro with no real need for targeting. As research develops and approaches in vivo studies, bioconjugation will likely become more widespread. Indeed, several papers have implicated bioconjugation in future steps.^[^ ^93^, ^94^ ^]^ While reagents such as APTES can incorporate functional amine groups onto the surface of piezoelectrics like BTO, it should be noted that physical hybridization can potentially facilitate piezoelectric conjugation while enhancing performance. For example, gold decoration of BTNPs by Wu et al. was shown to improve ROS generation for ultrasound‐induced bacteria clearance and wound healing.^[^ ^95^ ^]^ Physisorption has also been applied for piezoelectric conjugation,^[^ ^96^, ^97^, ^98^ ^]^ as have polymer ligands.^[^ ^99^ ^]^

#### 2.3.2 Upconversion Nanoparticles

Upconversion nanoparticles, or UCNPs, are exciting materials that have gained significant attention in recent years. UCNPs work by absorbing two or more low‐energy photons and converting them into a single, higher‐energy photon through progressive excitation of dopant ions (typically lanthanides such as Er^3^⁺, Tm^3^⁺, and Ho^3^⁺).^[^ ^100^ ^]^ Lanthanide‐doped NPs specifically convert near‐infrared light into visible and UV emissions.^[^ ^101^ ^]^ Given NIR's frequent use in tissue therapy and other medical applications, these make attractive therapeutic and diagnostic agents. UCNP synthesis methods such as high‐temperature thermal decomposition and low‐temperature aqueous synthesis produce ligated nanoparticles from the outset, which provide stabilization (oleic acid and citrate are the most common “native” ligands).^[^ ^102^ ^]^ These can either be replaced through ligand exchange or modified with functional groups for facile conjugation. Upconversion levels are directly influenced by their immediate environment, providing opportunities for the clever manipulation of light and its interaction with biomolecules. It is therefore unsurprising that UCNP bioconjugation is a rapidly growing field of research, with ligands and particles presenting a unique toolbox of properties.

**Figure 13:**
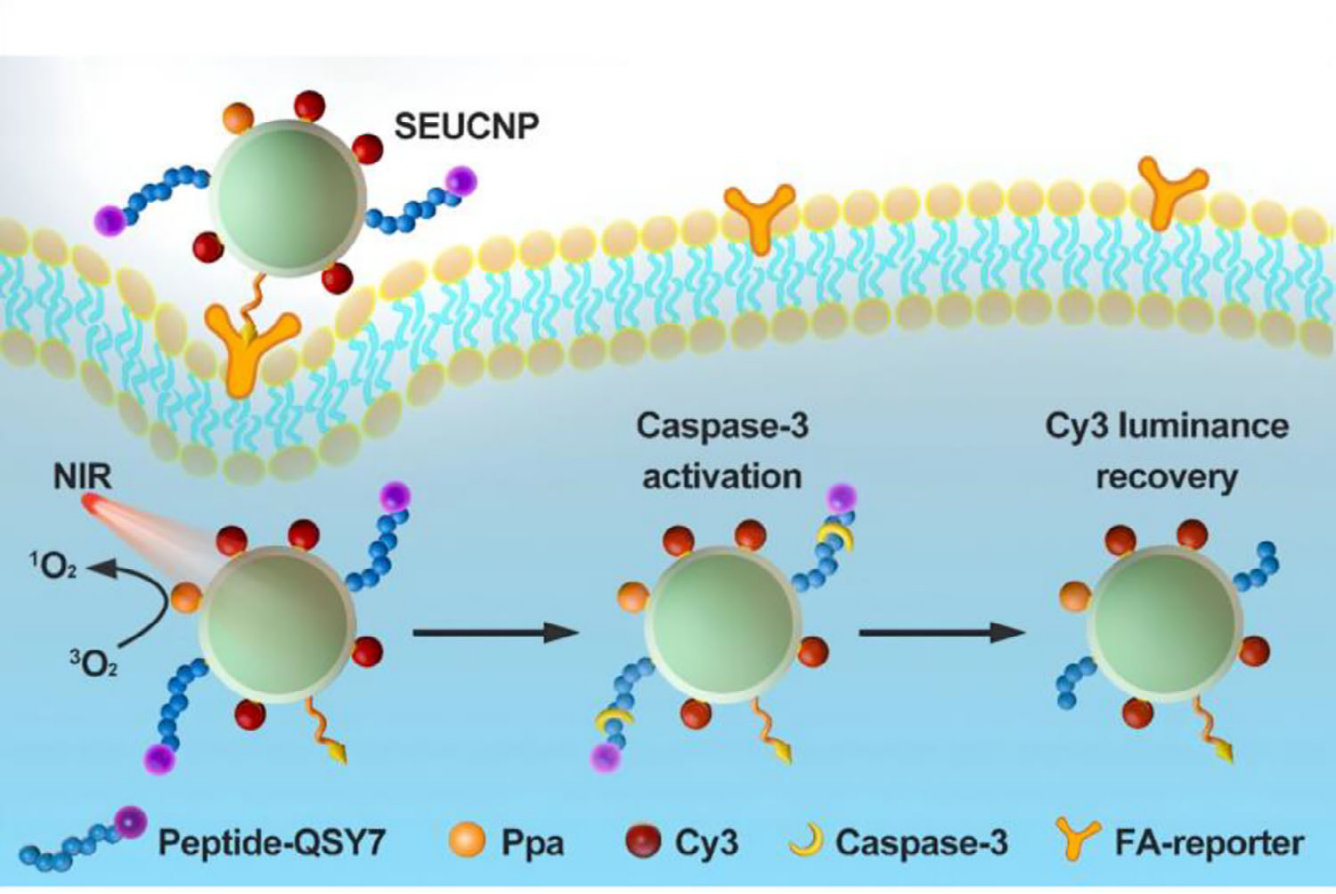
Schematic illustrating the self‐evaluation process for photodynamic therapy via caspase‐3‐induced restoration of Cy3 fluorescence. Reproduced with permission.^[^ ^103^ ^]^ Copyright 2021, Chinese Chemical Society.

For example, Zhang et al. used a set of multiple ligands for targeted photodynamic therapy with self‐evaluation. A photosensitizer ligand pyropheophorbide‐a (Ppa) was shown to produce ROS upon Lanthanide upconversion of NIR light, triggering apoptosis in cervical cancer cells (HeLA cell line). Cy3 fluorophore ligands provided real‐time monitoring of apoptosis, thanks to the addition of a separate two‐component ligand. This consisted of caspase‐3 substrate peptide labelled with quencher molecule QSY7 via Michael addition (caspase‐3 is a biomarker enzyme for apoptosis). Upon caspase‐3‐induced cleavage of the peptide‐quencher ligand, Cy3 fluorescence was restored, monitoring therapeutic effectiveness in real time (**Figure** **13**). Furthermore, PEGylated folic acid was conjugated for targeted cancer cell delivery. Conjugation was achieved by first functionalizing UCNPs with alendronic acid (ADA) in order to introduce amine groups. Each prepared ligand was then activated via NHS‐ester chemistry, forming stable amide bonds.^[^ ^103^ ^]^

Cao et al. explored imaging applications while conjugating UCNPs with antibodies. A ligand exchange was performed to replace oleic acid with colominic acid polysaccharides, creating abundant surface carboxyls. These reacted with antibody lysines through the help of EDC/NHS, and antibodies were found to retain up to 74% binding activity.^[^ ^104^ ^]^ Confocal microscopy, fluorescence spectrometry and flow cytometry were used to demonstrate biosensing and immunofluorescent cell imaging applications with high signal‐to‐noise ratios compared to traditional fluorophore labeling. In the case of Liu et al., dopamine acrylamide was used for ligand exchange prior to conjugation of aptamers and HRP. The presence of “ene” groups post‐exchange enabled a click reaction with the aptamers, which were first thiolated, and cysteine groups on the HRP. As a result, conjugates were shown to bind specifically to CEM and HeLa target cells via the aptamers, and to demonstrate unimpaired enzymatic activity, with applications in drug development and imaging.^[^ ^105^ ^]^

Like fluorophores, UCNP emissions can be quenched or modified if a nearby molecule absorbs some of the energy, in what is referred to as luminescence resonance energy transfer (LRET). When certain cofactor‐containing enzymes are conjugated to UCNPs, emission shifts occur upon changes to the enzyme's redox state and therefore activity. In this context, Burgess et al. applied silica capping to UCNPs, followed by APTES amine functionalization for the incorporation of a sulfo‐SMCC (NHS/maleimide‐containing) cross‐linker. A variety of enzymes were then conjugated via their cysteine residues, including BM3Heme, cytochrome C, glucose oxidase, and glutathione reductase. All ligands were shown to retain enzymatic activity, which could be monitored in real time through LRET.^[^ ^106^ ^]^

Given the variety of approaches used to functionalize UCNPs for bioconjugation, a diverse range of conjugation techniques has been employed over the last several decades. Among these, EDC/NHS and Michael addition are frequently used due to their ability to interact with functional groups that are often added to or substituted with native UCNP ligands (or added to bare particles). Azide‐alkyne cycloaddition is also commonly used and can functionalize modified silica coatings, which are typically added using base alkoxysilanes such as APTES.

#### 2.3.3 Carbonaceous Nanomaterials

Carbonaceous nanomaterials encompass a range of unique substrates including carbon quantum dots, carbon nanotubes, graphene, fullerene, and nanodiamonds. As expanded in the following sections, these materials make promising biomedical agents for a number of reasons. Material precursors can also be derived from biomass, offering biocompatible, eco‐friendly and sustainable synthesis routes compared to traditional alternatives.^[^ ^107^, ^108^ ^]^ Cadmium‐based quantum dots, for example, have historically been applied towards bioimaging, diagnostics and drug delivery; yet the use of this heavy metal has been shown to cause severe organ toxicity and is under increasing scrutiny by regulatory bodies.^[^ ^109^ ^]^ Among the carbonaceous nanomaterials developed to date, carbon quantum dots, carbon nanotubes and graphene are frequently used for bioconjugation. We will expand on these substrates accordingly.

##### Carbon Quantum Dots

Carbon quantum dots (CQDs) are quasi‐spherical particles with a carbon‐based core often containing both sp^2^ and sp^3^ hybridized carbon atoms in an amorphous‐crystalline structure. These dots can absorb and emit light across a wide range of spectra: absorption spans the UV to visible light range, while emission spans the visible to NIR range. Some CQDs are also capable of excitation‐independent fluorescence and two‐photon absorption or emission, which can be leveraged for advanced medical imaging systems. Properties are influenced by factors such as particle size, surface functionalization, and synthesis method.^[^ ^110^, ^111^ ^]^ For example, CQDs exhibit the “quantum confinement effect”, a phenomenon that becomes significant when particle size approaches or falls below the materials’ dielectric constant. This effect leads to an increase in band gap energy and the emergence of discrete energy levels as particle size decreases, enabling the precise tuning of electronic and optical behavior for outcomes such as photocatalysis, photothermal effects and altered fluorescence.^[^ ^112^ ^]^ While the quantum confinement effect is shared by all quantum dots, CQDs offer additional advantages such as their biocompatible/eco‐friendly/sustainable composition (referenced earlier) and the presence of rich surface functionalities including carboxyl, hydroxyl and amine groups when materials such as carbohydrates, proteins, or biomass are used as precursors. Given the combination of optimal bioconjugation conditions and unique properties, CQD conjugates have been employed towards a range of medical applications.

These properties make CQDs particularly effective in the development of biosensors. For instance, Hamd‐Ghadareh et al. used CQD conjugates for the ultrasensitive detection of microRNA‐155 (miR‐155), a key biomarker for cancer diagnosis. The sensor worked by leveraging CQD substrates capable of producing two distinct fluorescent emissions (489 nm and 665 nm) upon excitation by UV light. Using EDC/NHS, CQDs were conjugated with “probe microRNA” complementary to the target microRNA, alongside the fluorescent dye Rhodamine B to serve as an internal control. Co‐diffusion with gold nanoparticles resulted in the specific quenching of CQD fluorescence at the 489 nm wavelength, a result of FRET likely supported by physisorptive interactions between the gold nanoparticles and the probe microRNA, enabling sufficient physical proximity for energy transfer. When target microRNA was introduced to the system, 489 nm fluorescence was restored as a result of hybridization between the target and probe strands, replacing close contact with the gold nanoparticles. In this way, the ratio of 480 nm / 580 nm fluorescence could be used to infer levels of miR‐155 present, demonstrating a linear relationship between ratio and miR‐155 concentration over a broad range (1 aM to 0.1 µM), with an ultralow limit of detection (0.3 aM). This was tested on human sera and cancerous cell lines (MCF‐7, MDA‐MB‐231).^[^ ^113^ ^]^

CQDs have also been utilized as drug delivery agents for payloads such as chemotherapy drugs doxorubicin and methotrexate, and the antioxidant boldine.^[^ ^114^, ^115^, ^116^ ^]^ The CQDs offer an increased surface area that helps widen the therapeutic window by efficiently packing targeting and controlled‐release molecules to enhance drug delivery. For example, Yang et al. conjugated CQDs with NLS peptide through EDC/NHS chemistry before adding doxorubicin (DOX) through the formation of a hydrazone bond, which employed pH‐sensitive hydrazinobenzoic acid as a linker. This dual functionalization provided both targeting and the pH‐sensitive release of DOX in acidic tumor environments. Cellular uptake and nucleus localization were measured by leveraging the natural fluorescent properties of CQDs in combination with confocal laser scanning microscopy and flow cytometry. The conjugates demonstrated efficient cellular uptake, nucleus localization, and enhanced apoptosis induction in A549 lung cancer cells compared to free DOX. While apoptosis increased by ca. 71% relative to the free DOX, necrosis decreased by a relative ca. 57%, suggesting a more precise and less toxic mechanism. In vivo mice studies similarly showed superior tumor inhibition and reduced systemic toxicity compared to free DOX, highlighting the therapeutic potential for CQD conjugates over traditional chemotherapeutic regimes.^[^ ^114^ ^]^

##### Carbon Nanotubes

Carbon nanotubes (CNTs) are hollow cylinders made from rolled graphene sheets, consisting primarily of sp^2^ bonds with minimal sp^3^ bonds or π‐π interactions. Depending on the number of overlapping cylinders, CNTs can be classified as single‐walled (SWCNTs), double‐walled (DWCNTs), triple‐walled (TWCNTs) or multi‐walled (MWCNTs). These nanomaterials are characterized by diameters of 100 nm or less that are accompanied by high aspect ratios, causing them to behave as “1D materials” with high surface areas.^[^ ^117^ ^]^ Their cylindrical structure and strong sp^2^ bonds confer high tensile strength, and their hollow nature means CNTs can be loaded with chemotherapy drugs and other payloads. In addition to cell labeling and tumor targeting applications,^[^ ^118^ ^]^ which are shared by many of the substrates covered in this review, CNTs are often applied towards gene therapy through functionalization with siRNA, antisense oligonucleotides, DNA, and other nucleic acids.^[^ ^119^ ^]^ They have also been used for antiviral therapeutics, as in the case of Rodriguez‐Pérez et al.

Rodriguez‐Pérez et al. functionalized several classes of carbon nanomaterial with carbohydrates to inhibit Ebola virus infection. Among the substrates used, multi‐walled carbon nanotubes produced the best results. One set of MWCNTs was decorated with glycodendrons (dendritic carbohydrate chains), while another set was decorated with glycofullerenes containing mannose and galactose. Conjugation in both cases was completed by first functionalizing the MWCNTs with phenylactylene groups via a Tour reaction before attaching the respective ligands using CuAAC. The therapy's mode of action involved competitively binding DC‐SIGN (dendritic cell‐specific intercellular adhesion molecule‐3‐grabbing non‐integrin) receptors on dendritic cells, which mediate viral entry through Ebola glycan recognition. By blocking these receptors, the conjugates demonstrated high inhibitory potency against Ebola virus, with glycofullerene‐MWCNTs producing inhibitory concentration 50% (IC_50_) values as low as 0.37 µg/mL. In theory, these glycan mimics benefit from non‐immunogenicity, facile scale‐up, and broad‐spectrum antiviral applications compared to competitive inhibitors such as antigens, which are typically virus‐specific. The conjugates outperformed free glycans for one primary reason: conjugation to the high‐surface‐area nanotubes enabled the presentation of multiple carbohydrate moieties in a dense, multivalent array, increasing the likelihood of successful inhibition. MWCNTs also mimic viral capsids due to their structural similarities.^[^ ^120^ ^]^

In a paper by Chen et al., AFM tips were modified with MWCNTs to synthesize cell nanoinjectors for the precise delivery of cancer therapeutics. The MWCNTs were functionalized with biotin via a pyrene‐based linker molecule, which attached to the substrate via π‐π stacking with the nanotubes’ aromatic rings. The other end of the linker attached to the biotin through a disulfide bond. Quantum dots manufactured with a streptavidin coating were then paired to the biotinylated nanotubes. The resulting conjugate was injected into the cytosol of HeLa cells via AFM, where native reducing agents cleaved the linker's disulfide bond, releasing the quantum dot. Successful delivery of QDs was confirmed by measuring fluorescence intensity relative to untreated cells, demonstrating the potential for future therapeutic injections.^[^ ^121^ ^]^

MWCNTs have also been used to produce sensitive, low‐cost, and rapid biosensors leveraging the high affinity and specificity of aptamers for the detection of thrombin (implicated in blood clotting disorders). As described by Kara et al., MWCNTs were oxidized to introduce carboxylic groups prior to drop‐casting onto a screen‐printed carbon electrode (SPCE). 5′‐NH_2_ modified thrombin‐binding aptamer (TBA) was then covalently bound to the nanotubes using EDC/NHS coupling, with the modification ensuring preservation of the aptamer's ability to bind. The resulting “aptasensor” operated via electrochemical impedance spectroscopy (EIS), wherein changes to charge transfer resistance could be detected upon thrombin binding to the aptamer. MWCNTs were thus selected as a result of their charge‐transfer capabilities and the power to enhance immobilization through their high surface areas. The sensor demonstrated a detection limit of 105 pM, a linear detection range of 0.39 to 1.95 nM, a relative standard deviation of 7.5%, and high specificity in the presence of other proteins. Its label‐free nature and the use of low‐cost materials further enhanced the potential for widespread applications in clinical diagnostics and point‐of‐care testing.^[^ ^122^ ^]^

##### Graphene

Graphene is a two‐dimensional nanomaterial composed of a single layer of sp^2^ carbon atoms arranged in a hexagonal honeycomb lattice, and it can exist in many forms: pristine graphene (its pure form), graphene oxide (GO), reduced graphene oxide (rGO), and graphene quantum dots (GQDs – fluorescent fragments of graphene that exhibit quantum confinement effects similar to CQDs).^[^ ^123^ ^]^ Graphene is characterized by excellent thermal and electrical conductivity and other unique properties (for example: its antimicrobial activity has received increasing attention in recent years), making it an excellent fit for biomedical conjugation.^[^ ^124^, ^125^ ^]^

**Figure 14:**
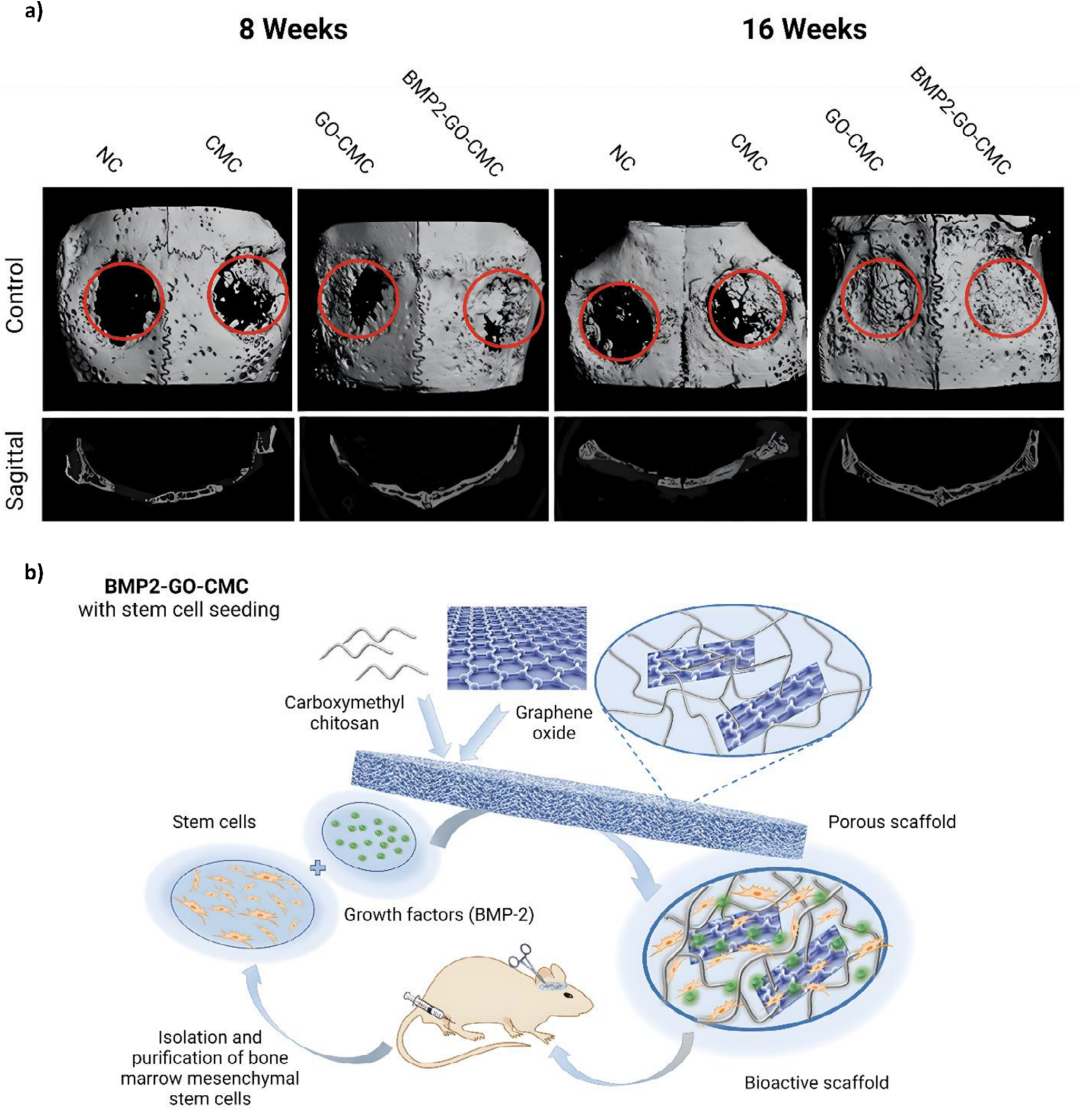
a) Comparison of bone tissue repair after 8 and 16 weeks using a negative control (NC), lone CMC, CMC conjugated to GO, and CMC and BMP‐2 conjugated to GO. As can be seen, the conjugates (and particularly the dual conjugate) significantly accelerate regeneration. b) Schematic illustrating the formation of dual conjugate BMP2‐GO‐CMC combined with stem cell seeding. Adapted with permission.^[^ ^126^ ^]^ Copyright 2016, WILEY‐VCH Verlag GmbH & Co. KGaA, Weinheim.

Ruan et al. highlighted the attractiveness of graphene oxide sheets in bone tissue engineering thanks to other key properties such as mechanical strength (mimicking the stiffness of natural bone tissue), π‐π interactions, hydrophilicity, high surface area, and the abundance of functional groups. GO carboxyl groups were used to attach carboxylmethyl chitosan (CMC) ligands, water‐soluble derivatives of chitosan, through the latter's amine groups using EDC/NHS. The same approach was used to attach a second ligand comprising bone morphogenetic protein‐2 (BMP‐2), a key osteoinductive growth factor, for its sustained release and bioavailability. As a result, water loss decreased from 120% to 44% when comparing unmodified CMC scaffolds to bioconjugate scaffolds. In addition to GO's hydrophilicity, these findings were attributed to synergistic effects from CMC's hydrophilicity and steric hindrance formed upon bioconjugation, effectively trapping water molecules within the conjugate microstructure and slowing their evaporation. π‐π interactions from the GO sheets resulted in a striking upregulation of osteogenesis‐related genes such as osteopontin, bone sialoprotein, osterix, osteocalcin, and alkaline phosphatase. Reparative effects were measured in rat models with critically‐sized skull defects, with BV/TV (bone volume to tissue volume) ratios as high as 54.8% for the BMP2‐GO‐CMC conjugates after 16 weeks, relative to 28.6% for GO‐CMC, 11% for solitary CMC, and 6.3% for a negative control indicating natural repair (**Figure** **14**). When combined with stem cell seeding, these impressive results could be expected to grow even further.^[^ ^126^ ^]^

Although the above examples use several different conjugation techniques, carbonaceous nanomaterials are primarily conjugated using EDC/NHS due to their abundant surface carboxyl groups, noting that the exact composition depends on the synthesis method and precursors used. Physisorptive π‐π interactions are also frequently used due to the aromatic nature of carbonaceous nanomaterials.^[^ ^127^, ^128^ ^]^ Interestingly, materials such as carbon nanotubes are often conjugated with multi‐component ligands containing nanoparticles, perhaps due to their high tensile strength.

#### 2.3.4 Characteristics and Challenges of Synthetic Substrate Conjugation

Unlike hybrid nanoparticles, synthetic substrates benefit from predictable surface properties that facilitate characterization and scale‐up.^[^ ^129^ ^]^ However, many surfaces lack functional groups such as those found on biological substrates, or they are less stable in biological environments, and as a result, conjugation often requires chemical pre‐functionalization (or in some cases, physisorptive layers). This can be seen in many of the above examples, which are characterized by larger functional radii thanks to the addition of silica and polymer coatings, native ligand extensions, and linkers (often introducing functional groups that can be activated via EDC/NHS). As a result, penetration and diffusion may be reduced, and hepatic uptake may increase.^[^ ^130^ ^]^ On the other hand, larger functional radii can improve targeting specificity due to improved ligand surface area. It is therefore important to weigh the degree of pre‐functionalization required against the intended applications, as this may impact the desired core nanoparticle size.

### 2.4 Summary of conjugation by substrate

**Figure 15:**
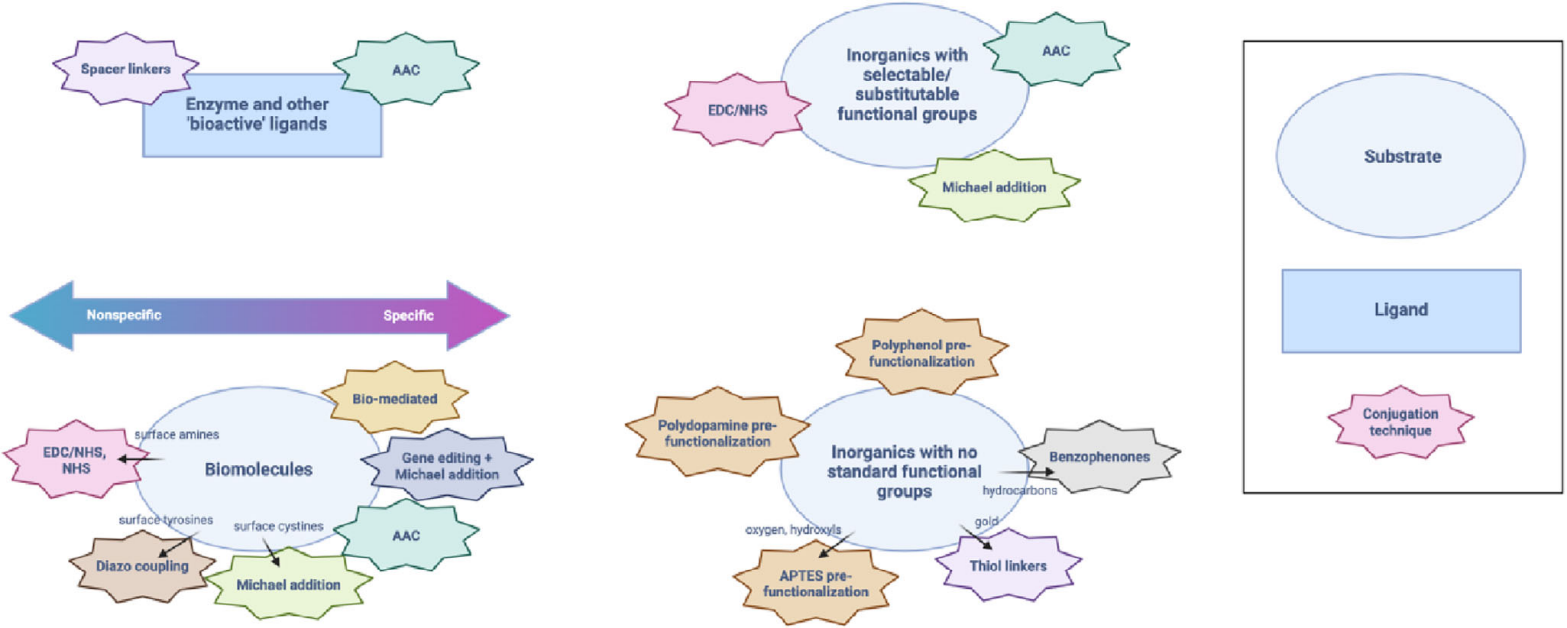
Graphic depicting conjugation approaches for material types at large. Note that some of the approaches specified for “inorganics with no standard functional groups” are not expanded upon in this review. Approaches used for biomolecules are grouped so those providing higher specificity are located to the right. The top left corner applies to ligands; the remaining figures apply to substrates.

The spectrum of fascinating materials described above perfectly encapsulates why conjugation has expanded to fill so many gaps. Surface functional groups continue to play an important role when approaching conjugation, as seen in conjugate literature at large. However, characteristics such as the presence of active sites, the manner in which the material is prepared, and the types of ligands associated with the material are often equally important to the decision‐making process. As a general rule, bio‐mediated approaches are a good fit for biological substrates that are sensitive to malplaced modifications. Alternatively, genetic modification to rare moieties such as cysteines can be paired with more common approaches such as Michael addition if native cysteines are not conveniently placed. The same is true for azide‐alkyne click chemistry. Genetic modification or incorporation is well‐suited to materials that would be grown in plasmids anyway, such as recombinant spider silk. The synthesis procedure is especially relevant to materials such as COFs, MOFs and UCNPs, where specific functional groups can be included or swapped for simple conjugation (e.g., EDC/NHS, Michael addition, or AAC). Inorganic materials often lack these groups and thus require “priming” (for example, APTES can be used to attach amines to oxide surfaces). Across all three material categories, substrates that employ enzymes and other “active” biomolecules as their ligands often use bioorthogonal AAC and/or flexible “spacer” linkers, which better preserve functional integrity. For the same reason, physisorption may be used in applications that are less dynamic and therefore less likely to pull the ligand from its substrate (e.g., benchtop diagnostics). These patterns, which derive from the examples above and literature as a whole, are summarized below (**Figure** **15**). For readers specifically looking to conjugate the materials described, the accompanying table may be of more use (**Table** **1**). It should be noted that while this review describes general trends, this does not imply that these trends are always the best course of action to take. They can, however, provide a starting point for conjugate design.

**Table 1:** Summary of relevant material characteristics and conjugation approaches commonly used for each substrate discussed in this review.

Substrate	Relevant characteristics	Common conjugation approaches
Viral vectors	Active/sensitive substrate Predictable surface composition Surface thiols Surface amines Surface phenols (some plant vectors)	Genetic modification + Michael addition Michael addition EDC/NHS Diazo coupling (some plant vectors)
Gene‐editing complexes	Active/sensitive substrate Less frequently conjugated	No clear pattern; bio‐mediated and AAC examples
Live cells	Active/sensitive substrate Surface amines	NHS and other ester‐based (nonspecific) Bio‐mediated (specific)
Spider silk	Platform substrate Enzyme ligands Methionine replacement to AHA well‐tolerated	AAC
Cellulose	Platform substrate Surface hydroxyls	Oxidation + EDC/NHS and other chemistries AAC
Gold‐decorated	Binds to thiols Negative charge in aqueous media	Dative bonding Dative bonding + EDC/NHS (linkers) Physisorption
Hybrid nanozymes	Enzyme ligands Diverse material group	No clear pattern; spacer linkers commonly used
COFs	Less dynamic environments Enzyme ligands Carboxyl, amine monomers Thiol monomers Azide monomers	EDC/NHS Michael CuAAC Physisorption
MOFs	Carboxyl, amine organic linkers Azide organic linkers	EDC/NHS CuAAC
Piezoelectrics	Surface oxygen (BTO)	APTES functionalization + NHS and other ester‐based (+ Michael addition)
UCNPs	Native or substituted amines, carboxyls Native or substituted thiols Native or substituted hydroxyls	EDC/NHS Michael addition APTES functionalization + linker or co‐precip. + AAC
Carbonaceous	Surface carboxyls Aromatics	EDC/NHS Pi‐pi physisorption

## 3 Characterization and analysis

The distinct properties of novel bioconjugate materials give rise to better therapeutic and diagnostic outcomes and a number of unique applications. But prior to investigating key outcomes, it is important to first characterize the synthesized bioconjugates. Although nuances exist (particularly with respect to biological and hybrid substrates), both novel and more traditional bioconjugates are studied using the same toolbox. Many techniques have been developed for the characterization and analysis of bioconjugates, and these have been covered by several in‐depth reviews.^[^ ^131^, ^132^, ^133^ ^]^ However, reviews are typically grouped according to instrument type. By grouping according to the characteristic being measured, we hope to capture the broad scope of analysis and allow readers to quickly find the options available for measuring a given characteristic. This also allows us to directly compare the efficacy of different techniques with the same function.

### 3.1 Confirmation and quantification of bioconjugation

After performing a bioconjugation reaction, it is imperative to confirm whether the ligand has bound at all and then in the manner intended. Surface coverage is a common metric to look for, as it allows researchers to optimize ligands to a density that keeps moieties accessible without limiting the odds of interaction—while also influencing diffusion speed, colloidal stability, and circulation time.^[^ ^134^ ^]^ Bioconjugation can be confirmed and quantified through many different approaches, so we will describe the most common and reliable ones before briefly highlighting other methods, many of which are best suited as complementary data.

Bioconjugation can be proven through both direct and indirect means. Indirect methods involve quantifying unbound ligand from supernatant and subtracting this from a known input concentration. Any protein quantification assay can work for this purpose, for example bicinchoninic acid (**BCA**), **Bradford** or enzyme‐linked immunosorbent (**ELISA**) assays. Due to its specific nature, ELISA represents the only viable option when blocking proteins such as BSA are added to prevent aggregation and instability.^[^ ^135^ ^]^ This is because BCA and Bradford detect overall protein content, making it impossible to distinguish between conjugating ligand proteins and BSA in the supernatant. UV‐vis can also be used to correlate biomolecule absorption against a known concentration curve.^[^ ^131^ ^]^ Unfortunately, indirect methods are prone to overestimation due to events such as unbound ligand agglomeration and container adsorption going undetected. Antibody conjugation to gold nanoparticles is reportedly overestimated by 18–434% when indirect methods are used for quantification.^[^ ^135^ ^]^ However, indirect quantification is virtually the only approach that can be used for all bioconjugates, which is perhaps why it is so common. It is important to note that supernatant ingredients can interfere with assays and may need to be adjusted post‐conjugation—either through centrifugation, buffer exchange, or dilution (keeping biomolecule concentration within assay limits). In the case of BCA assays: free thiols, ascorbate, and reducing sugars reduce copper (overestimating results and underestimating conjugation), while EDTA, citrate, and imidazole chelate copper (underestimating results and overestimating conjugation). Acidic buffers can neutralize the alkaline working environment, also underestimating results.^[^ ^136^ ^]^ Because conjugation can reduce ligands’ ability to interact with assay molecules and nanoparticles can scatter light, it is difficult to apply these assays directly onto conjugated particles—although it can be done by subtracting matrix absorbance.^[^ ^137^ ^]^ Semiquantitative **Southern, Northern and Western blots**, which indicate target ligand through signal changes (typically colorimetric/fluorescent), can be used directly^[^ ^138^, ^139^, ^140^ ^]^—however, this approach is less common.

Direct methods, which analyze the conjugate itself, are generally considered more reliable. One common approach is to use dynamic light scattering (**DLS**), a technique which correlates the intensity of scattered light with the hydrodynamic diameter of particles in solution.^[^ ^141^ ^]^ An expected increase in diameter, based on the size and expected coverage of ligands, can indicate successful conjugation. While this increase has been used to approximate the number of bound ligands using a Taylor dispersion analysis model,^[^ ^142^ ^]^ it is difficult to accurately predict coverage. Many DLS devices are also capable of measuring **zeta potential** (the effective charge on a particle surface), which can indicate bioconjugation via a shift in charge distribution^[^ ^143^ ^]^ and has been used to accurately quantify BSA attachment to gold nanoparticles.^[^ ^144^ ^]^ Nonetheless, zeta potential is sensitive to variable factors such as pH and ionic strength meaning data is not typically reliable enough to stand on its own.^[^ ^145^ ^]^

Like DLS, nanoparticle tracking analysis (**NTA**) measures changes in hydrodynamic diameter. This is accomplished by tracking the Brownian motion of individual particles under laser illumination with the help of a microscope and video camera.^[^ ^146^ ^]^ Curve‐fitting models can again be used to estimate ligand density.^[^ ^147^ ^]^ DLS measurements are generally simpler, faster and offer a wide size range (approximately 0.3 nm‐10 µm),^[^ ^148^ ^]^ while NTA (10 nm‐1 µm)^[^ ^149^ ^]^ provides higher resolution data and is better suited for polydisperse and agglomerating samples, as it is less biased towards larger particles.^[^ ^150^ ^]^ NTA devices can also quantify particle concentration and, when equipped with the right accessories, fluorescence. Thus NTA can be used to calculate average conjugation efficiency of particles with fluorescently‐labelled ligands.^[^ ^151^ ^]^ Fluorescent ligands can also be quantified using a standalone **fluorimeter** (measures average fluorescence) or **nanoparticle‐dedicated/modified flow cytometer** (measures individual particles >40–100 nm),^[^ ^152^, ^153^ ^]^ with the potential for imaging under a **fluorescent microscope** if bioconjugates are larger than ca. 200 nm.^[^ ^154^ ^]^ While fluorescent methods can accurately indicate successful conjugation, like DLS and NTA they are less reliable when it comes to estimating conjugation efficiency due to fluorescence quenching and enhancement caused by nanoparticles, particularly in the case of metal nanoparticles. Quenching typically occurs with aggregation or when fluorophores bind too close to the nanoparticle surface, encouraging electron or other energy transfer between fluorophore/nanoparticle or fluorophore/fluorophore (from anywhere between ca. 1 and 10 nm distance).^[^ ^155^ ^]^ On the other hand, overestimation can occur when metal nanoparticles influence fluorophore excitation and emission rates through their local electromagnetic field.^[^ ^156^ ^]^ To get around this problem, nanoparticles are sometimes **dissolved** in order to quantify the previously bound fluorophores without interference. Reagents such as cyanide are used for gold and silver nanoparticles.^[^ ^157^, ^158^ ^]^ It is difficult to dissolve other materials because the strong acids and bases required to do so can denature ligands and fluorophores themselves.

A phenomenon that has been widely exploited in nanotechnology is localized surface plasmon resonance (LSPR). The effect occurs when incident light is trapped within conductive nanoparticles smaller than the wavelength of the light, causing electrons on the particle surface to oscillate and form a localized electromagnetic field with distinct optical properties.^[^ ^159^ ^]^ LSPR absorption peaks are shown to red‐shift in the presence of biomolecules, thus **UV‐vis** represents another common probe for gold and silver nanoparticle conjugation.^[^ ^160^, ^161^ ^]^ While effective and non‐invasive, this method requires a thorough knowledge of adhered ligands’ refractive indices.^[^ ^135^ ^]^ Refractive index refers to the way in which light propagates through a medium and can be influenced by surface coverage, orientation and water content. For this reason it is difficult to quantify ligand density, although it has been done.^[^ ^157^ ^]^ In a few cases, UV‐vis has been used to confirm and even quantify conjugation without the help of LSPR, but rather through protein and DNA absorption bands around 185–320 nm^[^ ^162^, ^163^ ^]^ and 260 nm^[^ ^164^ ^]^ respectively, in addition to 410 nm in the case of hemoproteins.^[^ ^165^ ^]^ The infrequent use of this technique is likely due to biomolecules generally having a low absorption and concentration relative to their respective nanoparticles (in the case of synthetic and most hybrid substrates).^[^ ^166^ ^]^

Other common probes include Fourier transform infrared (FTIR) and Raman spectroscopy, both of which provide valuable information on molecular composition. Using infrared radiation, **FTIR** can indicate successful bioconjugation through changes in the vibrational modes of particles after conjugation. This is particularly useful when working with synthetic substrates, as characteristic biomolecule peaks such as amide I bands around 1600–1700 cm^−1^ and amide II bands around 1480–1580 cm^−1^ can serve as distinct markers for proteins.^[^ ^167^ ^]^ Frustratingly, water bending overlaps with amide I peaks. In some cases it is possible to subtract a water spectra without producing too much noise, and in other cases it is useful to freeze‐dry the conjugates.^[^ ^168^ ^]^ Attenuated total reflectance (ATR)‐FTIR avoids water interference by sending light through a highly refractive index prism.^[^ ^169^ ^]^ FTIR has been used to quantify bioconjugation by comparing free ligand concentration curves against the IR absorbance intensity of conjugates, however most papers involve polymer ligands <100 nm in length due to their distinct absorption curves and the fact that excessive length can cause absorbance overlapping.^[^ ^170^ ^]^

**Figure 16:**
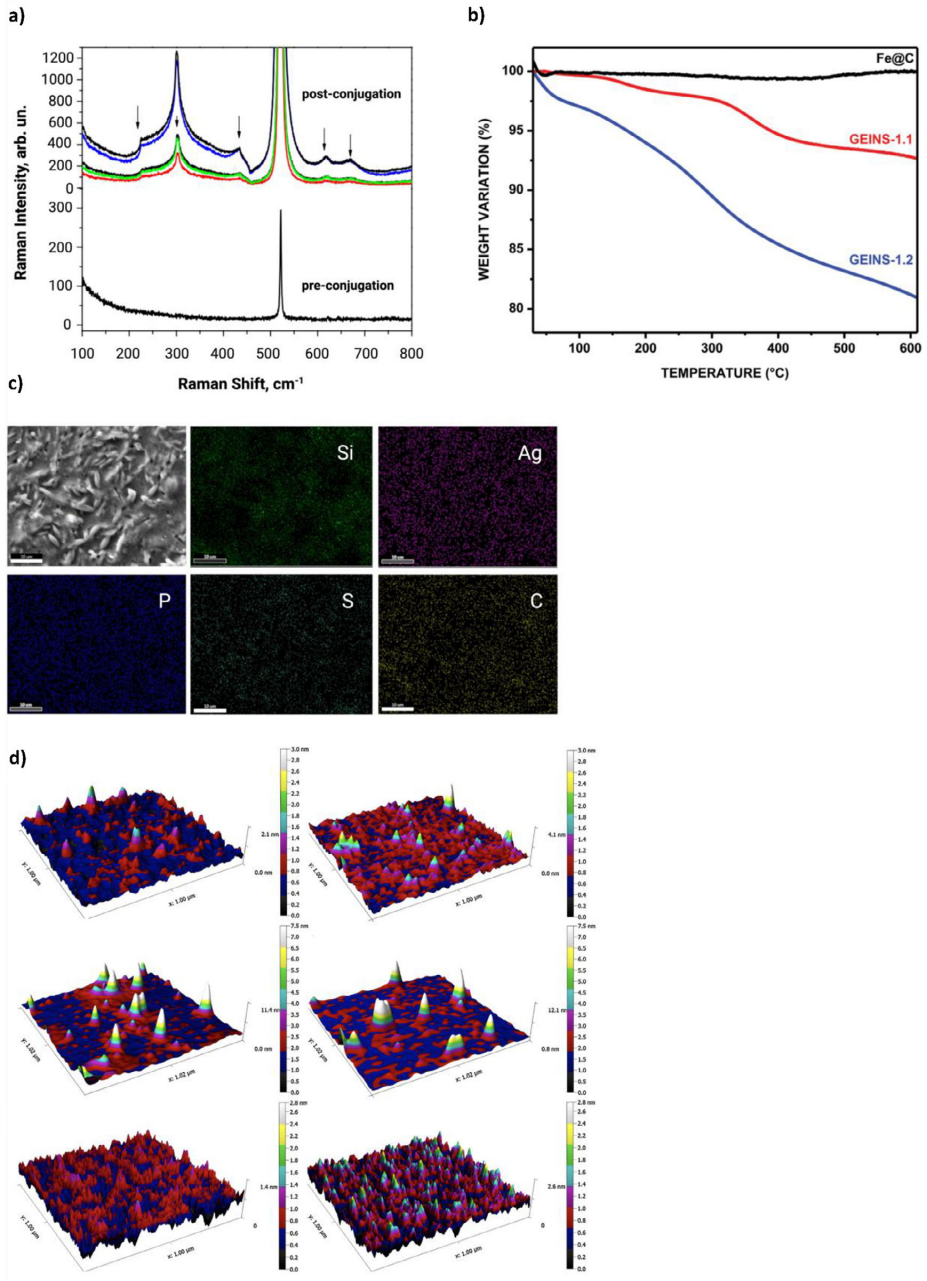
a) Raman spectra of CdSe/ZnS quantum dots before and after bioconjugation with mouse ovarian cancer antibodies. Colored lines at the top correspond to different bioconjugate samples. Adapted with permission.^[^ ^173^ ^]^ Copyright 2012, WILEY‐VCH Verlag GmbH & Co. KGaA, Weinheim. **b)** TGA curves of graphene‐coated iron nanoparticles (Fe@C, black line), Fe@C conjugated with polyethylenimine (PEI) (GEINS1.1, red line), and Fe@C conjugated with PEI under different reaction conditions to GEINS1.1, yielding a higher ligand density (GEINS2.2, blue line). Thermal decomposition of PEI begins around 220 °C and completes around 600 °C. Reproduced with permission.^[^ ^82^ ^]^ Copyright 2016, the Royal Society of Chemistry. **c)** EDX of coenzyme A‐conjugated silver nanoparticles. Top left: SEM micrograph; top middle: silicon; top right: silver; bottom left: phosphorous; bottom middle: sulfur; bottom right: carbon. Overlaps from biological elements such as phosphorous, sulfur and carbon against silver suggest successful conjugation. Reproduced with permission.^[^ ^189^ ^]^ Copyright 2023, the Royal Society of Chemistry. **d)** Left: AFM height images of APTES/succinic anhydride functionalization (top), APTES/glutaraldehyde functionalization (middle), and plasma polymerized acrylic acid (PPAA) functionalization (bottom) of silicon wafers. Right: AFM height images in which protein G is conjugated to each respective functionalized surface. Reproduced with permission.^[^ ^195^ ^]^ Copyright 2016, Elsevier B.V.

Like FTIR, **Raman spectroscopy** measures the vibrational energy of particles, although this is based on the inelastic light scattering of UV, visible and near‐IR light.^[^ ^171^ ^]^ Characteristic Raman bands can shift or intensify, and new bands can appear, in response to covalent bond formation between ligand and particle (**Figure** **16**a).^[^ ^131^, ^172^, ^173^ ^]^ In the case of gold and silver nanoparticles, surface enhanced Raman scattering (**SERS**) can be employed. This produces a more sensitive signal and can detect biomolecules at lower concentrations.^[^ ^174^ ^]^ Like FTIR, ligand density can be calculated by generating a standard curve for free ligands in solution.^[^ ^175^ ^]^ Another benefit to Raman is that water interferes with the spectra to a lesser degree than in FTIR, thus when it comes to metallic nanoparticles or aqueous samples, Raman may provide higher resolution data. In the case of non‐metallic nanoparticles, FTIR can detect bulk molecular changes with the benefit of identifying a broader range of functional groups groups.^[^ ^176^ ^]^ In light of their differences, FTIR and Raman complement each other well.

A generally cost‐effective alternative to the above methods is electrophoresis, particularly **gel electrophoresis**.^[^ ^177^ ^]^ This can be used to confirm conjugation by highlighting differences in mobility (based on size, or size and charge) between the bare and conjugated nanoparticle.^[^ ^161^ ^]^ In one case, gel electrophoresis was able to resolve the attachment of just one, two or three biomolecules,^[^ ^178^ ^]^ and in theory the colorimetric stain used (for example, Coomassie Blue) can be quantified with instruments such as a fiber optic spectrophotometer to average ligand per particle. It should be noted that in practice, bands can be difficult to distinguish when ligands are relatively small, and quantification is not as reliable as other techniques like mass spectrometry, mentioned below.^[^ ^131^ ^]^

Other less common probes include mass spectrometry (MS), resonant mass measurement (RMM), NMR, x‐ray photoelectron spectroscopy (XPS), thermogravimetric analysis (TGA), and energy‐dispersive x‐ray spectroscopy (EDX) elemental mapping. **Mass spectrometry** (particularly MALDI‐TOF, matrix‐assisted laser desorption ionization‐time of flight) can be useful for reliably correlating increased particle mass against the average concentration of bound ligands, however this technique is not commonly used for proving or quantifying conjugation – perhaps owing to its relatively high costs, required level of expertise, and the destruction of the sample.^[^ ^131^ ^]^ The more recently‐developed **RMM** offers similar capabilities by flushing nanoparticles through a resonant microfluidic channel to calculate the buoyant mass of individual particles.^[^ ^179^ ^]^ This technique can provide a more accurate range for ligand density, providing a distribution plot that closely resembles that of DLS and NTA, but for mass.^[^ ^142^ ^]^ However, RMM devices are less common.

**NMR** uses the magnetic absorption of atomic nuclei to reveal molecular structures and dynamics, meaning it can also prove binding. For example, dextran attachment to quantum dots was confirmed through the appearance of strong peaks in the alkyl region of a ^1^H NMR spectra.^[^ ^180^ ^]^ This high‐accuracy technique has been used to calculate grafting density in several cases, again by comparing conjugate spectra to unbound ligand spectra.^[^ ^181^ ^]^ **XPS** can signal the appearance or enhancement of elements on particle surfaces through signature atomic peaks (for example, sulfur or nitrogen).^[^ ^182^ ^]^ Changes in binding energy can also affect peak conformation, which can be mapped to specific covalent moieties.^[^ ^183^ ^]^ A number of transformation models and software tools are capable of deconvoluting peaks for high‐resolution scans in cases where multiple peaks overlap, and quantification is also possible.^[^ ^184^, ^185^ ^]^ However, a monolayer of particles is necessary to obtain more accurate densities, the formation of which may displace some ligands.^[^ ^185^ ^]^ **TGA**, which is often coupled with FTIR analysis, tracks sample weight across a range of temperatures and can evidence bioconjugation through more pronounced weight loss curves corresponding to the degradation of ligands at signature temperatures (Figure 16b).^[^ ^82^ ^]^ In some cases, these peaks have been used to estimate grafting density.^[^ ^186^ ^]^

Energy‐dispersive x‐ray spectroscopy (interchangeably known as **EDX**, EDS, EDXS or XEDS) can provide a more visual perspective in cases where biomolecules are being attached to an inorganic substrate. The technique uses characteristic x‐ray spectra to identify elements in a (typically) dried sample, which can be displayed as image layers with the help of SEM or TEM.^[^ ^187^ ^]^ EDX is much less sensitive than other probes such as FTIR or NMR, however color maps and spectra for elements including sulfur, phosphorous and nitrogen—assuming these elements are not also present in the nanoparticle—can contribute further evidence (Figure 16c).^[^ ^188^, ^189^ ^]^ Carbon and oxygen do not provide strong evidence of conjugation simply because many compounds contain these elements. Additionally, carbon tape is often used to deposit sample onto the instrument stubs. Hydrogen, being an extremely light element, is impossible for standard EDX detectors to identify due to its resulting low‐energy x‐rays.^[^ ^190^ ^]^

Topographical imaging techniques such as high‐performance atomic force microscopy (**AFM**) and near‐field scanning optical microscopy (**NFSOM**), the latter of which bridges AFM with fluorescence microscopy, can also be used to indicate ligands with a high degree of resolution (20‐50 nm axially, a few nanometers laterally).^[^ ^191^ ^]^ This enables longer molecules to be imaged as a rough 3D structure for a given nanoparticle surface area (Figure 16d). Additionally, AFM devices can be functionalized to map interaction forces, which can be used to distinguish between covalent, high‐affinity and low‐affinity, non‐covalent interactions.^[^ ^192^ ^]^ Many more techniques than those covered have been applied to prove successful bioconjugation,^[^ ^131^, ^193^, ^194^ ^]^ however for brevity we will limit our discussion to those already mentioned.

### 3.2 Biological and hybrid substrate considerations

As has been shown, there are a number of tools available for proving successful bioconjugation. This first degree of characterization is advisable for all bioconjugation papers, particularly those that don't incorporate bioactive ligands such as enzymes or antibodies (which can be verified through functional assays). However, some of the abovementioned techniques are limited when it comes to biological and hybrid substrates due to overlapping signals, irregular shapes and uneven surfaces. In the case of biological substrates, techniques such as BCA and Bradford cannot distinguish between conjugated and unconjugated particles. DLS, NTA and RMM are optimized for spherical particles, making these a good fit for icosahedral and other round viral capsids but a poor fit for many other biomolecules. Additionally, protein hydration shells can influence perceived hydrodynamic radii. UV‐vis is only effective where one biomolecule has a distinct absorption band outside of the overlapping region, for example if it contains a heme group. Similarly FTIR, Raman, TGA, EDX, and to an extent NMR and zeta potential all suffer from overlapping characteristics such as elemental makeup and decomposition temperatures, producing overlapping spectra. Fortunately, ELISA (where applicable), fluorescent techniques, gel electrophoresis, MS, XPS, and AFM circumvent these issues. Among these, MS is well‐suited to bio‐bio conjugates as the device can detect small concentrations and sizes of ligands. Zeta potential may be effective depending on the concentration and surface charge of ligands relative to substrates (for example, conjugating DNA to a protein can produce a notable negative shift). AFM can be challenging where biological substrates are characterized by uneven surfaces, however one workaround is to use high‐aspect‐ratio tips, which enhance access to valleys without compromising resolution.^[^ ^196^ ^]^

Hybrid surfaces such as those found on gold‐decorated nanoparticles require these adjustments to an even greater extent, as descent heights are much more pronounced (for example, 20 nm or more for a gold‐decorated, antibody‐conjugated nanoparticle in which gold nanoparticles are approximately 10 nm in diameter). Additionally, smaller metallic nanoparticles (<60 nm) have been shown to quench fluorescence more effectively, which limits their use in fluorescent devices unless fluorophores are >10 nm away from the substrate.^[^ ^197^, ^198^ ^]^ In the case of bio/synthetic hybrid substrates, bio‐bio‐compatible techniques also work. However, fully synthetic hybrid materials can employ almost all of the techniques mentioned, with the exception of fluorescent devices under the conditions just mentioned in addition to DLS, NTA and RMM if particles are significantly polydisperse and/or asymmetrical (this is also the case for synthetic substrates more generally). These considerations are relevant to device selection while measuring other characteristics such as stability and ligand orientation, described below.

**Table** **2** provides a general guide to the devices mentioned in this section, although conjugation should always be considered on a case‐to‐case basis. For example, photobleaching of inherently fluorescent ligands (as opposed to fluorescently‐tagged ligands for using fluorescent devices) has not been accounted for, and low oxidation conditions are assumed.

**Table 2:** Techniques for confirming bioconjugation. General summary of key considerations for comparing techniques that can be used to confirm bioconjugation. Note that ligands are assumed to contain some biological entity or derivative, and technique selection should always be done on a case‐by‐case basis. Techniques marked with a bolded “Yes” indicate highly‐effective quantification.

Method/device name	Destructive to bioconjugate?	Physiological liquid mediums supported?	Distribution vs average data	Conjugation quantifiable (ligand density)?	Compatible with biological substrates?	Compatible with synthetic hybrid substrates?
Indirect supernatant assay (BCA, Bradford, UV‐vis)	No	Yes	Average	Yes, considerations^k)^	Rarely^r)^	Yes
Indirect supernatant assay (ELISA)	No	Yes	Average	Yes, considerations^k)^	Yes	Yes
DLS	No	Yes	Both	Yes, considerations^l)^	Some^s)^	No
Zeta potential	No	Yes	Average	Yes, considerations^m)^	Rarely^t)^	Yes
NTA	No	Yes	Distribution	Yes	Some^u)^	No
Fluorescent NTA	No	Generally^f)^	Distribution	Yes, considerations^n)^	Some^s)^, ^u)^	Some^z)^
Fluorimeter	Potentially^a)^	Generally^f)^	Average	Yes, considerations^n)^	Some^u)^	Some^z)^
Fluorimeter + nanoparticle dissolution	Yes	Generally^f)^	Average	**Yes**	No	Some^aa)^
Nanoparticle flow cytometer	Potentially^a)^	Yes	Distribution	Yes^o)^	Yes	Some
UV‐vis via LSPR	No	Yes	Average	Yes	No^v)^	Some^bb)^
UV‐vis	No	Yes	Average	Yes	Rarely^w)^	Yes
FTIR and derivatives	Potentially^b)^	Yes^g)^	Average	Yes^p)^	Rarely	Yes
Raman spectroscopy	No^c)^	Yes	Both^i)^	Yes	Rarely	Yes
SERS	No^c)^	Yes	Both^i)^	Yes	No	Some^bb)^
Gel electrophoresis	Yes	No	N/A^j)^	Yes^o)^	Yes	Yes
Mass spectrometry	Yes	No	Average	**Yes**	Yes	Yes
RMM	No	Yes	Distribution	**Yes**	Some^s)^	Some^s)^
NMR	No	Yes	Average	**Yes**	Some^x)^	Yes
XPS	Potentially^d)^	No	Average	Yes, considerations^p)^	Yes	Yes
TGA	Yes	No	Average	Yes	No	Yes
EDX using SEM or TEM	Yes	No	N/A^i)^	No	No	Yes
AFM	Potentially^e)^	Yes	N/A^h)^	No	Some^y)^	Some^y)^
NSOM	Potentially^e)^	Generally^f)^	N/A^h)^	No	Some^y)^	Some^y)^

^a)^ Potential photobleaching

^b)^ If drying methods are used (these can potentially destabilize biomolecules)

^c)^ Assuming reasonable laser power

^d)^ Depending on sample preparation and radiofrequency power

^e)^ Depending on the tip and tip force used, in addition to imaging mode and scanning speed

^f)^ Salt may interfere

^g)^ Yes – but drying methods such as freeze‐drying are recommended if ATR‐FTIR is unavailable, meaning liquid mediums are not needed

^h)^ Visual output that cannot be classified as a distribution or average that is representative of the full sample

^i)^ If mapping is used for visual distribution data

^j)^ Average if quantitative additions are used

^k)^ Prone to overestimation

^l)^ Difficult for polydisperse particles

^m)^ Possible but difficult to control for pH, ionic strength and other effects

^n)^ Accuracy is reduced if substrates contain metal and fluorophores fall within ca. 10 nm of the metal

^o)^ Possible but uncommon

^p)^ Not when freeze‐dried

^q)^ Prone to underestimation

^r)^ No – unless the substrate lacks peptide bonds, and the ligand contains them

^s)^ If substrates are reasonably spherical

^t)^ No – unless the surface charges of the substrate and ligand differ significantly

^u)^ If ligands are fluorescent (either inherently or as a result of functionalization)

^v)^ LSPR only applies to plasmonics (metals)

^w)^ No – unless either the substrate or the ligand (but not both) contains a heme

^x)^ If higher‐resolution spectrometers and smaller substrates are used

^y)^ If substrate surface morphology is consistent or high‐aspect‐ratio tips are used

^z)^ Generally – but most reliable when substrate is not metal‐decorated, or is metal‐decorated with sufficient ligand space (e.g., >10 nm)

^aa)^ If it is possible to dissolve all substrate components without damaging the ligands

^bb)^ If ligands interact with plasmons on the substrate (e.g., if it is plasmon‐decorated)

### 3.3 Stability and conformational changes

Bioconjugate stability is important for extending shelf life, preventing agglomeration, and optimizing the biological activity of conjugating ligands, thereby preserving overall performance. Stability can be influenced by a variety of factors – namely pH, concentration, ionic strength of solution, temperature, radiation, and ligand dynamics. At the same time, conformational changes are key to bioconjugate projects which involve self‐assembly, controlled release, or enzymatic ligands, in addition to conjugates which could trigger undesired immune responses through epitope exposure.

Stability can be measured through several different approaches. A relatively straightforward technique is to measure the **zeta potential**, as particles with high absolute zeta potential (either positive or negative) are less likely to aggregate due to electrostatic repulsions. Likewise, a low relative zeta potential can be a sign that particles are aggregating. A value of at least ±30 mV is often designated as a stable range.^[^ ^131^ ^]^

Infrared spectroscopy techniques such as **FTIR** and ATR‐FTIR can show signs of protein denaturation or aggregation through changes to the signature amide I and II peaks mentioned previously. In the case of amide I (C = O) for example, stable proteins typically display a single, relatively sharp peak or a set of peaks in the 1600–1700 cm^−1^ range. The exact positioning depends on the protein's secondary structure: alpha helices and beta sheets appear around 1640–1660 cm^−1^ and 1630–1640 cm^−1^ respectively, while random secondary coils center between the two, around 1640–1650 cm^−1^. When proteins unfold, the amide I band shifts and broadens due to a loss of defined secondary structure. The unstable spectrum will often center around the 1640–1650 cm^−1^ range due to unfolding of alpha helices and beta sheets into random coils.^[^ ^199^ ^]^ FTIR can also signal protein aggregation, as shown by an increase in intensity around 1620–1630 cm^−1^, indicative of intermolecular beta‐sheet formation.^[^ ^200^ ^]^ While not strictly necessary, one drawback to FTIR is that it often requires an understanding of what a stable spectrum looks like for comparison against a potentially unstable one. Circular dichroism (**CD**) spectroscopy is akin to a more detailed version of FTIR in that it can detect subtle changes to the secondary and even tertiary structures of biomolecules. By measuring the differential absorption of polarized light, this technique provides detailed insights into the conformational state of biomolecules in real time, indicating molecular interactions and events such as unfolding and melting (for which onset temperature can be recorded).^[^ ^201^ ^]^

Amino acid **fluorescence** can also be used to infer tertiary structure. Tryptophan is sensitive to changes in its local environment such as enhanced polarity. This can occur when proteins denature and the tryptophan residues are turned away from hydrophobic pockets, exposing them to the surrounding solvent. The decrease in stability typically results in a fluorescent redshift^[^ ^202^ ^]^ and can also signal desired protein unfolding, for example if the conjugate involves pH‐stimulated drug release or if ligands are more interactive (e.g., antibodies, DNA or enzymes).

While heat tolerance profiles are known for more common biomolecules, **TGA** can provide information on the exact temperatures at which ligands begin to break down, informing storage conditions and applications. The generated thermogram can indicate the onset temperature of decomposition (and other events such as oxidation and moisture evaporation) through signature changes in mass. Importantly, TGA can also distinguish between ligand and substrate decomposition.^[^ ^203^ ^]^ Most TGA furnaces operate between 25–1100 °C,^[^ ^204^ ^]^ fitting the majority of proteins which begin to denature around 50–70 °C.^[^ ^205^ ^]^ Another heating technique is differential scanning calorimetry (**DSC**), which records heat flow as a function of temperature.^[^ ^206^ ^]^ Unfolding events appear as distinct peaks in the thermogram and can be used to understand the thermal stability of conjugates and the energy required to destabilize them (denaturation enthalpy, Δ*_d_H*, or the area under the peaks). Some papers have gone a step further by fitting thermodynamic models to the curve to better describe individual unfolding events.^[^ ^207^, ^208^ ^]^ While TGA and DSC are useful for understanding bioconjugate stability under different temperature profiles, they do not provide the same degree of conformational data as techniques such as FTIR and CD.

Though less common, **UV‐vis** can be applied for metallic materials such as gold nanoparticles, where aggregation is associated with absorption spectra redshifts greater than the expected range for ligand attachment. One paper optimizing the stability of gold nanoparticle‐antibody conjugates used an “aggregation index” equation where a ratio of peak 525 nm absorbance (LSPR) to peak 585 nm absorbance (a significant redshift) indicated aggregation if greater than 1.^[^ ^209^ ^]^

As touched on, an important aspect of bioconjugate conformation is the accessibility of active sites with respect to target molecules. This is sometimes referred to as “catalytic stability”.^[^ ^210^ ^]^ Given the wide array of potential ligand‐target interactions, we will not describe this measure of stability in much depth here, other than to note that a variety of kinetic models (e.g., Michaelis‐Menten) and colorimetric, fluorescent and other assays (e.g., ELISA, standalone TMB) may be used to characterize the catalytic activity of bioconjugates under different conditions. A number of reviews delve into these measures further.^[^ ^211^, ^212^, ^213^, ^214^, ^215^ ^]^

### 3.4 Ligand orientation

**Figure 17:**
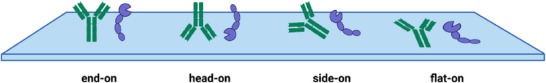
Schematic illustrating the various ligand orientations as they are referred to in this section. Created in Martin, C. (2025) https://BioRender.com/ibx5ioe.

Due to the precise nature of characterizing ligand orientation, a limited number of techniques are used. **DLS** and **NTA** lend themselves to the task in cases where ligands are reasonably monodisperse but asymmetric in some way (for example the IgG antibody, which measures approximately 14.2 nm in height and 8.5 nm in width).^[^ ^216^ ^]^ By comparing the increase in hydrodynamic diameter against ligand dimensions, one can roughly estimate ligand orientation provided the substrate is relatively monodisperse and colloidally stable (although NTA is slightly more forgiving).^[^ ^217^, ^218^ ^]^ For example, Ruiz et al. found that a solute pH of 7.5 produced an NTA increase of 13.2 ± 1.1 nm in anti‐HRP‐adsorbed gold nanoparticles at monolayer coverage. In contrast, pH values of 8 and 8.5 corresponded to just 9.8 ± 1.0 nm and 7.4 ± 0.6 nm respectively, suggesting a more upright orientation at 7.5 which was further confirmed through an immunoassay.^[^ ^218^ ^]^ It should be noted that “layer thickness” techniques are ideally performed by comparing surface‐functionalized molecules to fully conjugated molecules in order to account for at least some of the diameter increase. These methods are particularly useful if the conjugation chemistry permits a limited number of orientations, as this can help distinguish between “up” and “down” (e.g., end‐on versus head‐on antibody orientation) (**Figure** **17**). While DLS and NTA are typically used to confirm bioconjugation rather than orientation, their working principal is shared with techniques such as neutron reflectivity and AFM, described below, which together with time‐of‐flight secondary ion mass spectrometry (ToF‐SIMS) are routinely used.

Another technique which shares the DLS/NTA working principle is the **quartz crystal microbalance (QCM)**, which can estimate layer thickness by monitoring small changes in ligand mass through a piezoelectric quartz sensor on which nanoparticles are adhered. This data is translated to thickness using a Voigt viscoelastic model and has been used to estimate the orientation of DNA, proteins and other conjugating ligands.^[^ ^219^, ^220^, ^221^, ^222^ ^]^ **Neutron reflectivity** or neutron reflection (NR) measures the angle at which a beam of neutrons reflects off solid‐liquid interfaces, providing direct information about the structures there. Measurements occur at the angstrom level and can inform the thickness and chemical composition of layers around the surface in question, with a thickness boundary that varies between 5‐5000 Å, or 0.5‐500 nm.^[^ ^223^ ^]^ It is therefore unsurprising that this technique is frequently used to characterize orientation. Following the logic of DLS, NTA and QCM, a number of studies have differentiated between side, flat‐lying and upright orientations by fitting “depth profile” models to NR data, comparing estimated layer height against ligand dimensions.^[^ ^224^, ^225^, ^226^, ^227^, ^228^ ^]^

**AFM** can also be used to this end. The device operates under ambient conditions and is arguably the most direct way to infer orientation given that it can visualize surface topography at the nanometer scale. In most cases, the surface of the conjugate is recreated on a planar glass slide for ease of analysis, however this runs the risk of misrepresenting the molecular dynamics of the actual conjugates. In answer to this, some studies have transferred their fully‐conjugated complexes onto the slide instead. This is the case for Marciello et al., who validated the orientation of lipase‐ and antibody‐coated magnetic nanoparticles both directly, through AFM projection height, and indirectly through a peroxidase immunoassay.^[^ ^229^ ^]^ Both methods suggested a standing, or end‐on, orientation. The process can be automated by setting an upper and lower height boundary to isolate potential ligands. Due to the vertical force applied by the AFM probe during imaging, it is common for ligand protrusions to appear shorter than expected, particularly those oriented vertically.^[^ ^230^, ^231^ ^]^ Keeping this in mind, the presence of “lobes” (e.g., 2 lobes for F_ab_ regions and 1 lobe for F_ac_) can be a useful visual metric to validate findings. For example, three lobes can indicate a flat‐on antibody.^[^ ^230^ ^]^ Probe distortions can also be corrected for using standards such as small gold colloids. By analyzing colloids of a known and relevant size, researchers can predict and calibrate for size discrepancies. It should also be noted that smooth or at least topologically‐uniform substrates make it easier to distinguish between surface and ligand. Regardless, software that takes stock of the surface plane can be a useful way to correct for subtle variations in tilt and height that could obscure or exaggerate the height of attached biomolecules.^[^ ^232^ ^]^ Even when adjusting for all of these considerations, interactions between the AFM cantilever and weakly‐held ligands can cause movement or distortion, thus it is recommended to use “soft” cantilevers with a spring constant (k) ≤ 0.5 N·m^−1^ (>10 N·m^−1^ for conventional tapping mode);^[^ ^233^ ^]^ sharp tips (≤ 10 nm radii);^[^ ^234^ ^]^ slow scan rates (ca. 1–4 Hz); low peak forces (≤ 50–100 pN);^[^ ^233^ ^]^ and PeakForce or conventional tapping mode for highest resolution.^[^ ^233^ ^]^ Biological samples are usually placed in liquid cells.^[^ ^231^ ^]^

A simpler tool is **zeta potential**, which takes a different approach. Again using antibodies as an example, the slipping plane (theoretical boundary which zeta potential measures) will experience a larger positive shift when F_ab_ regions are oriented away from the particle, increasing the net zeta potential.^[^ ^209^ ^]^ This is due to the exposure of more positively‐charged amino acid residues, meaning a good understanding of charge distribution is necessary to interpret results. Said caveat is perhaps why zeta potential is not commonly used for orientation applications: a wide variety of factors such as ligand density, ionic strength of medium, and substrate material can influence ligand surface charge.^[^ ^145^, ^235^ ^]^ A distantly related technique is **ToF‐SIMS**, a form of mass spectrometry that is capable of analyzing the outer 2–3 nm of a surface.^[^ ^236^ ^]^ This is accomplished by firing a high‐energy beam at the sample, displacing ions which are reported according to their mass and charge.^[^ ^237^ ^]^ Characteristic mass‐to‐charge ratios (typically logged in a database) can be used to identify specific amino acids in the outermost layer, indicating which ligand regions might be outward‐facing.^[^ ^238^, ^239^, ^240^ ^]^ A thorough knowledge of amino acid composition is therefore necessary for this technique.

It is also possible to infer orientation by testing ligands' ability to interact with their target molecules through various **functional assays** (e.g., immunoassays or blocking assays). However in some cases (for example, when a substrate is less densely conjugated), access to a moiety does not precisely inform how outwards‐facing it may be. It is therefore useful to couple these indirect assays with one or several of the more direct methods mentioned above. In fact, this is a common tactic.

Antibodies are perhaps the best example of a ligand that needs to be oriented correctly, given that Fab access is critical to antigen binding. Four methods are used to optimize antibody orientation into an upright position. The first is genetic modification of the Fc region for site‐specific conjugation (e.g., engineered cysteines or an AviTag for enzymatic biotinylation). The second applies to IgG antibodies from major mammalian species, where post‐translational glycosylation enriches the F_c_ region with glycans that can be selectively targeted. It is common to oxidize the glycans with sodium periodate to create aldehydes, which react with hydrazides in a manner that can be stabilized by sodium cyanoborohydride. Hydrazide linkers are typically employed for this step (e.g., hydrazide‐thiol linkers for gold attachment).^[^ ^135^ ^]^ A third approach that works on both polyclonal and monoclonal antibodies from various mammalian species is the incorporation of F_c_‐binding proteins A and G. These proteins are naturally produced on bacterial cell walls in order to “flip” nearby antibodies, shielding their Fc regions from immune recognition.^[^ ^241^ ^]^ While protein A/G‐F_c_ interactions are characterized by dissociation constants as low as 10^−7^ and 10^−8^, attachment is still reversible and therefore less durable than covalent bonds.^[^ ^242^ ^]^ A fourth, less precise option involves modifying pH before or during conjugation (particularly physisorptive conjugation) to induce favorable charge conditions for Fc attachment. This was shown by Ruiz et al. (mentioned previously), where a buffer pH of 7.5 was shown to enhance Fc orientation during electrostatic adsorption to gold nanoparticles.^[^ ^218^ ^]^ Genetic modification, “patch” (e.g., glycan) chemistry, and pH modulation can also be applied to other sensitive ligands for their controlled orientation if properties are well‐known. For example, enzyme ligands can be engineered with N‐ or C‐terminal tags if the chosen terminus is not essential for function and is spatially distant from the active site.

### 3.5 Surface Charge

Another important metric is surface charge, given its influence on cellular uptake (specific and nonspecific), circulation time, and colloidal stability. A bioconjugate's electrostatics are typically measured using **zeta potential** ^[^ ^243^, ^244^, ^245^, ^246^, ^247^ ^]^ (usually performed via a DLS machine with ELS, or zeta potential, capabilities). Zeta potential specifically measures the electrical potential at the slipping (“shear”) plane of a particle. This plane occurs just beyond the surface of the particle, in the region where tightly‐bound ions transition to freely‐moving ions in the surrounding solution. Positive and negative potentials are determined by identifying the direction of particle movement during electrophoresis.^[^ ^248^ ^]^ **Kelvin probe force microscopy (KPFM)** has been used in a few cases to map the direct surface potential of bioconjugates,^[^ ^249^, ^250^ ^]^ as the device works by measuring the local electrostatic potential between a conductive AFM tip and the sample surface, although sudden changes in terrain height can reduce the accuracy of this technique.^[^ ^251^ ^]^ Ligands approximately 50 nm and below (i.e., most ligands) produce sufficient contrast.^[^ ^252^ ^]^ Surface potential and charge distribution can also be computationally modelled based on known material properties, ligand sequences and solution properties,^[^ ^253^ ^]^ however we will not expand on this method here. Zeta potential is generally better suited to understanding bulk behaviors such as colloidal stability, whereas surface potential and charge distribution are more useful for surface‐specific interactions such as receptor binding.

### 3.6 Biocompatibility and Cytotoxicity

Viability assays can be used to assess the biological toxicity of conjugates and their individual components. However, nanoparticle substrates can interfere with assays, confounding readouts. It is therefore crucial to understand nanoparticles’ physical, chemical, and optical properties before conducting assays. Common assays such as MTT (3‐(4,5‐dimethylthiazol‐2‐yl)‐2,5‐diphenyltetrazolium bromide) and MTS (3‐(4,5‐dimethylthiazol‐2‐yl)‐5‐(3‐carboxymethoxyphenyl)‐2‐(4‐sulfophenyl)‐2H‐tetrazolium) are typically read on a microplate **spectrophotometer**.^[^ ^254^ ^]^

**Figure 18:**
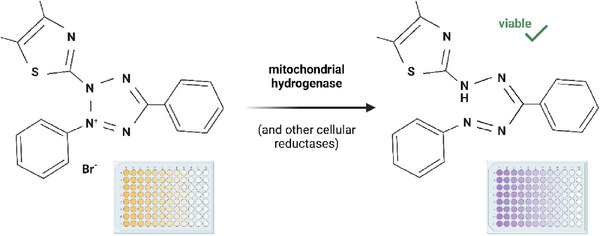
MTT reduction by mitochondrial hydrogenase and other cellular reductases, leading to the formation of the formazan product (and indicating cell viability via a color change from yellow to purple). Created in Martin, C. (2025) https://BioRender.com/ydqpyig.

MTT and MTS are colorimetric methods used to indicate cellular viability and proliferation within microplates by assessing metabolic activity. MTT is a positively‐charged tetrazolium salt that penetrates the cell membranes of viable eukaryotic cells and is reduced by mitochondrial dehydrogenases, producing insoluble purple formazan crystals (Figure 17) (please place Figure 18 here and remove (Figure 17) as written before!). MTS is negatively‐charged and cannot penetrate cell membranes; it is instead reduced by electron carriers such as NADH or NADPH to produce soluble purple formazan crystals. MTT's crystals are dissolved using dimethyl sulfoxide (DMSO) before reading absorbance values. While unnecessary, MTS assays often utilize DMSO for consistency.^[^ ^255^ ^]^ Equation 1 is used to determine viability in the case of both assays:

(1) $$\mathrm{Cell}\;\mathrm{viability}\left(\%\right)=\frac{\mathrm{Abs}\left(\mathrm{sample}\right)\;-\mathrm{Abs}\left(\mathrm{blank}\right)}{\mathrm{Abs}\left(\mathrm{control}\right)\;-\mathrm{Abs}\left(\mathrm{blank}\right)}\times 100$$

Both assays are influenced by cell culture medium and supplements including serum albumin and fatty acids. Huang et al. found that serum albumin binds to the reduced formazan product of MTS assays, reducing absorbance readings by up to 50%. This effect was modulated by the addition of fatty acids, which competed with formazan for albumin binding to restore partial absorbance. The study emphasized the importance of managing these interactions to improve the reliability of MTS assays, noting that defined media with optimized components may help address these issues. Interestingly, many cell culture protocols, including those from the UK Health Security Agency (UKHSA) and the American Tissue Culture Collection (ATCC), recommend using 10% fetal bovine serum – which contains high levels of serum albumin and variable fatty acids (**Figure** **18**).^[^ ^256^ ^]^

While MTS and MTT assays are commonly used to assess biocompatibility and cytotoxicity, other spectrophotometer assays such as Alamar Blue, CellTiter‐Glo, and trypan blue can be found in the literature. Flow cytometry provides insights into cellular uptake and accumulation and has been used to indicate necrosis and apoptosis in vitro.^[^ ^257^, ^258^ ^]^ Staining techniques employing Acridine Orange, Ethidium Bromide, and Calcein AM provide information about conjugate interactions with various cell lines, with Calcein AM being used most often. In this approach, nonfluorescent Calcein AM dye diffuses across cell membranes into viable cells. Once inside, intracellular esterases cleave the acetoxymethyl (AM) group, converting Calcein AM into the green fluorescent molecule Calcein. Both molecules are hydrophobic and stay localized to the cytoplasm, enabling precise and reliable fluorescent measurements. As a result, Calcein AM assays have been widely applied to evaluate cell viability, adhesion, apoptosis, and cytotoxicity. Results can be quantified with fluorescence microplate readers or visualized with fluorescence microscopy.^[^ ^259^ ^]^

**Summary of multi‐capability devices** (**Table** **3**)

**Table 3:** Summary of devices capable of more than one of the characterization methods mentioned in this section.

Method/device name	Characteristics measured
Zeta potential^*)^	Confirmation of conjugation; Stability; Ligand orientation; Charge distribution
DLS*, NTA	Confirmation of conjugation; Ligand orientation
FTIR	Confirmation of conjugation; Stability and conformational changes
Fluorimeter	Confirmation of conjugation (fluorophores and amino acid fluorescence); Stability and conformational changes (amino acid fluorescence)
TGA	Confirmation of conjugation; Stability
UV‐vis	Confirmation of conjugation; Stability

*) Often included on the same device

## 4 Perspectives

Bioconjugation is inherently synergistic. This is especially true when novel materials are used as substrates, conferring functional properties that are enhanced or complemented by ligands to achieve the desired use case. Many other novel materials can therefore be expected to infiltrate bioconjugate literature in the future, particularly those that are stimuli‐responsive or biodegradable. Combination therapeutics have been increasingly reported through the use of multiple ligands, multi‐component ligands and drug encapsulation, and this trend will likely continue, although complexity will need to be carefully balanced against scalability. Fortunately, recent innovations to monoclonal antibody production have significantly reduced costs,^[^ ^260^ ^]^ and the same is true of peptide synthesis.^[^ ^261^ ^]^ As batch ligand production becomes more affordable, bioconjugates can be expected to move from the lab to the commercial scale with more potential for regulatory approval. At the same time, synthetic ligands are already cost‐effective, scalable and relatively predictable and may be amplified as a result.^[^ ^262^ ^]^ Substrate, ligand and bioconjugation technique selection will likely face additional pressure from a sustainability perspective. For a better understanding of how bioconjugate regulation will look in the future, it is first necessary to describe its history and current status.

The FDA approved its first regulated antibody‐drug conjugate in 2000. “Mylotarg” was composed of monoclonal anti‐CD33 covalently bound to the antibiotic “calicheamicin” via an acid‐labile hydrazone linker. The compound targeted acute myeloid leukemia and was approved through the FDA's accelerated program based on promising early clinical data. The drug was withdrawn in 2010 after confirmatory trials identified toxicity concerns and failed to demonstrate a significant survival benefit. Mylotarg was re‐approved in 2017 after adjustments to the dosing regimen and patient groups, and a host of bioconjugates have been approved by regulatory bodies since its inception (primarily antibody‐drug conjugates and PEGylated biomolecules).^[^ ^263^, ^264^ ^]^ Still, Mylotarg's journey provides insights into the challenges that arise in demonstrating quality, safety and efficacy when synthesizing and characterizing bioconjugates.^[^ ^265^ ^]^

Before approval of any bioconjugate for market release, stringent quality control assessments must be conducted. These include characterization techniques described in this review (for example: proof of conjugation, ligand density, ligand orientation, stability, and biocompatibility) alongside methods to determine the efficiency of the conjugation reaction and by extension, purity.^[^ ^266^ ^]^ In addition to the conjugates themselves, it is important to characterize clinical outcomes through measures such as specificity, LOD, LOQ, and reproducibility.^[^ ^267^ ^]^ While bodies such as the FDA and the European Medicines Agency (EMA) clarify that bioconjugate and clinical outcome assessments need to be conducted with “justified acceptance criteria”, they do not provide any standardized guidance on which instruments and techniques to use. Currently, the World Health Organization (WHO) provides the most detailed guidance, for example by recommending the use of NMR to prove conjugation of pneumococcal polysaccharide‐protein vaccines. WHO documents state that “if a national regulatory authority so desires, these Recommendations may be adopted as definitive national requirements, or modifications may be justified and made by the regulatory authority”.^[^ ^268^ ^]^ Documents such as the target product profile (TPP), technical specification series (TSS), preferred product characteristic (PCC) and the EU‐mandated summary of product characteristics (SmPC) can also guide product design with regulatory expectations in mind.^[^ ^269^, ^270^ ^]^

The International Council for Harmonization of Technical Requirements for Pharmaceuticals for Human Use (ICH) is a global initiative that brings together all major regulatory bodies and pharmaceutical industry members, and in 2022 the ICH published a harmonized “M10” document specifying drug characteristics to be measured by applicants across all regulatory bodies. While regulatory harmonization is promising, the M10 lacks any discussion on characterization techniques and their relative effectiveness. Considering the WHO is already publishing specific recommendations and regulatory bodies such as Health Canada have stressed the need to develop clearer guidelines,^[^ ^271^ ^]^ it is possible that frameworks will become standardized to a greater extent in the future (timeline unclear), particularly as bioconjugate research continues to expand. In order for this to happen, regulatory bodies will need to collaborate with industry stakeholders and academics so that key considerations and best practices can be shared. Until then, onus remains on the researcher to thoroughly characterize their bioconjugates and to choose starting materials that will result in effective, predictable, scalable and safe applications for the greater good.

In addition to reflecting on future materials and the regulatory landscape, it is worth commenting on applications for artificial intelligence. The 2024 Nobel Prize in Chemistry was awarded to researchers David Baker, Demis Hassabis and John Jumper for their use of AI in protein design and structure prediction, highlighting its ability to distill the inherent complexity that exists at the molecular level.^[^ ^272^ ^]^ Bioconjugation can be difficult to optimize as a result of this complexity, leading to suboptimal conversion efficiencies and unstable or ineffective products. AI and particularly deep learning thus lends itself to this task by modelling material properties, simulating conjugate performance, selecting conjugation strategies, and improving reaction efficiencies. For example: x‐ray crystallography and cryo‐EM are gold standard techniques for inferring protein structure, but they are intensive and structures are not always available on Protein Data Bank (PDB). As a workaround, protein sequences can be fed into AlphaFold, RoseTTAFold and similar AI models to predict 3D structures and visualize accessible functional groups on protein substrates and ligands. In many cases, sequences can be obtained from sites such as UniProt and do not need to be individually obtained by the researcher.

Physical models such as density functional theory (DFT), molecular dynamics (MD), quantitative structure‐activity relationship (QSAR) and AutoDock can serve as training datasets for neural network potentials and Gaussian process regression models, producing AI surrogates that retain accuracy at significantly lower computational costs.^[^ ^273^ ^]^ Both surrogates and data‐trained AI can predict properties such as surface functionalization, toxicity and therapeutic activity with greater cross‐comparison than physical models alone, even when the latter is deployed on high‐performance computational clusters (HPCCs).^[^ ^274^ ^]^ For example, Chen et al. developed ‘ADCNet’ to predict whether 435 antibody‐drug conjugates were therapeutically active using a combination of ESM‐2 (a protein language AI model), FG‐BERT (a small‐molecule representation AI model), and drug‐to‐antibody ratio (DAR) values. ADCNet predicted activity with ca. 87% accuracy.^[^ ^275^ ^]^

Beyond predictive modeling, AI has been used to optimize experimental parameters through empirical training datasets. Zhang et al. used artificial neural network (ANN) and genetic algorithm (GA) AI models to optimize EDC/NHS concentrations and coupling times for cellulase bioconjugation to smart polymer Eudragit L‐100, producing conjugation efficiencies of ca. 88%.^[^ ^276^ ^]^ While the field continues to gain traction, challenges such as limited data quality and quantity (leading to potential biases in training datasets) could hinder its effectiveness. Nevertheless, careful application could virtually transform the field, paralleling the trajectory seen in computer aided drug design (CADD).

## 5 Conclusions 

To demonstrate the range of biomedical applications associated with novel substrates, this review has explored the conjugation of viral vectors and capsids, gene‐editing complexes, live cells, spider silk proteins, cellulose, gold‐decorated nanoparticles, nanozymes, covalent‐organic frameworks, metal‐organic frameworks, piezoelectrics, upconversion nanoparticles, and carbonaceous nanoparticles. In each case, conjugating ligands were used to enhance the unique properties provided by the substrate, demonstrating bioconjugation's ability to produce highly effective, synergistic properties at the small scale. Conjugation is often associated with targeted cancer therapy (a trend reflected in several papers here), yet its applications extend far beyond. This review covered topics such as increasing the sensitivity of diagnostic assays; improving the fidelity of gene therapy; studying cell receptors and enzymes in real time; engineering difficult tissue structures; promoting sustainable tissue engineering; enhancing optogenetic safety; prolonging prodrug therapy; and inhibiting viral infection using scalable materials. In each case, these interesting applications were enabled or enhanced by the unique properties of the materials used to achieve them.

While bioconjugation methods such as EDC/NHS, Michael addition and azide‐alkyne click chemistry are commonly employed, the field is constantly expanding, with new techniques often driven by novel substrates as described in this review. For example, enzyme‐, protein‐ and aptamer‐mediated approaches are highly specific and perform well in biological environments, making them well‐suited to functionalizing biological substrates (alongside azide‐alkyne). Standard techniques such as Michael addition can be paired with genetic modification to achieve similar levels of specificity. Notably, all three approaches tend to require genetic modification of the substrate and sometimes the ligand, with some notable exceptions (e.g., aptamer‐ and certain protein‐ and enzyme‐mediated techniques). Plasmid constructs are typically used, although vectors may be necessary for in‐vivo and other intensive systems. Needless to say, precision usually begets complexity. Careful substrate selection can sometimes circumvent this if amino acids or other reactive moieties are uncommon and well‐placed, although this requires detailed knowledge of the target's sequence, structure, and surface accessibility. EDC/NHS and NHS‐based approaches can be applied to a broad spectrum of materials with no modification required, but they are generally limited to non‐selective applications such as fiber decoration and cell imaging, and may still affect function. In contrast, synthetic materials often lack key functional groups and require priming through the attachment of coupling agents or linkers. MOFs and UCNPs are an exception to this rule: they can be engineered to contain specific functional groups. The use of priming agents and linkers may contribute to slow diffusion and hepatic uptake due to larger functional radii, although this may benefit more bioactive ligands such as enzymes by providing flexibility.

Because most applications require specific nanoscale configurations and properties, it is useful to characterize bioconjugates after their synthesis. Traits such as proof of conjugation, ligand density, stability and conformational changes, ligand orientation, surface charge, and biocompatibility and cytotoxicity can be characterized through a variety of approaches described in this review, many of which can be used for biological and hybrid substrates as well. By comparing each approach, we hope to aid researchers in choosing an appropriate set of tools to understand their conjugates on a case‐by‐case basis. This detailed comparison can also facilitate regulatory application processes, although standardized guidance may become available in the foreseeable future. With increasingly scalable ligand production costs, the development of an exciting array of new substrate materials and conjugation methods, and the advent of AI, the future of bioconjugation continues to look bright.

## Conflict of Interest

The authors declare no conflict of interest.

## Permission to reproduce material from other sources

Permission to reproduce figures from published sources has been obtained where necessary. Proper attribution has been provided in the figure captions.

## Supporting information



Supporting Information
