# Supplementary material for: Novel Bioconjugate Materials: Synthesis, Characterization and Medical Applications
Source: Adv Healthc Mater. 2025 Oct 23;14(31):e00303. doi: 10.1002/adhm.202500303 (PMC12683229; doi:10.1002/adhm.202500303)
Supplement: Supplementary file 1 — Supporting Information [file ADHM-14-0-s001.pdf]

ADVANCED HEALTHCARE MATERIALS

Supporting Information

for *Adv. Healthcare Mater.*, DOI 10.1002/adhm.202500303

Novel Bioconjugate Materials: Synthesis, Characterization and Medical Applications

*Ellie Martin, Sean P. Doidge, Eiman Aleem, Suela Kellici, Steven Dunn, Claire Atkinson and Philip D. Howes**

Supplementary Information

Novel Bioconjugate Materials: Synthesis, Characterization and Medical Applications

Authors: Ellie Martin, Sean P. Doidge, Eiman Aleem, Suela Kellici, Steven Dunn, Claire Atkinson, Philip D. Howes

1. Introduction

Here we present an overview of the bioconjugation approaches that are relevant to the works presented in the main review article, along with a glossary of terms used. An extensive number of bioconjugation techniques have been developed since the 1950s, and each reaction (or interaction) comes with a set of distinct traits that can inform whether it will be a good fit for the substrate, ligand, and application in mind. Techniques can roughly be grouped into chemical coupling, click chemistry, biorecognition, and physisorption (Figure S1). We will describe each category below, however due to the broad scope of bioconjugation we will mainly focus on commonly-used examples for each category. These are employed by the various papers highlighted in 'Novel substrates and applications' under the main text.

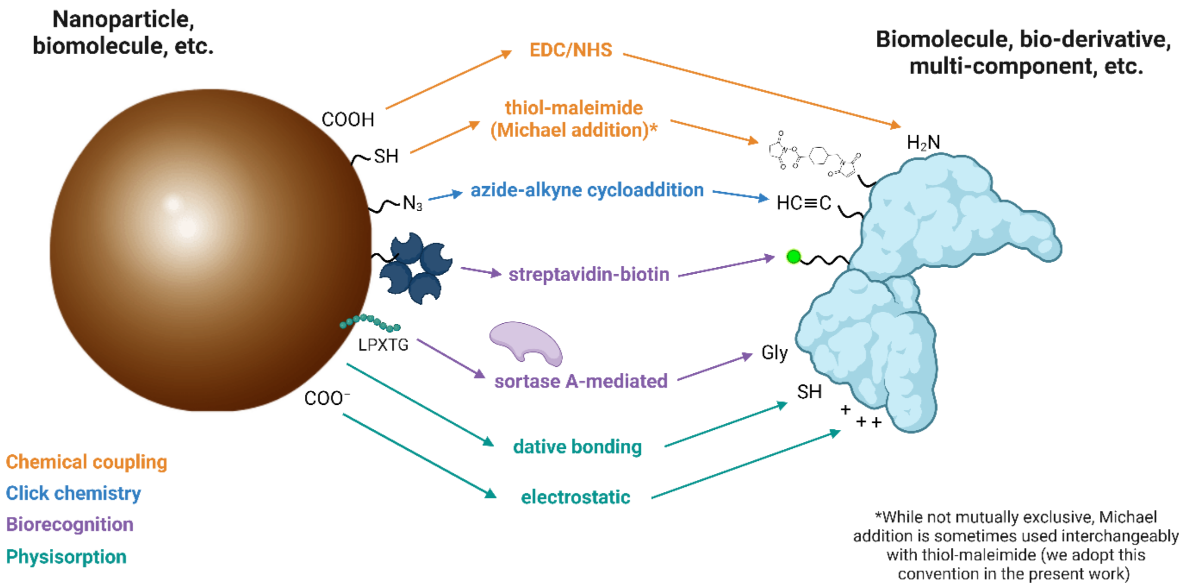

Figure S1: Schematic depicting the four general categories of bioconjugation described below, with specific examples to illustrate each category.

2. Chemical Coupling

Chemical coupling encompasses bioconjugation reactions that link two functional groups together through the formation of a covalent bond, relying on naturally-occurring moieties such as thiols,

amines, and carboxylic acids. Given the relative abundance of these groups, chemical coupling reactions are generally nonselective but require less pre-functionalization than categories such as click chemistry and biorecognition, making them well suited to applications where the exact placement of the linkage is less important. Below we will expand on conjugation methods involving thiol groups (namely, thiol-maleimide and thiol-amine reactions), and conjugation methods involving amines and carboxylic acids.

2.1. Thiol-based

Thiol-based bioconjugation methods are popular for tagging a variety of ligands to nanoparticles and other substrates. One of the simplest thiol-based methods is dative bonding, in which thiol groups covalently bind gold surfaces without the help of additional reagents.[1] Other approaches are slightly more complex; these include thiol-maleimide, thiol-amine, thiol-halogen, and thiol-pyridine disulfide reactions. Functionalization with the thiol groups themselves can be performed using several approaches, and either the substrate or the ligand may be thiolated depending on the circumstance. Proteins and peptides contain cysteine amino acids that sometimes provide free thiol groups, however cysteines more commonly contain disulfide bridges that can be converted into thiol groups through reducing reagents such as DTT ('Cleland's reagent').[2] Nanoparticles with surface hydroxyl groups, such as silica nanoparticles, can be modified with thiol-ending silane reagents (e.g. 3-mercaptopropyl)trimethoxysilane, or MPTMS) to present covalently-bound thiol groups for conjugation.[3] We will expand on thiol-maleimide and thiol-amine chemistries below, covering ways to functionalize surfaces with both maleimides and amines respectively.

2.2. Thiol-maleimide

Thiol-maleimide is a popular conjugation reaction that occurs rapidly and with high yield, forming a strong thioether bond. This reaction optimally occurs within a slightly acidic pH and is a type of Michael addition.[4] Although the terms are not mutually exclusive, many papers use thiol-maleimide conjugation and Michael addition interchangeably, and we will adopt this convention in the present work. A standard approach for introducing maleimide groups to substrates and ligands is through the use of heterobifunctional crosslinkers that contain maleimide on one end and a reactive group on the other. SMCC is one widely used example, as its NHS reactive group easily binds to protein amine groups.[3]

2.3. Amine-based

Carbodiimide coupling is a well-established technique for linking amine and carboxylic acid groups to form an amide bond. This makes it ideal for bioconjugation, as proteins and other biomolecules often contain one or both of these functional groups. Among the different coupling systems, carbodiimides such as 1-ethyl-3-(3-dimethylaminopropyl)carbodiimide (EDC) and dicyclohexylcarbodiimide (DCC) are commonly used, often with N-hydroxysuccinimide (NHS) as a coupling enhancer. EDC/NHS carbodiimide coupling is one of the most widely-used bioconjugation reactions to date, and it works by forming a heterobifunctional linker between the two functional groups. The reaction between EDC and carboxyl groups (on the surface of a nanoparticle, for example) leads to an unstable o-Acylisourea intermediate, which then interacts with NHS to form an amine-reactive ester. This ester subsequently reacts with an amine group (on the surface of a protein, for example) to generate a bioconjugate and isourea. If the intermediate does not encounter an amine group, it will be hydrolyzed back into a carboxylic acid functional group.[5] Another variant of amine-carboxylic acid conjugation is NHS esterification, which is akin to a modular version of EDC/NHS coupling. In the first step, a carbodiimide (such as EDC) is used to attach NHS to the ligand of interest. The NHS-ligand is then introduced to the solution containing the target substrate, where it can bind to amine groups. This differs from EDC/NHS where the two reagents are typically introduced at the same time.[6]

2.4. Click Chemistry

"Click chemistry" describes a class of chemical reactions that are rapid, selective, high-yielding, and occur under mild conditions.[7,8] Examples of these reactions include azide-alkyne cycloaddition, alkyne-nitrone cycloaddition, tetrazine-based inverse electron demand Diels-Alder, and isonitrile-based [4+1] cycloaddition.[9,10] Among these, copper-catalyzed and strain-promoted azide-alkyne cycloaddition are popular bioconjugation techniques. This is primarily because they are bioorthogonal, meaning they can proceed in biological environments without affecting biomolecule function. Bioorthogonality results from the high specificity of both reactions: azides react only with alkynes under appropriate conditions, avoiding side interactions with the functional groups that are typically found on biomolecules (azides and alkynes are not naturally-occurring).

2.4.1. Copper-catalyzed azide-alkyne 1,3 dipolar cycloaddition (CuAAC)

As its name suggests, copper-catalyzed azide-alkyne 1,3 dipolar cycloaddition (CuAAC) is a bioorthogonal click chemistry reaction that covalently links azide ($R\text{-}N_3$) and alkyne (R'–C≡C–R'') functional groups with the help of a Cu^{2+} or Cu^{0} catalyst. It occurs in the presence of a reducing agent such as sodium ascorbate, hydrazine, or hydroxylamine, which converts the copper catalyst into its active form (Cu^{+}).[11]

The CuAAC reaction begins with the azide donating a pair of electrons to the copper catalyst ("coordination"), forming a nitrogen-copper bond in which the resulting complex activates the azide. The alkyne group then coordinates with the copper end of the complex, forming a copper-acetylide intermediate. Now in close proximity, the two functional groups react through a [3 + 2] cycloaddition, forming a five-ring triazoline intermediate. Due to strain and instability, the ring structure undergoes a rearrangement that breaks the copper-nitrogen bond, releasing the 1,2,3 triazole product that contains bound azide and alkyne groups.[12–14]

On top of general click chemistry benefits such as high yield and the ability to proceed under mild conditions, CuAAC is highly versatile and allows for the conjugation of a wide range of molecules, forming diverse products with tailored properties.

2.4.2. Strain-promoted azide-alkyne cycloaddition (SPAAC)

Strain-promoted azide-alkyne cycloaddition (SPAAC) is another type of bioorthogonal click chemistry reaction, but unlike CuAAC it does not require metal catalysts. The inherent instability of strained alkynes causes their triple bonds to be highly reactive, enabling [3 + 2] cycloaddition and the formation of a stable five-ring triazoline under ambient conditions.[15] This approach offers superior biorthogonality to CuAAC, as metal catalysts can damage biomolecules (e.g. through ROS generation) and have the potential to affect metabolic pathways.[16] CuAAC's risk of damage is relatively low when conjugation is performed in vitro and catalyst can be quickly removed, however select applications require in vivo bioconjugation and would therefore benefit from SPAAC. The SPAAC reaction was first described in 2004[17] and has since made significant contributions to the field of bioconjugation, such as bolstering the development of bio-bio complexes.[18–20]

2.5. Biorecognition

Biorecognition techniques involve functionalizing a substrate with a biomolecule/derivative (if not already present) that can participate in highly-specific interactions with another biomolecule/derivative. Many of these techniques are relatively novel and are recognized for their precision and stability. Examples include biotin-streptavidin interactions, enzyme-mediated conjugation, protein-mediated conjugation (e.g. HaloTag and SpyCatcher/SpyTag), and aptamer-mediated conjugation. We will expand on the first three categories below, as these are more commonly used.

2.5.1. Biotin-streptavidin

Streptavidin is a ca. 56 kDa homotetramer protein[21] with a high affinity for biotin, a ca. 0.244 kDa molecule.[22] The dissociation constant between the two entities approximates 10^-14 M, making biotin-streptavidin one of the strongest known non-covalent interactions. This binding is largely unaffected by buffer solution, pH or temperature variations, making it a popular method for bioconjugation across various applications.[23] Interestingly, biotin-streptavidin conjugation often requires additional conjugation steps to functionalize both the substrate and ligand. For example, nanoparticles may be functionalized with streptavidin using EDC/NHS, while ligands are often functionalized with NHS-biotin, using NHS esterification to create the biotinylated ligand. Once functionalized, biotin-streptavidin binding proceeds without additional reagents, linking the ligand to the streptavidin-functionalized nanoparticle (Figure S2). The reverse approach is also common (e.g., nanoparticle biotinylation followed by conjugation to streptavidin-tagged ligands). While this multi-step approach may seem unnecessary compared to more direct routes, it offers several benefits. For example, direct conjugation techniques such as thiol-maleimide and EDC/NHS can be spatially constrained when key functional groups on the substrate or ligand (or both) are inaccessible. In contrast, biotin and streptavidin are independently tagged using a variety of conjugation methods, eliminating the need for complementary functional groups and providing more control over the ligand's orientation. The stability provided by the biotin-streptavidin bridge is also useful, particularly when conjugates will be traveling through dynamic environments. Additionally, biotin and streptavidin are sometimes naturally expressed (or can be genetically introduced) on biomolecules such as enzymes and live cells, removing the need for pre-functionalization.

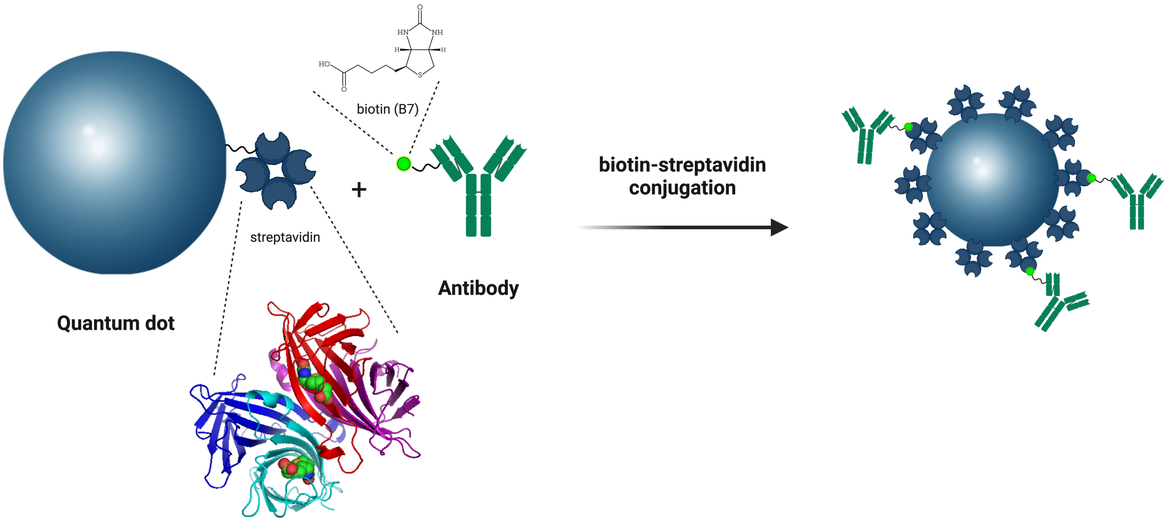

Figure S2: Illustration of biotin-streptavidin conjugation using streptavidin-functionalized quantum dots and biotinylated antibodies as an example. As can be seen, high-affinity conjugation proceeds with no additional reagents (besides those used to functionalize the substrate and ligand in prior steps). Adapted under the terms of the CC BY license.[295] Copyright 2021, the Authors.

2.5.2. Enzyme-mediated

Enzyme-mediated bioconjugation leverages the inherent specificity of enzymes to catalyze covalent bond formation between two entities under mild conditions. This approach offers more control over the conjugation site, which is particularly useful when conjugating functional particles such as live cells, other enzymes, or gene-editing complexes. One commonly used enzyme is transglutaminase (TGase), which catalyzes the formation of an amide bond between a glutamine residue (acting as the acyl donor) and a lysine residue (acting as the acyl acceptor), forming γ-glutamyl-ε-lysine.[24] Both residues naturally occur in proteins, peptides and some cell walls,[25] or they can be engineered onto the substrate

or ligand of interest. Thus, TGase can be used to form a stable covalent bond that can be region-specific, particularly if recombinant surfaces are used. Microbial TGase (mTGase) preferentially modifies accessible (e.g. solvent-exposed) glutamine residues, invoking its preferential use in bioconjugation applications.[24] Other commonly-used enzymes include sortase A, lipoic acid ligase, and tyrosinase.[26,27] Each enzyme-mediated technique requires a recognition point (e.g. residue or peptide) on the substrate, but not all enzymes carry the ligand to be attached.

2.5.3. Protein-mediated

Protein-mediated conjugation shares many overlaps with enzyme-mediated conjugation, and several protein tag systems are in fact derived from enzymes (and could therefore be considered a subset of enzyme-mediated conjugation). The same principle applies, however the recognition point is recognized by a protein 'tag' which reacts to form a covalent bond with it, eliminating the need for a third party such as an enzyme. In this way, substrates can be functionalized with the recognition point, and ligands can be functionalized with the active protein tag. The most common protein-mediated techniques include HaloTag, SNAP-tag, CLIP-tag, and SpyCatcher/SpyTag.[28] None of these techniques' recognition points are naturally-occurring, even in biological substrates, meaning protein-mediated reactions are extraordinarily specific.

HaloTag comprizes a derivative of the bacterial enzyme haloalkane dehalogenase, which normally catalyzes the cleavage of haloalkanes but in this case is modified to replace its catalytic activity with the formation of a single covalent bond. This enables conjugation of HaloTag-functionalized ligands to haloalkane-functionalized substrates.[29] SpyCatcher/SpyTag uses a short "SpyTag" recognition peptide (~13 amino acids) derived from a bacterial fibronectin-binding protein, which is first expressed on the target substrate. This is typically done using vector engineering in the case of biological substrates. Its corresponding "SpyCatcher" protein (~138 amino acids) is separately attached to the functional ligand of interest (either genetically or through chemical conjugation), after which it covalently binds its SpyTag, completing the conjugation.[30] Each method is inherently flexible in that the recognition point can be bound to the ligand rather than the substrate, and vice versa for the reactive protein, however the reactive protein is often paired with the smaller of the two for diffusion reasons.

2.6. Physisorption

A simple and cost-effective form of conjugation is the non-covalent adsorption of biomolecules through a variety of interactions with the substrate surface. These methods, collectively referred to as "physisorption", do not require the chemical modification of either entity. Physisorptive methods include electrostatic interactions, π-π stacking, hydrogen bonding, hydrophobic interactions, and van der Wals attractive forces.[1] Among these, electrostatic interactions and π-π stacking are more commonly employed.

Electrostatic interactions rely on the attraction of opposite charges. Due to the presence of aromatic amino acids and non-polar regions, many protein and peptide ligands are positively charged and readily interact with negatively charged substrates, such as silica or polymeric nanoparticles. Conversely, biomolecules such as DNA, RNA and acidic proteins carry negative charges and can interact with positively-charged substrates such as proteins. π-π stacking is a type of dispersion force (similar to van der Waals) that occurs when the π-electron clouds of aromatic rings overlap. A surprising number of materials are characterized by aromatic rings, including graphene, carbon nanotubes, and certain aromatic polymers. As a result, biomolecules containing aromatic amino acids (phenylalanine, tyrosine, and tryptophan) can adhere to these surfaces or other biomolecules.[31]

Despite its ease of use, physisorption is characterized by inherently random and weak attachments.[1] Not all biomedicine applications require the precise orientation of ligands, however many applications require more permanent bonds or interactions. In dynamic physiological environments such as the bloodstream, native proteins and biomolecules can displace weakly adsorbed substrates.[32]

Methods such as electrostatic interactions and hydrogen bonding are highly sensitive to natural changes in pH in ionic strength,[33] and the inherent reversibility of physisorptive interactions means that ligands can naturally desorb over time, leading to potential off-targets and limiting long-term applications such as sustained drug delivery. Although physisorption is well-suited to certain biomedical applications, this review emphasizes covalent and high-affinity techniques for the above reasons.

3. Glossary of Abbreviations and Acronyms

**Abbreviation or acronym**	**Long name**
AAC	Azide-alkyne cycloaddition
AAV	Adeno-associated virus
ADA	Alendronic acid
ADV	Adenovirus
AFM	Atomic force microscopy
AHA	L-azidohomoalanine
AI	Artificial intelligence
AM	Acetoxymethyl
ANN	Artificial neural network
APTES	3-Aminopropyltrimethoxysilane
Arg	Arginine
ATCC	American Tissue Culture Collection
ATP	Adenosine triphosphate
ATR	Attenuated total reflectance
B. subtilis	*Bacillus subtilis*
BCA	Bicinchoninic acid
BirA	Biotin protein ligase
BNC	Bacterial nanocellulose
BSA	Bovine serum albumin
bST	Bovine somatotropin ('bovine growth hormone')
BTNP	Barium titanate nanoparticle
BTO	Barium titanate
CADD	Computer aided drug design
CAR	Coxsackie-adenovirus receptor
CD	Circular dichroism
CDAP	1-Cyano-4-dimethylaminopyridinium
cDNA	Complementary DNA
Ce6	Chlorin e6
CFP	Cyan fluorescent protein
ChOx	3β-Hydroxysteroid oxygen oxidoreductase ('cholesterol oxidase')
ChR2	Channelrhodopsin-2
CMC	Carboxylmethyl chistosan
CNC	Cellulose nanocrystal
CNF	Cellulose nanofibril
CNT	Carbon nanotube
CodA	Cytosine deaminase
COF	Covalent organic framework
CP	Coat protein
CQD	Carbon quantum dot
CRISPR	Clustered regularly interspaced short palindromic repeats
CSP	Chiral stationary phase
CuAAC	Copper-catalyzed azide-alkyne cycloaddition

Cy3	Cyanine 3
Cy5	Cyanine 5
Cy5.5	Cyanine 5.5
Cys	Cysteine
DAPI	4',6-Diamidino-2-phenylindole
DBA	4,40-Diamino-3,30-biphenyldicarboxylic acid
DC-SIGN	Dendritic cell-specific intercellular adhesion molecule-3-grabbing non-integrin
DCC	Dicyclohexylcarbodiimide
DET	Direct electron transfer
DFT	Density functional theory
DLS	Dynamic light scattering
DMAP	4-Dimethylaminopyridine
DMSO	Dimethyl sulfoxide
DNA	Deoxyribose nucleic acid
DOX	Doxorubicin
DSC	Differential scanning calorimetry
DTT	Dithiothreitol ('Cleland's reagent')
DWCNT	Double-walled carbon nanotube
EC	European Comission
EDA	Ethylenediamine
EDC	1-Ethyl-3-(3-dimethylaminopropyl)carbodiimide
EDX (also EDS, EDXS or XEDS)	Energy dispersive x-ray spectroscopy
EGFR	Epidermal growth factor receptor
EIS	Electrochemical impedance spectroscopy
ELISA	Enzyme-linked immunosorbent assay
EMA	European Medicines Agency
EpCAM	Epithelial cell adhesion molecule
ERK	Extracellular signal-regulated kinase
E. coli	*Escherichia coli*
EUNCL	European Nanomedicine Characterization Laboratory
FDA	Food and Drug Administration
$FeCl_3{\cdot}6H_2O$	Ferric chloride hexahydrate
FITC	Fluorescein isothiocyanate
FOL	Folate
FRET	Förster resonance energy transfer
FTIR	Fourier transform infrared
GAG	Genetic algorithm
GalNAc	N-acetylgalactosamine
GIRK2	G-protein-coupled inwardly rectifying potassium channel 2
GLP-1	Glucagon-like peptide 1
GO	Graphene oxide
GOx	Glucose oxidase
GQD	Graphene quantum dot
HCY	Homocysteine

HDR	Homology-directed repair
HER2	Human epidermal growth factor receptor 2
Hf	Hafnium
HLA	Human leukocyte antigen
HNC	Hairy bacterial nanocellulose
HPLC	High pressure liquid chromatography
HRP	Horseradish peroxidase
HTA	2-Hydroxybenzene-1,3,5-tricarbaldehyde
HUVEC	Human umbilical vein endothelial cell
IC_{50}	Inhibitory concentration 50%
ICH	International Council for Harmonization of Technical Requirements for Pharmaceuticals for Human Use
IFE	Inner filter effect
IgG	Immunoglobulin G
IgG2b	Immunoglobulin G subclass 2b
KPFM	Kelvin probe force microscopy
LacZ	Beta-galactosidase
LAP	LplA acceptor peptide
LOD	Limit of detection
LOQ	Limit of quantification
LplA	Lipoic acid protein ligase A
LRET	Luminescence resonance energy transfer
LSPR	Localized surface plasmon resonance
MALDI-TOF	Matrix-assisted laser desorption ionization-time of flight
MES	2-(N-Morpholino)ethanesulfonic acid
MeTz3	3-Hydroxymethyl-6-methyl tetrazine
miR	MicroRNA
ML	Machine learning
MOF	Metal organic famework
MPTMS	(3-Mercaptopropyl)trimethoxysilane.
mTG	Microbial transglutaminase
MTS	3-(4,5-Dimethylthiazol-2-yl)-5-(3-carboxymethoxyphenyl)-2-(4-sulfophenyl)-2H-tetrazolium
MTT	(3-(4,5-Dimethylthiazol-2-yl)-2,5-diphenyltetrazolium bromide
MWCNT	Multi-walled carbon nanotubes
nano-QSAR	Nano-quantitative structure-activity relationship
NFSOM	Near-field scanning optical microscopy
NH2-BDC	2-Aminobenzene-1,4-dicarboxylic acid
NHS	N-hydroxysuccinimide
NIR	Near-infared
NLS	Nuclear localization sequence
NMR	Nuclear magnetic resonance
Npys	Nitropyridyl
NR	Neutron reflectivity (or 'neutron reflection')
NTA	Nanoparticle tracking analysis

PBS	Phosphate-buffered saline
PDI	Perylene diimide
PDMS	Polydimethylsiloxane
PEG	Polyethylene glycol
PEI	Polyethylenimine
PLL	ε-Poly-L-lysine
PTK7	Protein tyrosine kinase 7
Ppa	Pyropheophorbide-a
PPAA	Plasma polymerized acrylic acid
PPC	Preferred Product Characteristic
QCE	Quantum confinement effect
QCM	Quartz crystal microbalance
QD	Quantum dot
RMM	Resonant mass measurement
ROS	Reactive oxygen species
SEM	Scanning electron microscope/microscopy
SERS	Surface enhanced Raman spectroscopy
SHG	Second harmonic generation
SHRIMP	Second harmonic radiation imaging probe
siRNA	Small interfering RNA
SIRM	Stress-induced rolling membrane
SMC	Smooth muscle cell
SMCC	Succinimidyl 4-(N-maleimidomethyl)cyclohexane-1-carboxylate
SmPC	Summary of Product Characteristics
SNAr	Nucleophilic aromatic substitution
SPAAC	Strain-promoted azide-alkyne cycloaddition
SPCE	Screen printed carbon electrode
SPDP	Succinimidyl 3-(2-pyridyldithio)propionate
SPR	Surface plasmon resonance
S. aureus	*Staphylococcus aureus*
Sulfo	Sulfonated
SV40	Simian virus 40
SWCNT	Single-walled carbon nanotube
TAL	Transcription activator-like
TALE	Transcription activator-like effectors
TALEN	Transcription activator-like effector nucleases
TCEP	Tris(2-carboxyethyl)phosphine
TCO	Trans-cyclooctane
TEM	Transmission electron microscope/microscopy
TEMPO	2,2,6,6-Tetramethylpiperidine-1-oxyl
TGA	Thermogravimetric analysis
Tgase	Transglutaminase
TM	Transmembrane
TMB	3,3',5,5'-Tetramethylbenzidine

TMV	Tobacco mosaic virus
ToF-SIMS	Time-of-flight secondary ion mass spectrometry
tPA	Tissue plasminogen activator
TPP	Target Product Profile
TSS	Technical Specification Series
TWCNT	Triple-walled carbon nanotube
UCNP	Upconversion nanoparticle
UKHSA	United Kingdom Health Security Agency
UV	Ultraviolet
VMD	Visual molecular dynamics
VLP	Virus-like particle
WHO	World Health Organization
XPS	X-ray photoelectron spectroscopy
ZFN	Zinc-finger nuclease

4. References

1. Zhang L, Mazouzi Y, Salmain M, Liedberg B, Boujday S. Antibody-Gold Nanoparticle Bioconjugates for Biosensors: Synthesis, Characterization and Selected Applications. *Biosensors and Bioelectronics*. 2020;165:112370. doi:10.1016/j.bios.2020.112370

2. Alliegro MC. Effects of Dithiothreitol on Protein Activity Unrelated to Thiol–Disulfide Exchange: For Consideration in the Analysis of Protein Function with Cleland's Reagent. *Analytical Biochemistry*. 2000;282(1):102–106. doi:10.1006/abio.2000.4557

3. Huang X, Wu L, Hsu J, Shigeto S, Hsu H. Biothiol-triggered, self-disassembled silica nanobeads for intracellular drug delivery. *Acta Biomaterialia*. 2015;23:263–270. doi:10.1016/j.actbio.2015.05.006

4. Lahnsteiner M, Kastner A, Mayr J, Roller A, Keppler BK, Kowol CR. Improving the Stability of Maleimide–Thiol Conjugation for Drug Targeting. *Chemistry A European J*. 2020;26(68):15867. doi:10.1002/chem.202003951

5. Neri-Cruz CE, Teixeira FME, Gautrot JE. A guide to functionalisation and bioconjugation strategies to surface-initiated polymer brushes. *Chem Commun*. 2023;59(49):7534. doi:10.1039/d3cc01082a

6. Sun Kang M, See Kong TW, Xin Khoo JY, Loh T. Recent developments in chemical conjugation strategies targeting native amino acids in proteins and their applications in antibody–drug conjugates. *Chemical Science*. 2021;12(41):13613–13647. doi:10.1039/D1SC02973H

7. Hu C, Wang J. Chapter Five - Method for Enzyme Design with Genetically Encoded Unnatural Amino Acids. In: Pecoraro VL, ed. *Methods in Enzymology* Vol 580. Academic Press; 2016:109–133. https://doi.org/10.1016/bs.mie.2016.06.005. Accessed Dec 12, 2024

8. Zhang M, June SM, Long TE, Kong J. Principles of Step-Growth Polymerization (Polycondensation and Polyaddition). In: Principles of Step-Growth Polymerization (Polycondensation and Polyaddition). In: *Reference Module in Materials Science and Materials Engineering*Elsevier; 2016. https://doi.org/10.1016/B978-0-12-803581-8.01410-7. Accessed Dec 12, 2024

9. Fantoni NZ, El-Sagheer AH, Brown T. A Hitchhiker's Guide to Click-Chemistry with Nucleic Acids. *Chem Rev*. 2021;121(12):7122–7154. doi:10.1021/acs.chemrev.0c00928

10. Ghiassian S, Yu L, Gobbo P, et al. Nitrone-Modified Gold Nanoparticles: Synthesis, Characterization, and Their Potential as 18F-Labeled Positron Emission Tomography Probes via I-SPANC. *ACS Omega*. 2019;4(21):19106–19115. doi:10.1021/acsomega.9b02322

11. Pickens CJ, Johnson SN, Pressnall MM, Leon MA, Berkland CJ. Practical Considerations, Challenges, and Limitations of Bioconjugation via Azide–Alkyne Cycloaddition. *Bioconjugate Chem*. 2017;29(3):686. doi:10.1021/acs.bioconjchem.7b00633

12. Salic A, Mitchison TJ. A chemical method for fast and sensitive detection of DNA synthesis in vivo. *Proceedings of the National Academy of Sciences*. 2008;105(7):2415–2420. doi:10.1073/pnas.0712168105

13. Ahmad Fuaad AAH, Azmi F, Skwarczynski M, Toth I. Peptide Conjugation via CuAAC 'Click' Chemistry. *Molecules*. 2013;18(11):13174. doi:10.3390/molecules181113148

14. Rao H, Sawant AA, Tanpure AA, Srivatsan SG. Posttranscriptional chemical functionalization of azide-modified oligoribonucleotides by bioorthogonal click and Staudinger reactions. *Chem Commun*. 2012;48(4):498–500. doi:10.1039/C1CC15659D

15. Lepori I, Oz Y, Im J, et al. Bioorthogonal "Click" Cycloadditions: A Toolkit for Modulating Polymers and Nanostructures in Living Systems. *Reactions*. 2024;5(1):231. doi:10.3390/reactions5010010

16. Fantoni NZ, El-Sagheer AH, Brown T. A Hitchhiker's Guide to Click-Chemistry with Nucleic Acids. *Chem Rev*. 2021;121(12):7122. doi:10.1021/acs.chemrev.0c00928

17. Agard NJ, Prescher JA, Bertozzi CR. A Strain-Promoted [3 + 2] Azide−Alkyne Cycloaddition for Covalent Modification of Biomolecules in Living Systems. *J Am Chem Soc*. 2004;126(46):15046–15047. doi:10.1021/ja044996f

18. Kechkeche D, El Mousli S, Poujouly C, et al. Strain promoted azide alkyne cycloaddition, an efficient surface functionalization strategy for microRNA magnetic separation. *Next Materials*. 2025;6:100409. doi:10.1016/j.nxmate.2024.100409

19. Gobbo P, Mossman Z, Nazemi A, et al. Versatile strained alkyne modified water-soluble AuNPs for interfacial strain promoted azide–alkyne cycloaddition (I-SPAAC). *J Mater Chem B*. 2014;2(13):1764–1769. doi:10.1039/C3TB21799J

20. Liu X, Gong P, Song P, et al. Rapid conjugation of nanoparticles, proteins and siRNAs to microbubbles by strain-promoted click chemistry for ultrasound imaging and drug delivery. *Polym Chem*. 2019;10(6):705–717. doi:10.1039/C8PY01721B

21. Dundas CM, Demonte D, Park S. Streptavidin–biotin technology: improvements and innovations in chemical and biological applications. *Appl Microbiol Biotechnol*. 2013;97(21):9343–9353. doi:10.1007/s00253-013-5232-z

22. Luong JHT, Male KB, Glennon JD. Biotin interference in immunoassays based on biotin-strept(avidin) chemistry: An emerging threat. *Biotechnol Adv*. 2019;37(5):634–641. doi:10.1016/j.biotechadv.2019.03.007

23. Fatima I, Rahdar A, Sargazi S, Barani M, Hassanisaadi M, Thakur VK. Quantum Dots: Synthesis, Antibody Conjugation, and HER2-Receptor Targeting for Breast Cancer Therapy. *Journal of Functional Biomaterials*. 2021;12(4). doi:10.3390/jfb12040075

24. Matsumoto T, Tanaka T, Kondo A. Enzyme-mediated methodologies for protein modification and bioconjugate synthesis. *Biotechnology Journal*. 2012;7(9):1137–1146. doi:10.1002/biot.201200022

25. Li Y, Kan Z, You Y, Gao X, Wang Z, Fu R. Exogenous transglutaminase improves multiple-stress tolerance in Lactococcus lactis and other lactic acid bacteria with glutamine and lysine in the cell wall. *Biotechnol Lett*. 2015;37(12):2467–2474. doi:10.1007/s10529-015-1942-x

26. Walper SA, Turner KB, Medintz IL. Enzymatic bioconjugation of nanoparticles: developing specificity and control. *Current Opinion in Biotechnology*. 2015;34:232–241. doi:10.1016/j.copbio.2015.04.003

27. Montanari E, Gennari A, Pelliccia M, et al. Tyrosinase-Mediated Bioconjugation. A Versatile Approach to Chimeric Macromolecules. *Bioconjugate Chem*. 2018;29(8):2550–2560. doi:10.1021/acs.bioconjchem.8b00227

28. Alam MK, El-Sayed A, Barreto K, Bernhard W, Fonge H, Geyer CR. Site-Specific Fluorescent Labeling of Antibodies and Diabodies Using SpyTag/SpyCatcher System for In Vivo Optical Imaging. *Mol Imaging Biol*. 2019;21(1):54–66. doi:10.1007/s11307-018-1222-y

29. England CG, Luo H, Cai W. HaloTag Technology: A Versatile Platform for Biomedical Applications. *Bioconjugate Chem*. 2015;26(6):975–986. doi:10.1021/acs.bioconjchem.5b00191

30. Hatlem D, Trunk T, Linke D, Leo JC. Catching a SPY: Using the SpyCatcher-SpyTag and Related Systems for Labeling and Localizing Bacterial Proteins. *International Journal of Molecular Sciences*. 2019;20(9):2129. doi:10.3390/ijms20092129

31. Jian MQ, Xie HH, Xia KL, Zhang YY. Chapter 15 - Challenge and Opportunities of Carbon Nanotubes. In: Peng H, Li Q, Chen T, eds. *Industrial Applications of Carbon Nanotubes*Elsevier; 2017:433–476. doi.org/10.1016/B978-0-323-41481-4.00015-0. Accessed Dec 13, 2024

32. Alvisi N, Vries R. Biomedical applications of solid-binding peptides and proteins. *Materials Today Bio*. 2023;19:100580. doi:10.1016/j.mtbio.2023.100580

33. Kooijman EE, Tieleman DP, Testerink C, et al. An Electrostatic/Hydrogen Bond Switch as the Basis for the Specific Interaction of Phosphatidic Acid with Proteins *. *Journal of Biological Chemistry*. 2007;282(15):11356–11364. doi:10.1074/jbc.M609737200
